# Plant-associated fungi co-opt ancient antimicrobials for host manipulation

**DOI:** 10.1126/sciadv.aec1406

**Published:** 2026-04-29

**Authors:** Fantin Mesny, Valentina Wolf, Ana López-Moral, Anton Kraege, Wilko Punt, Saifei Liu, Jinyi Zhu, Jiyeun Park, Yukiyo Sato, Bart P. H. J. Thomma

**Affiliations:** Institute for Plant Sciences, University of Cologne, Cluster of Excellence on Plant Sciences (CEPLAS), Cologne, 50674, Germany.

## Abstract

Evolutionary histories of effector proteins secreted by fungal pathogens to mediate plant colonization remain largely elusive. While most functionally characterized effectors modulate plant immunity, recent discoveries have revealed previously unknown functions in targeting host-associated microbiota. We now developed an antimicrobial activity predictor for effector candidates (AMAPEC) and identified a wealth of antimicrobial effectors, including many highly conserved ones—suggesting ancient evolutionary origins. Unexpectedly, several plant immunomodulatory effectors display antimicrobial activity. We propose that these evolved from ancestral antimicrobials while perhaps retaining their original functions. In addition to roles in suppressing host immunity, they may manipulate plant microbiota to promote colonization. We show that the *Verticillium dahliae* effector Vd424Y affects host microbiota during infection and, more recently, evolved to target plant cell nuclei to manipulate host immunity. Thus, we argue that microbial antagonism is a fundamental fungal effector function and suggest that fungi repurposed ancient antimicrobials to serve diverse roles during host-pathogen coevolution.

## Teaser

Fungal effectors may have evolved from ancient antimicrobials, shaping plant microbiota and immunity during infection.

## INTRODUCTION

Under continuous threat of microbial parasitism, organisms evolved immune systems to withstand pathogenic encounters (*1*–*4*). In turn, pathogens evolved strategies to interfere with immune responses to mediate host colonization, typically through the secretion of molecules of diverse nature that are collectively called “effectors.” These effectors target a wide variety of molecular mechanisms in planta, ultimately resulting in compromised host immunity and resilience. Plant-pathogenic fungi typically secrete effectors apoplastically, several of which may subsequently be taken up by host cells to reach intracellular destinations. Effectors that localize in the apoplast may perturb detection by immune receptors or disarm apoplastic defenses, whereas those that localize intracellularly in the cytoplasm or within particular organelles may modulate immune signaling or particular metabolic pathways. (*5*, *6*). In addition to manipulating these well-documented plant targets, effectors may also serve in microbial competition, as revealed by the recent identification of effectors with selective antimicrobial properties that suppress antagonistic plant microbiota members with disease-suppressive functions (*7*–*14*). It is generally accepted that effectors are the products of long coevolutionary “arms races” in which plants and pathogens aim to defend and overcome defenses, respectively (*1*, *3*). Although particular effectors may originate from gene divergent evolution through the accumulation of mutations that ultimately lead to novel gene functions, genome recombination or horizontal gene transfer during host adaptation (*15*–*18*), the evolutionary histories, and, more particularly, the molecular origins of most effectors remain enigmatic.

Here, we investigate evolutionary histories of effectors in the light of the recent discovery of antimicrobial effectors. As we reveal that many secreted antimicrobial proteins show broad conservation throughout the fungal kingdom, suggesting ancient origins, we hypothesize that effectors with functions in host physiology manipulation evolved from ancient antimicrobials. We validate this hypothesis by demonstrating that effectors with reported functions in host immunomodulation have ancestral antimicrobial properties that may serve in microbial antagonism during plant colonization. Thus, our study provides unprecedented insights into fungal evolution and the origins of key factors required for host colonization.

## RESULTS

### Accurate prediction of effector antimicrobial activity

**Fig. 1.**
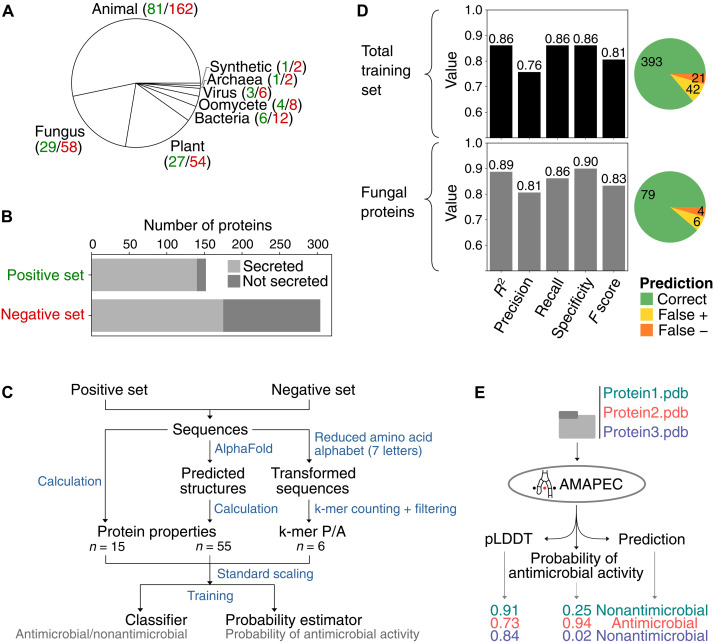
AMAPEC accurately predicts antimicrobial activities of fungal effector proteins. (**A**) Phylogenetic origin of the proteins in the dataset (green: number of proteins in positive dataset; red: number in negative dataset) used to train the predictor. (**B**) Number of proteins included in the training dataset and proportion of secreted proteins. (**C**) Diagram presenting a schematic overview of the training pipeline. In addition to physicochemical properties retrieved from protein sequences and structures, the pipeline considers the presence/absence (P/A) in protein sequences of six short motifs (k-mers) that are over- or underrepresented in the positive training set. These k-mers are encoded in a reduced amino acid alphabet, which groups amino acids according to their physicochemical properties (*n*, number of variables). (**D**) Estimation of the classifier quality, based on leave-one-out cross-validation in the training dataset. The top bar plot and pie chart show quality estimates calculated on the total dataset (*n* = 456), while the bottom charts analyze only the classifications of fungal proteins (*n* = 87) during the leave-one-out cross validation. (**E**) Schematic overview of AMAPEC v1.0 showing its inputs and outputs with an example of three proteins.

We first sought to identify previously unknown fungal effectors with antimicrobial activities. Machine learning classifiers have previously supported the identification of antimicrobial peptides (AMPs) that are shorter than 100 amino acids in length, based on their physicochemical properties (*19*–*21*). However, these are not suited to predict antimicrobial activities of effectors, as they are generally longer and form more complex structures (fig. S1). To train an adequate predictor, we curated a dataset of experimentally validated antimicrobial proteins from diverse organisms in the size range of effectors (35 to 642 amino acids, median = 125.5; Fig. 1, A and B, and table S1). Only few carbohydrate-active enzymes (CAZymes) were included since, although several families target microbial structures, it is often unknown whether individual members compromise microbial growth and can thus be classified as genuine antimicrobials. Thus, only few CAZymes could be included as bona fide antimicrobials. In addition, we curated a negative training set of equivalent proteins unlikely to have antimicrobial activity according to their functional annotation (table S2). For each protein in the training sets, we calculated properties from their amino acid sequences and predicted high-confidence protein structures (Fig. 1C and fig. S2), representing 70 numerical values reflecting diverse physicochemical properties (table S3). Moreover, we queried for the presence/absence of six k-mers that are over- or underrepresented in the sequences of the antimicrobial proteins (see Materials and Methods for details; table S3). All data were used to train a support vector machines (SVM) classifier and we subsequently estimated its quality through leave-one-out cross-validation, revealing that our classifier has high accuracy, recall, and specificity, particularly for fungal proteins (Fig. 1D). Analysis of support vector coefficients, representing the importance of individual properties for the prediction, revealed a role for hydrophobicity, charge, secondary structures, identity of exposed amino acids, disulfide bonds, and structural cavities (fig. S3). To confirm that our predictor can reliably identify previously unknown fungal antimicrobial proteins, we tested whether it correctly calls out seven more recently characterized fungal antimicrobials that were not included in the training set (*22*–*26*). As all seven proteins were correctly classified as antimicrobials (table S4), we implemented the predictor in the software package AMAPEC (antimicrobial activity prediction for fungal effector candidates; Fig. 1E), available at https://github.com/fantin-mesny/amapec (*27*).

**Fig. 2.**
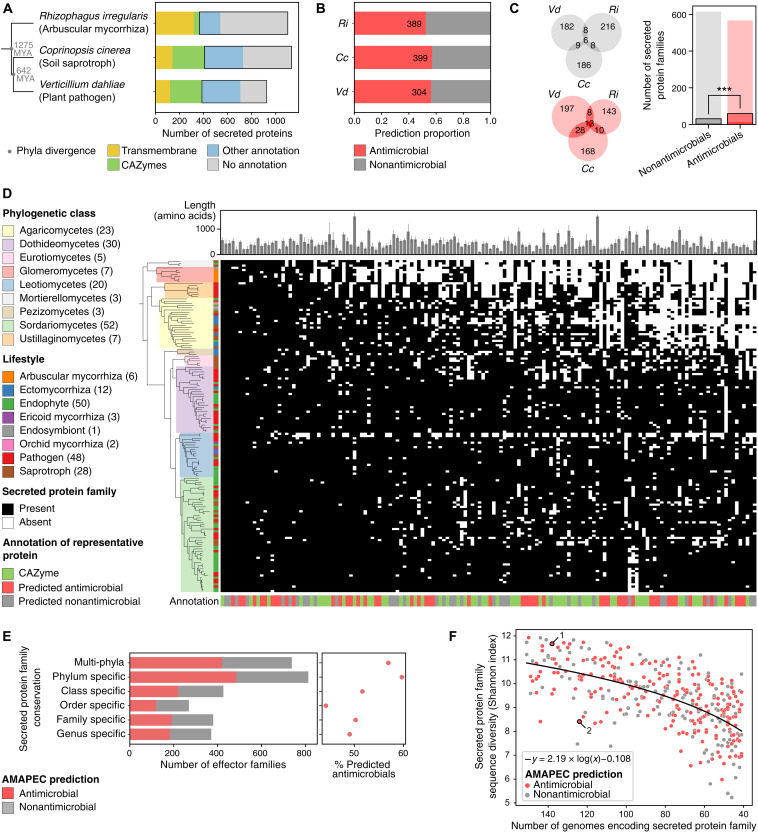
Predicted antimicrobials are abundant in fungal secretomes and exhibit high conservation. (**A**) Secretome functional annotation in three phylogenetically distant fungi with distinct lifestyles: *Rhizophagus irregularis* (*Ri*), *Coprinopsis cinerea* (*Cc*), and *Verticillium dahliae* (*Vd*). The phylogenetic tree [from phylot.biobyte.de, based on National Center for Biotechnology Information Taxonomy (*114*)] shows median taxa divergence times [TimeTree (*42*); MYA, million years ago]. Secreted proteins used for antimicrobial activity prediction are framed. (**B**) Proportions and counts of predicted antimicrobials in the three secretomes (excluding transmembrane proteins and CAZymes). (**C**) Conservation of predicted antimicrobials (red) and nonantimicrobials (gray) across the three secretomes according to orthology prediction. A bar plot displays protein family counts, with darker colors and dashed lines marking conserved fractions (i.e., presence in ≥2/3 fungi). Conserved effectors are overrepresented among antimicrobials (Fisher’s exact test: odds ratio = 2.19, *P* = 3.6 × 10^−4^). (**D**) Annotated phylogenetic tree (left) and heatmap (right) describing a diverse dataset of 150 fungal genomes and the presence/absence of the 150 most conserved secreted protein families in these fungi (according to orthology prediction), respectively. A bar plot above shows average protein lengths; colored bars below indicate CAZyme annotation or AMAPEC prediction (on one representative protein per family). (**E**) Numbers of predicted antimicrobial and nonantimicrobial families (prediction on representatives, excluding those annotated as transmembrane protein or CAZyme) across conservation levels in the 150-genome dataset. A scatterplot presents proportions of predicted antimicrobials along the same conservation levels, which follow a significantly descending trend (Cochran-Armitage test: stat. = 5129.0, *P* < 2.36 × 10^−5^). (**F**) Protein family sequence diversity [Shannon α diversity (*69*)] is plotted against decreasing conservation across 150 genomes. A logarithmic fit highlights the overall trend linking sequence diversity to conservation. Outlier datapoints above this fit may be under diversifying selection (accumulating mutations), whereas those below may be under purifying selection (preventing mutations to conserve ancestral functions). Datapoint colors indicate predicted antimicrobial activity. Families “1” and “2” include experimentally validated antimicrobials (*11*, *30*, *115*).

We used AMAPEC to identify candidate antimicrobial proteins in the secretomes of three phylogenetically distant fungi with distinct lifestyles, namely, the mycorrhizal glomeromycete *Rhizophagus irregularis*, the saprotrophic basidiomycete *Coprinopsis cinerea*, and the ubiquitous plant-pathogenic ascomycete *Verticillium dahliae* that causes vascular wilt disease in a wide diversity of host plants, including many crops (Fig. 2, A to C) (*28*). Functional compositions of fungal secretomes remain largely elusive, as they comprise a substantial proportion of proteins without functional annotations (Fig. 2A) and proteins for which the assigned annotations are poorly informative (tables S5 to S7). Although, in principle, the AMAPEC predictor can be used on CAZymes, we excluded these enzymes from the analysis to be rather conservative in our predictions, given the low number of CAZymes in our training set, which may lead to less robust predictions. Unexpectedly, AMAPEC prediction revealed that one-third to half of each of the secretomes without CAZymes of the three fungi is composed of putative antimicrobial proteins (Fig. 2B). Orthology analysis revealed that some antimicrobials are conserved across the three fungi, despite their wide phylogenetic spread, and thus ancestral to the three phyla (Fig. 2C). Moreover, antimicrobials show significantly greater conservation across species (i.e., occurrence of orthologs) than nonantimicrobials (Fig. 2C; Fisher’s exact test: odd’s ratio = 2.19, *P* = 3.6 × 10^−4^), suggesting that microbial antagonism is relatively ancient among secretome functions.

### Secreted antimicrobial proteins are conserved throughout the fungal kingdom

The evolution of antimicrobial proteins was further investigated in 150 genomes of diverse soil- and plant-associated fungi, spanning three phyla, nine classes, and 24 orders (fig. S4 and table S8). This dataset encompasses more than 700 million years of fungal evolution, whereas pathogenicity toward land plants only evolved after plants colonized land, about 500 million years ago. After classifying secreted proteins into sequence-related families through orthology prediction, antimicrobial activities were predicted for the most representative protein of each family (table S9). Many of the most conserved protein families, which occur in fungi with diverse lifestyles, were predicted as antimicrobials (Fig. 2D). We calculated the percentage of predicted antimicrobial families across different levels of conservation (Fig. 2E) and identified a significant decrease from 56.9% of multi-phyla families to 49.1% of genus-specific families (Cochran-Armitage test for trend: statistic = 5129.0, *P* < 2.36 × 10^−5^). This overrepresentation of predicted antimicrobials among the most conserved secreted protein families signifies their ancient origins, preceding fungal phyla divergence, and corroborates that fungi have relied on antimicrobials long before establishing symbioses with multicellular eukaryotes such as land plants and animals (*29*). While the most conserved secreted protein families expectedly exhibit the most diverse sequences, certain predicted antimicrobial families, including previously characterized ribonuclease-like antimicrobials (*11*, *30*), retained low sequence diversity during evolution, possibly to conserve their ancestral function (i.e., purifying selection; Fig. 2F). Together, our results demonstrate that microbial antagonism through the secretion of antimicrobial proteins is an ancient and conserved trait that likely supports fungal fitness across a wide diversity of habitats.

### Effectors of plant-pathogenic fungi display antimicrobial activities

**Fig. 3.**
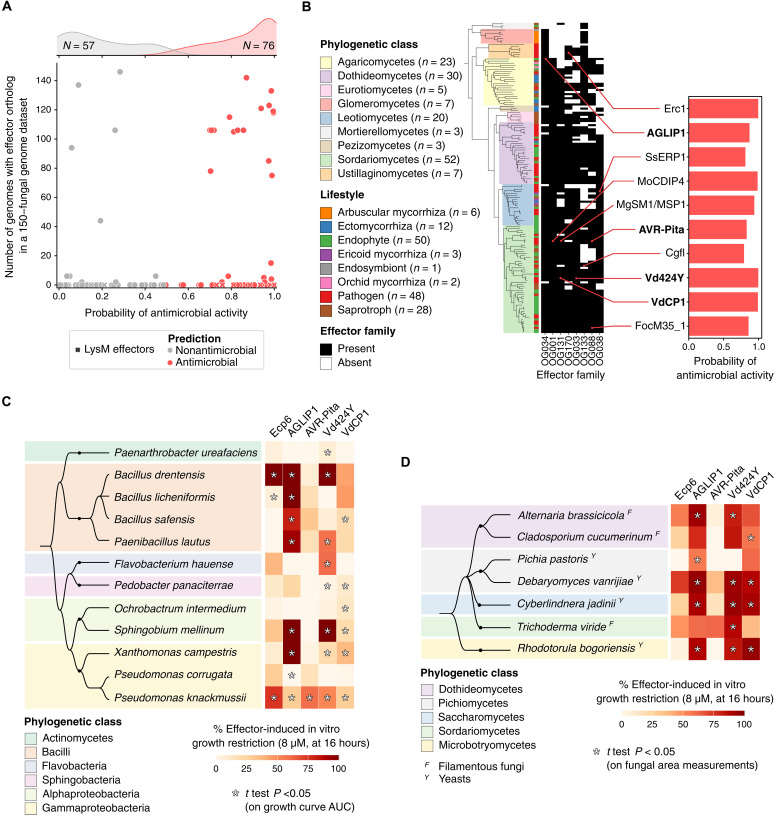
Immunomodulatory effectors also have antimicrobial properties. (**A**) Scatterplot showing results of antimicrobial activity prediction on fungal effectors from PHI-base (*37*) and the number of genomes containing their orthologs (of 150 fungal genomes) as a proxy for their conservation across the fungal kingdom. (**B**) Heatmap showing the presence/absence in 150 fungal genomes of eight secreted protein families including effectors previously demonstrated to act on plant hosts. These families were selected because they include at least one protein which is highly similar to a PHI-base effector (≥80% amino acid sequence identity), they show high conservation across the fungal kingdom (family represented in ≥100 of 150 genomes), and the associated reference effectors are predicted antimicrobials with highly confident structure prediction (mean pLDDT ≥ 70). A bar plot shows this antimicrobial activity prediction and the effector names. Names in bold highlight effectors selected for experimental validation of predicted antimicrobial activities. (**C**) Heatmap showing growth restriction by 8 μM effector protein of 12 bacteria, described with a species phylogeny on the left [Taxallnomy (*116*)]. Each heatmap column corresponds to a different fungal effector. Percentages of effector-induced growth restriction were calculated after 16 hours of growth. Asterisks indicate significant differences between from bacterial growth in the presence and in the absence of effector protein, identified with Student’s *t* tests on area under the curve (AUC) values. (**D**) Heatmap showing growth restriction by 8 μM effector protein of seven fungi (four yeasts, three filamentous fungi), described in the species phylogeny on the left [Taxallnomy (*116*)]. Each heatmap column corresponds to a different fungal effector. Percentages of effector-induced growth restriction were calculated after 16 hours of growth. Asterisks highlight significant differences between from fungal growth in the presence and in the absence of effector protein, identified with Student’s *t* tests. See figs. S6 to S15 for more details on the assays presented on (C) and (D).

We recently showed that *V. dahliae* secretes effector proteins with selective antimicrobial activity to manipulate resident microbiota at various life stages, including host colonization (*7*–*9*, *14*). We noticed that several *V. dahliae* effectors previously characterized to modulate host immunity have predicted antimicrobial properties (table S10), including VdCP1 (*31*), Vd424Y (also known as VdXyn4) (*32*, *33*), and Vd2LysM (*34*), a member of the LysM effector family which represses chitin-triggered plant immunity and has an ancient origin since family members occur throughout the fungal kingdom (*35*, *36*). On the basis of these findings, we hypothesized that plant-pathogenic fungi have evolved effectors to manipulate plant host physiology from ancestral antimicrobial proteins. In support of this hypothesis, we found that most functionally characterized effectors registered in the Pathogen-Host Interactions Database (PHI-base) (*37*) (*n* = 76 of 133), some of which are broadly conserved throughout our dataset of 150 genomes (Fig. 3B), have predicted antimicrobial properties (Fig. 3A and table S11). To validate these predictions, we subsequently selected five effectors for experimental validation: the LysM effector Ecp6 from the tomato leaf mould fungus *Cladosporium fulvum* (*38*), AGLIP1 from the root rot fungus *Rhizoctonia solani* (*39*), AVR-Pita from the rice blast fungus *Magnaporthe oryzae* (*40*), and Vd424Y (*32*, *33*) and VdCP1 (*31*) from *V. dahliae*. All proteins were heterologously produced in *Escherichia coli* and used in antimicrobial activity assays using a diverse set of plant-associated microbes including 12 bacteria that were isolated from tomato plants (*41*), as tomato is a host of three of the four above-mentioned fungi, four yeasts, and three filamentous fungi. Despite their well-characterized host targets, all five effectors exhibited antimicrobial activities in vitro with highly distinct activity spectra at micromolar concentrations (Fig. 3, C and D, and figs. S6 to S15). As homologs of all five proteins occur in fungi that do not live in association with plants, and most homologs in their protein families are predicted antimicrobials (fig. S5 and tables S12 to S16), antimicrobial activity is likely ancestral and predates the evolution of host manipulation. Overall, our data suggest that pathogen effectors evolved from ancient antimicrobial proteins that retain their ancestral functions while acquiring the ability to manipulate host physiology.

### A dual role of the effector Vd424Y in host manipulation and microbial antagonism

**Fig. 4.**
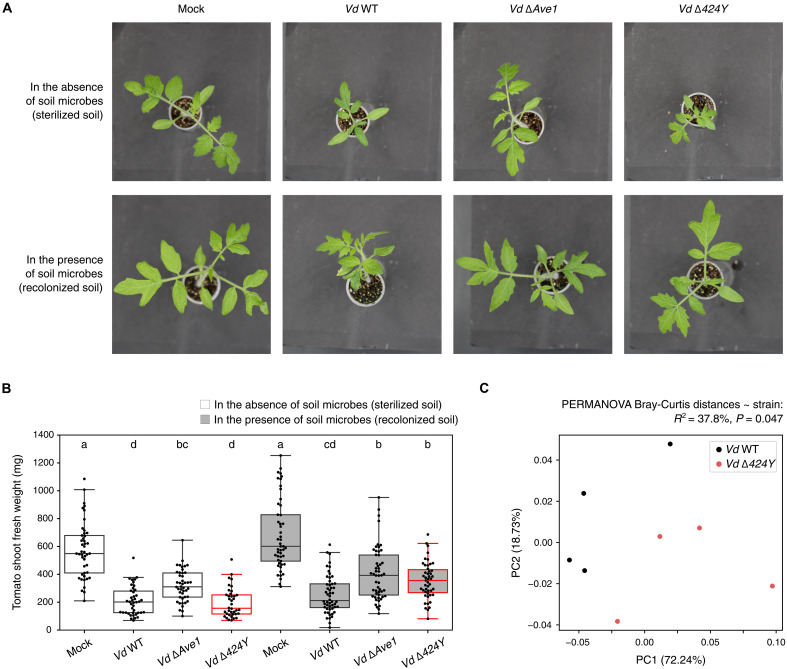
The effector Vd424Y acts in *V. dahliae* antagonism toward plant microbiota members. (**A**) Representative photographs of tomato plants upon mock or fungal inoculation in a gnotobiotic system in the absence (sterilized soil; top) or presence (sterilized soil recolonized with microbes derived from nonsterilized soil; bottom) of a soil microbiota. Fungal strains used for plant infections include wild-type *V. dahliae* (*Vd* WT), *Vd424Y* deletion mutant (*Vd* ∆424Y), and, as a control, an *Ave1* deletion mutant lacking the Ave1 effector previously demonstrated to contribute to *V. dahliae* virulence through both an antimicrobial activity targeting plant microbiota members and the manipulation of plant physiology (*7*, *41*). (**B**) Tomato shoot fresh weight values reflecting plant health after mock or fungal inoculation in a gnotobiotic system. Plants were grown axenically (sterilized soil; white boxes) or in the presence of microbes (recolonized soil; gray boxes). The *Vd424Y* deletion mutant (*Vd* ∆424Y) is highlighted with red box edges. Between 38 and 52, tomato plants were grown per condition, over three independent biological replicates. Letters on the boxplot highlight significant differences between treatments identified by ANOVA test (*P* < 0.05) followed by Tukey’s post hoc test (adjusted *P* < 0.05). (**C**) Principal coordinates analysis (PCoA) of Bray-Curtis dissimilarities between bacterial microbiota compositions of tomato plants inoculated with *Vd* WT or *Vd* ∆424Y. Results of a permutational multivariate analysis of variance (PERMANOVA) test revealing the significant difference of tomato microbiota compositions between *Vd* WT– and *Vd* ∆424Y–inoculated conditions are displayed above the PCoA.

Since many fungal effectors have evolutionary conserved ancestral antimicrobial properties (Fig. 3), we hypothesize that, in addition to manipulation of host physiology, they antagonize microbial competitors during infection. To address this hypothesis, we focused on the *V. dahliae* effector Vd424Y, which is known to exert xylanolytic and cytotoxic activities in planta (*32*, *33*). To investigate whether this effector also functions in microbial competition, we first measured effector gene expression in a diverse set of 10 soils, in the absence of a plant host (fig. S16). We found that *V. dahliae* expresses the *Vd424Y* gene in these soils, suggesting functions beyond host physiology manipulation. Next, we tested whether the antimicrobial activity of Vd424Y plays a role during plant colonization, by performing tomato inoculation experiments in a gnotobiotic system, allowing to study virulence contributions of effector genes in the presence and absence of host-associated microbiota (*41*). We identified a significant microbiota-dependent contribution of Vd424Y to *V. dahliae* virulence, as disease development was compromised upon *Vd424Y* deletion in the presence, but not in the absence of host-associated microbes (Fig. 4, A and B). This finding suggests that Vd424Y plays a role in microbiota manipulation during tomato plant infection, and thus that it retained its ancestral antimicrobial property throughout coevolution with plant hosts. To investigate this hypothesis further, we profiled the bacterial microbiota of the *V. dahliae* wild-type– and ∆*424Y*-infected tomato plants and found that their compositions differed significantly (permutational multivariate analysis of variance, *R*^2^ = 37.8%, *P* = 0.047; Fig. 4C). A differential abundance analysis enabled to identify several bacterial genera, including *Pseudoxanthomonas*, *Comamonas*, *Brachybacterium*, and *Sphingobium*, whose relative abundance is reduced in the presence of the *Vd424Y* gene (fig. S17A). Since the Vd424Y protein restricted the growth of a *Sphingobium* isolate in vitro (fig. S9), these bacterial genera may represent microbial targets of the effector in planta. We assessed whether the growth of *Pseudoxanthomonas* bacteria, whose abundance in planta was significantly reduced in the presence of *Vd424Y* (fig. S17A), is similarly restricted by Vd424Y in vitro. Although our results revealed notable intragenus variation, we found that Vd424Y is able to inhibit particular *Pseudoxanthomonas* strains (fig. S17B). Thus, we conclude that Vd424Y plays a role in microbiota manipulation through direct antimicrobial activity.

### Vd424Y evolved to manipulate plant physiology by locating to host cell nuclei

**Fig. 5.**
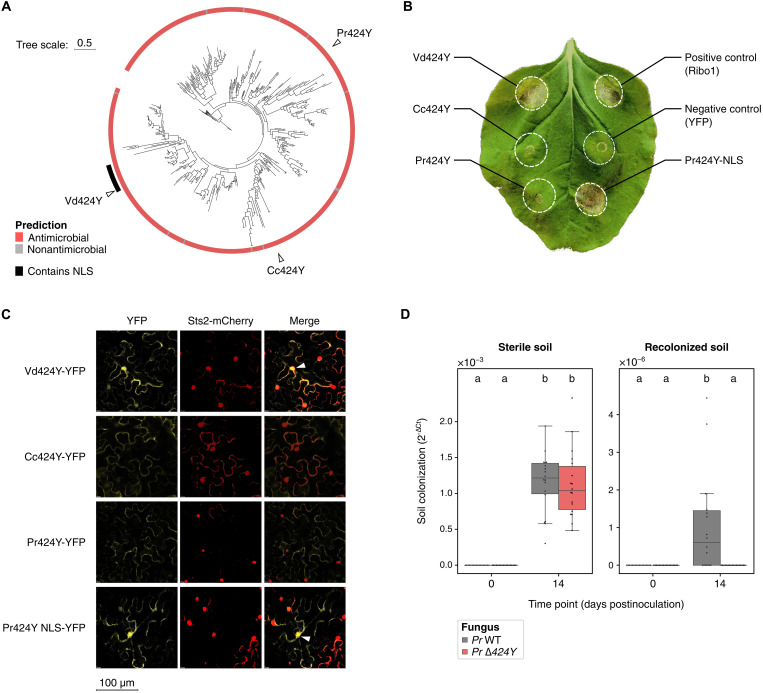
Evolutionary and functional analysis of the Vd424Y family. (**A**) Maximum-likelihood phylogenetic tree [IQ-TREE (*89*), model LG] of Vd424Y homologs in a dataset of 150 fungal genomes (as in fig. S5), supplemented with Vd424Y orthologs identified in the genomes of *C. cinerea* (Cc424Y) and *P. restrictum* (Pr424Y). The tree is annotated with antimicrobial activity prediction results as well as the occurrence of an NLS, annotated using cNLS Mapper (*94*). NLS sequences annotated in the effector family are all identical (fig. S17B). (**B**) Representative *N. benthamiana* leaf in which proteins were transiently expressed by *Agrobacterium*-mediated transformation. Infiltrated leaf areas are circled by dotted lines and annotated with the names of transiently expressed genes. In addition to *Vd424Y*, *Cc424Y*, *Pr424Y*, the positive control *Ribo1* (*11*), and the negative control *YFP*, a chimeric variant of *Pr424Y* that carries the NLS of *Vd424Y* (Pr424Y-NLS) was transiently expressed. See fig. S19 for photographs of experimental replicates. (**C**) Fluorescent confocal microscopy images displaying the localization of effector proteins fused to the yellow fluorescent protein (YFP) in *N. benthamiana* leaf cells; from top to bottom: Vd424Y, Cc424Y, Pr424Y, and Pr424Y-NLS. While the first column shows effector localization (YFP signal), the second displays localization of the mCherry-tagged nuclear localized effector protein Sts2 as a positive control (*110*). The third column shows merged signals with white arrowheads pointing to cell nuclei in which both effectors were detected. (**D**) Soil colonization by wild-type *P. restrictum* (*Pr* WT) and a *Pr424Y* gene deletion mutant (*Pr* ∆424Y), in sterilized soil (left) and sterilized soil that was recolonized with soil microbes (right). Colonization was quantified by real-time PCR using species-specific primers and normalization to an artificial spike-in plasmid added before DNA extraction. Letters indicate significant differences according to Kruskal-Wallis (*P* < 0.05) and post hoc Dunn tests (adjusted *P* < 0.05).

Vd424Y was previously demonstrated to localize into chloroplasts and nuclei of host cells and, accordingly, carries a chloroplast transit peptide (cTP) and a nuclear localization signal (NLS) (*32*, *33*). While the cTP is sparsely detected throughout the Vd424Y family (fig. S18A) and shows a high degree of sequence variation (fig. S18B), the occurrence of the NLS is restricted to a single clade that is exclusively composed of proteins secreted by plant pathogens and endophytes from the Sordariomycete genera *Verticillium*, *Fusarium*, and *Cylindrocarpon* (Fig. 5A and fig. S18A), which diverged about 250 million years ago (*42*). This observation suggests that, contrary to antimicrobial properties, the plant cell nuclear localization that is required for immunomodulation and cytotoxicity in planta (*32*) is a trait that emerged recently in the family of Vd424Y homologs. We hypothesized that homologs of Vd424Y from saprotrophic fungi do not exert immunomodulatory and cytotoxic effects in planta, given that these fungi do not typically live in association with plants and that these traits are mediated by the NLS. To address this hypothesis, we selected two orthologs of Vd424Y, secreted by the basidiomycete *C. cinerea* and the ascomycete *Penicillium restrictum*, that we named Cc424Y and Pr424Y, respectively. Following *Agrobacterium*-mediated transient effector gene expression in *Nicotiana benthamiana* leaves, we observed that Vd424Y caused cell death, in line with previous reports (*32*, *33*). The Cc424Y and Pr424Y homologs did not cause cell death in *N. benthamiana* (Fig. 5B and fig. S19). This finding corroborates the recent evolution of plant immunomodulatory functions in the 424Y effector family, as it highlights that (a relatively recent ancestor of) *V. dahliae* repurposed this ancient antimicrobial protein for host manipulation. We furthermore tested whether the acquisition of the NLS is sufficient for such neofunctionalization by engineering a chimeric *Pr424Y* variant that comprises the NLS of *Vd424Y* (named *Pr424Y-NLS*). Notably, *Agrobacterium*-mediated expression of this variant in *N. benthamiana* leaves resulted in clearly visible cell death, showing that the *P. restrictum* 424Y homolog gained the capability to manipulate host physiology (Fig. 5B). Through fluorescent confocal microscopy, we observed that Vd424Y localized to the nuclei of *N. benthamiana* leaf cells upon transient gene expression, in contrast to Cc424Y and Pr424Y. However, like Vd424Y, also the artificially engineered Pr424Y variant that contains the NLS of Vd424Y could be found in the nucleus, confirming that cell death induction depends on the nuclear localization of the effector (Fig. 5C). Together, these results suggest that the recent evolution of an NLS underlies the host immunomodulatory functions of Vd424Y by mediating its localization in host nuclei.

We lastly hypothesized that in the absence of an NLS, Vd424Y homologs support diverse fungal lifestyles by serving functions in microbial antagonism. We tested whether Pr424Y contributes to fungal niche colonization by comparing the capacity of *P. restrictum* wild-type and a Pr∆*424Y* gene deletion mutant to colonize soil in the presence and absence of a resident microbiota. Concordant with a role in microbial competition, no significant difference in colonization was observed following inoculation of *P. restrictum* in sterilized soil (Fig. 5D). However, although fungal colonization in natural soil was greatly reduced when compared with growth in sterilized soil due to the presence of microbial competitors, in the presence of a soil-associated microbiota, Pr∆*424Y* mutants displayed a significantly reduced soil colonization when compared with wild-type *P. restrictum*. This finding suggests that ancient antimicrobial properties of 424Y proteins underlie diverse fungal lifestyles by supporting the colonization of various niches through microbial antagonism.

## DISCUSSION

Before plants colonized land about 500 million years ago, fungi lived as saprotrophs and/or parasites of bacteria and primitive algae in (semi)aquatic environments (*43*). The study of a 600 million year–old fossil found evidence of a symbiosis between a fungus and an auxotrophic organism, highlighting the ancient origins of fungi-plant interactions (*44*). However, pathogenicity toward land plants represents a relatively recent trait in the 1.5 billion year-long fungal evolutionary history, which evolved multiple times independently across the fungal tree of life (*45*). Effector-based strategies to manipulate host physiology are essential for the pathogenic lifestyle (*5*, *6*), yet their evolutionary origins remained elusive. The broad conservation of certain effector families throughout the fungal kingdom (Fig. 3B), together with their occurrence in fungi that do not colonize plants, demonstrates ancient origins of these effectors and suggests their primary roles beyond plant host manipulation. Since antimicrobial effectors are likely secreted upon encountering plant microbiota epiphytically and in the apoplast of host tissues, they may be particularly prone to evolve functions to manipulate host physiology through interactions with host surface and apoplastic proteins. However, our findings that underpin an evolutionary history of Vd424Y through the acquisition of a NLS (Fig. 5) demonstrates that some antimicrobial effectors acquired in planta functions by evolving intracellular localization. Thus, many effector proteins with diverse in planta modes of action have ancestral antimicrobial properties, indicating that microbial antagonism is a fundamental effector role that likely supported fungal fitness in (semi)aquatic environments before the evolution of fungal pathogenicity toward land plants (*29*). In line with this discovery, previous studies identified peptides with dual functions in microbial antagonism and immunity modulation, suggesting that these two protein activities may often co-occur given their functional complementarity (*46*, *47*). Moreover, a recent study identified that changes in protein domain organization may have repurposed an effector from antimicrobial to immunomodulator, thereby mediating a recent fungal lifestyle transition from saprotroph to plant symbiont (*48*). Hypothetically, the co-option of antimicrobial proteins for host manipulation mediated the compatibility of the first fungal symbioses with land plants and predates the evolution of a plethora of other molecular mechanisms that underlie more specific pathogenic interactions. Some fungal effectors represent recent innovations, as evidenced by their lineage specificity, and likely result from fungal adaptation to specific plant lineages (*15*, *49*). Such effectors are generally thought of as products of coevolutionary arms races in which plant immune systems and fungi aim to detect and overcome detection, respectively (*1*, *3*). While this study focuses on the occurrence of antimicrobial properties among the most conserved effector families, it paves the way for further research on effector evolution, particularly to investigate how recently evolved antimicrobial effectors may mediate host and niche adaptation (*50*). More generally, this study, and more particularly the AMAPEC software, will support the discovery ofpreviously unknown fungal antimicrobial proteins and assist studies on their modes of action, which now remain largely unknown. Furthermore, the broad variety of fungal secreted antimicrobials and their crucial role in supporting host colonization are important factors to consider when designing novel biocontrol strategies to protect crops efficiently from pathogenic fungi. Last, the evolutionary trajectories identified in this study are likely to be relevant beyond plant colonization, given the need for human- and animal-pathogenic fungi to manipulate immune responses of their hosts during infection too (*51*).

## MATERIALS AND METHODS

### Curation of a training dataset for antimicrobial activity prediction

To develop the AMAPEC predictor, a positive training set of antimicrobial proteins (table S1) was curated from literature. Only proteins for which antimicrobial activity has been experimentally demonstrated in vitro (i.e., restricting the growth of bacteria and/or fungi in culture medium) were selected. While not restraining the dataset to proteins encoded by any phylogenetic group, we paid particular attention to include all the fungal antimicrobial proteins reported in scientific literature. Secretion signal peptides were removed from sequences [SignalP v6.0 (*52*)], since the antimicrobial function of proteins occurs after secretion. Considering sequence lengths of fungal secreted proteins, peptides with mature sequence lengths below 35 amino acids were excluded (fig. S1), not to enrich the protein set in AMPs, for which dedicated predictors exist (*19*, *53*–*57*). By largely spanning the size range of typical effector proteins, this protein set should support the prediction of effector antimicrobial activity without bias toward the recognition of short AMPs, which are the most described antimicrobial proteins in the literature.

A negative training dataset was assembled by gathering presumable nonantimicrobial proteins (table S2). As previously suggested (*19*, *54*), this negative set was curated by retrieving proteins which functional annotation does not suggest any antimicrobial activity from the UniProt database (*58*). To do so, Gene Ontology (GO) terms associated to antimicrobial activity were filtered out (i.e., GO:0090729, GO:0001878, GO:0045087, GO:0050830, GO:0050829, GO:0042742, GO:0071222, GO:0071224, GO:0001530, GO:0031640, and GO:0050832). In addition, only well-annotated proteins without any known function in microbial antagonism or immunity were selected. To prevent strong effects of potential misjudgment during the curation process, the negative training dataset includes twice as many nonantimicrobial proteins as there are antimicrobials in the positive set. For each antimicrobial in the positive set, two presumably nonantimicrobial proteins encoded by the same organism (or a close relative) and with similar sizes (±4 amino acids) were included in the negative set. We paid attention to include at least as many secreted proteins [signal peptide detected and removed with SignalP (*52*)] in the negative set as in the positive set, not to bias the prediction toward apoplastically released proteins. Last, since 11 proteins in the positive training set were annotated or described as ribonucleases, 11 ribonucleases, unlikely to exert antimicrobial functions according to their annotation (for instance, involved in transfer RNA maturation), were included in the negative set.

### Calculation of protein properties

The AMAPEC predictor was trained on a set of 70 numerical variables reflecting protein physicochemical properties (table S3). Some of these values (*n* = 15) were calculated from amino acid sequences, using R v4.2.0 and the library Peptides v2.4.4 (*59*). However, to better describe the physicochemistry of proteins, their predicted structures were used to calculate 55 numerical values per protein that reflect structural properties. Protein structures were predicted using AlphaFold v2.0 (*60*) with parameters “--max_template_date=2021-05-14 –preset=casp14.” Structure properties were calculated from AlphaFold best models (“ranked_0.pdb” output files) using Python v3.11.5 and the protein database file format parser implemented in Biopython v1.78 (*61*). Some previously published code and formula from diverse sources (*62*–*64*) (details in table S3) were implemented in AMAPEC’s Python scripts. For properties linked to protein secondary structures, DSSP v3.0.0 (*65*, *66*) was used to assign individual amino acids to different types of secondary structures. In addition, pocket structures in proteins were predicted using Fpocket v4.0.2 (*67*), and information related to their number, size, and properties was implemented as variables.

In addition to sequence- and structure-derived physicochemical properties, the presence/absence of certain k-mers in the protein sequences was implemented as variable. To reduce sequence complexity, a reduced amino acid alphabet was used, as previously implemented in various machine learning methods applied to protein sequences (*54*, *68*). A novel seven-letter alphabet based on amino acid properties was designed, to define k-mers that may represent key motifs in protein physicochemistry: small amino acids (G and A) were mapped to “0,” nucleophilic amino acids (S, T, and C) were mapped to “1,” hydrophobic amino acids (V, L, I, M, and P) were mapped to “2,” aromatic amino acids (F, Y, and W) were mapped to “3,” acidic amino acids (D and E) were mapped to “4,” amide amino acids (N and Q) were mapped to “5,” and basic amino acids (H, K, and R) were mapped to “6.” The k-mer compositions of transformed sequences in the training set was profiled using MerCat2 v1.0 (*69*), with k-mer sizes 3, 4, 5, and 6 amino acids. Then, chi-square multiple testing was computed, as implemented in function feature_selection.SelectFdr(chi2, alpha=0.05) of Python library scikit-learn v1.2.1 (*70*), to identify k-mers that are over- or underrepresented in antimicrobial protein sequences. This aimed to select for k-mers that are likely biologically meaningful and to prevent later overfitting of our prediction model that can occur if training is performed on numerous variables which combination describes protein sequences in too much detail. With chi-square testing, six k-mers (five 4-mers and one 3-mer) of interest were identified. Their presence/absence was implemented in the set of protein properties used to train the AMAPEC predictor (table S3).

### Classifier training and quality estimation

Numerical variables reflecting properties of our 456 proteins were standardized using function preprocessing.StandardScaler() of Python library scikit-learn v1.2.1 (*70*). Then, a SVM classifier with a linear kernel was trained using function svm.SVC() from scikit-learn. The correct the imbalance of the training set (152 proteins in the positive set and 304 in the negative set), the weight of antimicrobials was set to 2 and the weight of nonantimicrobials to 1. A second model was trained to predict the probability of antimicrobial activity, by computing Platt scaling over the SVM binary classifier. To do so, the function calibration.CalibratedClassifierCV(method=“sigmoid”, cv=“prefit”) from sci-kit learn was used. Both models were exported using function dump() from Python library joblib v1.2.0.

Because of the small size of the training dataset (*n* = 456), classifier quality testing was performed through leave-one-out cross-validation. As implemented in the function cross_val_score[cv=KFold(n_splits=456)] of scikit-learn, 456 SVM classifiers were trained with a train/test split of 455/1 to classify individual proteins using as a basis, protein properties in the rest of the dataset. Protein classifications into “antimicrobial” or “nonantimicrobial” were then analyzed by counting the numbers of true positives, false positives, true negatives, and false negatives. These counts allowed not only the estimation of the overall classifier accuracy (*R*^2^) but also its precision, recall, specificity, and *F* score. Such quality estimates were also calculated by exclusively taking the classification correctness of fungal proteins into account, to identify if the predictor is suited for the annotation of fungal proteins. Last, the predictor was tested on seven recently characterized fungal proteins demonstrated to have antimicrobial activities (*22*–*26*), after structure prediction with ESMFold v1.0.3 (table S4) (*71*).

A bash pipeline allowing both the calculation of protein properties and antimicrobial activity prediction using the trained predictors was written, resulting in the software AMAPEC v1.0 (https://github.com/fantin-mesny/amapec) (*27*), developed and tested on operating system GNU/Linux Ubuntu v20.04.3 LTS.

### Secretome analysis on three phylogenetically distant fungi

Sets of proteins associated to the published genomes of three phylogenetically distant fungi with distinct lifestyles were downloaded: *V. dahliae* JR2 (*72*) [annotation VDAG_JR2 v.4.0 downloaded from the database Ensembl Fungi (*73*)], *C. cinerea* AmutBmut pab1-1 (*74*) [annotation *Copci_AmutBmut1 v1.0* downloaded from the database Joint Genome Institute Mycocosm (*75*)], and *R. irregularis* DAOM197198 (*76*). SignalP v6.0 (*52*) was then used to predict secretion signal peptides in protein sequences and thereby define the secretomes of these fungi. Sequences with removed signal peptides were used in all subsequent analyses. Functional annotation of proteins in these secretomes was carried out using emapper v2.0 (*77*) and the database EggNog v5 (*78*). CAZymes and transmembrane proteins were specifically annotated in these secretomes using dbCAN v4.0 and TMBed v1.0.0, respectively (*79*, *80*). Structure predictions were computed with ESMFold v1.0.3 (*71*), using default parameters, of all secreted proteins besides CAZymes. The structures of two proteins from *V. dahliae* (VDAG_JR2_Chr4g10970 and VDAG_JR2_Chr1g22375) could not be predicted due to high computational requirements linked to their size (>2500 amino acids) and were excluded from our analyses. To validate that the quality of protein structures predicted by ESMFold is sufficient, structure prediction for 626 of 635 non-CAZyme secreted proteins of *V. dahliae* was also performed with AlphaFold v2.0 (*60*) with parameters --max_template_date=2021-05-14 --preset=casp14, with nine predictions failing due to high computational requirements. Average structure predicted local distance difference test (pLDDT) values were compared between AlphaFold and ESMFold (fig. S20). Then, AMAPEC v1.0 was used to predict the antimicrobial activity of proteins secreted by *V. dahliae*, *C. cinerea*, and *R. irregularis* while excluding CAZymes and transmembrane proteins. ESMFold (*71*)-predicted structures, which pLDDT confidence scores are in tables S5 to S7, were used as an input. To analyze the conservation of predicted antimicrobials and nonantimicrobials, an orthology prediction was computed on the three fungal secretomes with OrthoFinder v2.5.5 (*81*). The conservation of antimicrobials and nonantimicrobials was studied independently, after subsetting the OrthoFinder-generated “orthogroups” tables to only contain proteins from each group.

### Comparative genomics in a dataset of 150 fungal genomes

A set of 150 fungal genomes was assembled, based on a previously published dataset of 120 fungal genomes for which fungal lifestyles had been manually curated (*82*). This dataset was supplemented with 23 genomes of plant-pathogenic fungi and 7 genomes of Glomeromycetes (see details in table S8). An orthology prediction was performed on total sets of annotated proteins with OrthoFinder v2.5.5 (*81*) to generate a phylogenomic tree with the implemented method “STAG” (*83*). This genome-scale phylogeny is displayed on Fig. 2D and fig. S4. In all 150 genomes, sets of proteins carrying SignalP v6.0 (*52*)-annotated signal peptides were considered to form secretomes. A second orthology prediction with OrthoFinder v2.5.5 was performed on these 150 secretomes. In each secreted protein family (i.e., “orthogroup”) defined by this orthology prediction, the most central and representative protein was identified with phylorep v0.1 (*84*) using OrthoFinder-generated gene trees [relying on method FastTree (*85*)] as inputs. CAZymes and transmembrane proteins among these family representatives were annotated using dbCAN v4.0 and TMBed v.1.0.0, respectively (*79*, *80*). The structures of other representative proteins were predicted with ESMFold v1.0.3 (*71*) and then submitted to antimicrobial activity prediction with AMAPEC v1.0. Protein family conservation analyses were performed considering the presence/absence of families in each genome (i.e., considering the occurrence of orthologs but not the numbers of paralogs). Specifically, each family was assigned a conservation level according to the clade (phylum, class, order, family, or genus) its occurrence is restricted to, in the 150-genome dataset. Proportions of protein families which representative members are predicted antimicrobials/nonantimicrobials were analyzed, excluding CAZyme- and transmembrane protein-encoding families. Then, sequence diversity within the 600 most conserved secreted protein families (excluding CAZymes) was estimated through k-mer-based Shannon index calculation with MerCat2 v1.0 (*69*) using a k-mer size of three amino acids.

### Analysis of predicted antimicrobials in PHI-base

Sequences of proteins that have been studied for their contribution to host-pathogen interactions were downloaded from the PHI-base database (*37*) (accessed in January 2024). To identify proteins previously studied for their contribution to fungal virulence in the *V. dahliae* JR2 secretome, SignalP v6.0 (*52*)-predicted secreted proteins in the *V. dahliae* genome were blasted against the downloaded PHI-base sequences using blastp v2.5.0 with parameters -evalue 0.0001 -max_target_seqs 1 and additional filtering to only keep hits with more than 95% sequence identity to query proteins (table S10). To investigate more generally if previously characterized fungal effectors have antimicrobial activities, the PHI-base dataset was subsetted to only retain proteins from fungi that have secretion signal peptides according to SignalP v6.0 (*52*) and that were classified as “effectors (plant avirulence determinants)” in the database. After structure prediction with ESMFold v1.0.3 (*71*), the antimicrobial activity of these proteins was predicted with AMAPEC v1.0. To estimate the conservation of these effectors across the fungal kingdom, the protein family of the most similar protein in the 150-genome dataset was considered, and the number of genomes in which it occurs was used as a proxy for effector conservation (Fig. 3A). To identify previously characterized fungal effectors with confidently predicted antimicrobial activity and broad conservation across the fungal kingdom (Fig. 3B), the following filtering criteria were used: highly confident effector structure prediction with an average pLDDT of >70, predicted antimicrobial activity, presence of an effector homolog with more than 80% sequence identity in the 150-genome dataset, and representation of the associated family in more than 100 of 150 genomes.

### Effector protein production and purification

For heterologous production of the AGLIP1, AVR-Pita, Ecp6, Vd424Y, and VdCP1 effectors, protein sequences were retrieved from PHI-base (*37*), and then codon-optimized nucleotide sequences encoding for mature proteins were subcloned into pET-15b (AVR-Pita, Vd424Y, and AGLIP1), pET-28a(+) (VdCP1), or pETSUMO (Ecp6) expression vectors. All the constructs with an N-terminal His_6_ tag (Gene Universal, Newark, DE, USA) were transformed into *E. coli* BL21 or Shuffle cells by heat shock (45 s at 42°C, 5 min on ice). The transformed BL21 cells (AGLIP1, AVR-Pita, Vd424Y, and VdCP1) were grown at 37°C with constant shaking at 180 rpm in 2× yeast-tryptone (YT) medium [tryptone (16 g/liter), yeast extract (10 g/liter), and NaCl (5 g/liter)] containing ampicillin (100 μg/ml). Protein production was induced with 1 mM isopropyl-β-d-thiogalactopyranoside (IPTG) when cultures reached an optical density (OD_600_) of 2.0. Induction was performed for 2 hours at 37°C for AVR-Pita and Vd424Y, 4 hours at 37°C for AGLIP1, and 2 hours at 42°C for VdCP1, all with constant shaking at 180 rpm. For protein extraction, the bacterial cells were pelleted, then resuspended in denaturing 6 M guanidinium chloride (GdmCl), 10 mM β-mercaptoethanol, and 10 mM tris (pH 8.0), and incubated overnight at 4°C with continuous rotation. The lysate was centrifuged at 16,000*g* for 1 hour, and the resulting cleared supernatant was collected for protein purification by metal affinity chromatography using a nickel His60 Ni Superflow Resin (Takara, San Jose, CA, USA) column in the ÄKTA go protein purification system (Cytiva, Marlborough, MA, USA). After column equilibration with 6 M GdmCl and 10 mM tris at pH 8.0, denatured protein was loaded onto the column, and weakly bound protein was washed off with 6 M GdmCl, 10 mM tris, and 20 mM imidazole at pH 8.0 before the His_6_-tagged protein was eluted with 6 M GdmCl, 10 mM tris, and 200 mM imidazole at pH 8.0. Purified proteins were dialyzed (Spectra/Por 3 RC Dialysis Membrane Tubing 3500 Da molecular weight cutoff; Spectrum Laboratories, Rancho Dominguez, CA, USA) sequentially against 20 volumes of buffers with decreasing GdmCl concentrations (table S17) to promote refolding. Each dialysis step lasted a minimum of 24 hours. Last, depending on their isoelectric point, the proteins were dialyzed twice against 200 volumes of 30 mM sodium phosphate buffer (pH 7.0 for Vd424Y and pH 5.8 for AGLIP1) or 30 mM potassium phosphate buffer (pH 7.5 for AVR-Pita and pH 6.5 for VdCP1). Final concentrations were determined with a Qubit 4 Fluorometer (Invitrogen, Waltham, MA, USA). For the heterologous protein production of Ecp6 (*86*, *87*), *E. coli* Shuffle cells carrying pETSUMO-Ecp6 were grown at 37°C in lysogeny broth (LB) containing kanamycin (50 μg/ml) until an OD_600_ of 0.8. Protein expression was induced with 0.2 mM IPTG, followed by incubating at 18°C overnight. Cells were pelleted, resuspended in cell lysis buffer [50 mM tris-HCl (pH 7.5), 150 mM NaCl, 10% glycerol, lysozyme (6 mg/ml), deoxycholic acid (2 mg/ml), deoxyribonuclease I (DNase I; 60 μg/ml) (Roche, Basel, Switzerland; ref. 04536282001), and one protease inhibitor cocktail pill (Roche; ref. 11836170001)], and then incubated either at room temperature for 3 hours or at 4°C overnight with stirring. The lysate was centrifuged at 20,000*g* for 1 hour, and the cleared supernatant was collected for protein purification. His_6_-tagged SUMO-Ecp6 was purified using His60 Ni Superflow Resin, preequilibrated with 20 mM tris, 150 mM NaCl, and 5 mM imidazole (pH 8.0). Bound protein was eluted with 20 mM tris, 150 mM NaCl, and 300 mM imidazole (pH 8.0). The His_6_-SUMO affinity tag was removed by incubation overnight with ubiquitin-like-specific protease 1 (ULP-1) protease (Sigma-Aldrich, St. Louis, MO, USA; ref. SAE0067) in dialysis buffer [20 mM tris, 100 mM NaCl, and 2% glycerol (pH 8.0)]. Noncleaved fusion protein was removed by a second round of affinity purification using His60 Ni Superflow resin, and the flow-through containing cleaved Ecp6 was dialyzed overnight. The final protein concentration was determined spectrophotometrically at 280 nm.

### In vitro microbial growth inhibition assays

A diverse set of 12 bacteria previously isolated from tomato plants (*41*) were grown on LB agar plates at room temperature in the dark and then transferred into low-salt tryptic soy broth [ls-TSB; tryptone (17 g/liter), soy peptone (3 g/liter), sodium chloride (0.5 g/liter), dipotassium phosphate (2.5 g/liter), and glucose (2.5 g/liter)] and grown overnight at 28°C while shaking at 180 rpm. Overnight cultures were resuspended to the final OD_600_ = 0.025 in equal parts of fresh ls-TSB and AVR-Pita, Vd424Y, AGLIP1, VdCP1, or Ecp6 in phosphate buffer of the corresponding pH (see above; final protein concentration: 8 μM) or in the respective phosphate buffer only as a control. Total volumes of 100 μl were incubated in clear 96-well flat-bottom polystyrene tissue culture plates in a CLARIOstar plate reader (BMG Labtech, Ortenberg, Germany) at 25°C with double orbital shaking every 15 min (10 s at 300 rpm). The optical density was measured every 15 min at 600 nm. After OD_600_ normalization, areas under curves in the presence and absence of effector proteins were calculated using the trapezoidal method.

Four yeasts, i.e., *Pichia pastoris* GS115, *Cyberlindnera jadinii* (Deutsche Sammlung von Mikroorganismen, DSM 70167), *Debaryomyces vanrijiae* (DSM 70252), and *Rhodotorula bogoriensis* (DSM 70872), were grown on potato dextrose agar (PDA; Roth, Karlsruhe, Germany; ref. X931) at room temperature in the dark, then transferred into 0.05× potato dextrose broth (PDB), and grown overnight at 25°C while shaking at 180 rpm. Overnight cultures were resuspended to the final OD_600_ = 0.025 in equal parts of fresh 0.05× PDB and AVR-Pita, Vd424Y, or AGLIP1 in phosphate buffer of the corresponding pH (see above; final protein concentration: 8 μM) or in the phosphate buffer only as a control. In addition, spores from filamentous fungal strains of *Alternaria brassicicola*, *Cladosporium cucumerinum*, and *Trichoderma viride* (from our in-house culture collection) were harvested from PDA plates after culture at room temperature in the dark, then separated from the mycelium with a sterile 40-μm nylon filter (VWR, Radnor, PA, USA), and suspended in equal parts of 0.05× PDB and AVR-Pita, Vd424Y, or AGLIP1 in phosphate buffer of the corresponding pH (see above; final protein concentration: 8 μM) or in the respective phosphate buffer only as a control to a final concentration of 10^4^ spores/ml. Total volumes of 100 μl were incubated in clear 96-well flat-bottom polystyrene tissue culture plates at 25°C overnight. For both filamentous fungi and yeasts, fungal growth was imaged using a CKX41 inverted microscope (Olympus, Shinjuku City, Japan) with a DP20 camera (Olympus). Images were analyzed with ImageJ (*88*): Each image was first subjected to binarization and next to particle analysis to measure the total particle area.

### Analysis of the evolutionary histories of effectors with validated antimicrobial activities

The evolutionary histories of effectors AGLIP1, AVR-Pita, Vd424Y, and VdCP1 were analyzed by reconstructing protein family phylogenies. All sequences in the protein families (defined through orthology prediction in the 150-genome dataset) including these effectors were used to reconstruct maximum-likelihood phylogenetic trees using IQ-TREE v2.0.3 (model “LG,” default settings) (*89*) after multiple sequence alignment with MAFFT v7.310 (default parameters) (*90*). Since fungal LysM effectors are thought to represent a single effector family but exhibit large sequence diversity and therefore occur in multiple protein families defined by orthology prediction, the LysM effector family was identified through functional annotation, following a previously introduced procedure (*35*). In the 150-fungal genome dataset, and additionally in the genome of the fungus *C. fulvum* which secretes the well-characterized Ecp6 LysM effector (*38*), functional domains were annotated using InterProScan v5.65-97.0 (*91*). Secreted proteins containing LysM domains (IPR036779, IPR018392, and IPR045030), but not any other functional domains (e.g., chitinase domains), were considered as LysM effectors. All annotated LysM effectors were used to reconstruct a protein phylogenetic tree with IQ-TREE (model LG) after sequence alignment with MAFFT, as performed for the four other effector families. In all five effector families, antimicrobial activities were predicted using AMAPEC v1.0 after structure prediction with ESMFold v1.0.3 (*71*). Protein family phylogenetic trees were visualized and annotated with antimicrobial activity prediction results using interactive tree of life [iTOL (*92*)].

The recent evolution of the Vd424Y effector family was further studied focusing on the subfamily (clade identified on the total protein family tree; fig. S5) containing Vd424Y. As previously performed (*33*), chloroplastic transit peptides and NLSs were annotated using ChloroP v1.1 (*93*)and cNLS Mapper (*94*) (used online with default parameters in May 2025: https://nls-mapper.iab.keio.ac.jp/), respectively. To analyze sequence variations in the Vd424Y subfamily, subfamily members were aligned using MAFFT, and a figure was generated with iTOL (*92*), referring to a previously published domain annotation of Vd424Y to highlight the domain organization of the proteins (*33*).

### Measurements of *V. dahliae* effector gene expression in soils

Soil extracts were prepared from 10 previously sampled soils with distinct properties and microbial communities (*95*). To do so, 20 g of soil (stored at 4°C) was mixed with 100 ml of sterile water (1:5, w/v) followed by incubation at room temperature for 2 days. Soil particles were removed by centrifugation at 4000*g* for 30 min, and the resulting supernatant was used as soil extract.

Conidiospores of *V. dahliae* JR2 were harvested from mycelium cultured on PDA plates for 7 days. The spores were washed once with sterile water and collected by centrifugation at 10,000*g* for 2 min. Spore concentration was determined by counting with a hemocytometer, and 1 × 10^6^ spores were inoculated into 10 ml of PDB in 50-ml flasks. Cultures were incubated at 22°C with shaking at 130 rpm for 2 days. Mycelia were then collected, rinsed with sterile water, and transferred into 10 ml of prepared soil extracts in 50-ml flasks. After 2 days of incubation, the mycelia were collected using Miracloth, rinsed with sterile water, and blotted dry with tissue paper. The samples were transferred to 2-ml tubes containing two 2.3-mm iron beads, flash-frozen in liquid nitrogen, and ground using a tissue lyser. Total RNA was extracted using TRIzol reagent (Thermo Fisher Scientific, Waltham, MA, USA). For each sample, 500 ng of RNA was reverse transcribed using the HiScript III RT SuperMix for quantitative polymerase chain reaction (qPCR; +gDNA Wiper) (Vazyme Biotech Co. Ltd., Nanjing, China; ref. R323-01). The resulting cDNA was diluted 10-fold with nuclease-free water. Real-time qPCR was performed using SsoAdvanced Universal SYBR Green Supermix (Bio-Rad, Hercules, CA, USA). Primers 5′-TGTTACCAAAGCAGCACACAAGG-3′ and 5′-CCTTATGCCTCGTTCCCTTCCAC-3′ were used to amplify the Ave1-encoding gene [positive control; (*7*)], primers 5′-GCAAGCGAGGACTGACAAGATC-3′ and 5′-CGACGGAATGGACGGCGTG-3′ were used to amplify the Tom1-enconding gene [negative control; (*96*)], newly designed primers 5′-TCGGGCGGTTTCTACTACTC-3′ and 5′-TGTTGTTCTTCCAGCTGACG-3′ were used to amplify the Vd424Y-encoding gene (VDAG_JR2_Chr5g00880), and the primers 5′-CGAGTCCACTGGTGTCTTCA-3′ and 5′-CCTCAACGATGGTGAACTT-3′ were used to amplify the glyceraldehyde-3-phosphate dehydrogenase (GAPDH) gene (house-keeping gene). The PCR cycling conditions were as follows: initial denaturation at 95°C for 3 min, followed by 40 cycles of denaturation at 95°C for 10 s and annealing/extension at 60°C for 30 s with fluorescence signal collection. After amplification, a melt curve analysis was performed from 65° to 95°C, increasing by 0.5°C every 5 s, to verify the specificity of the PCR products. Gene expression levels of effector genes were normalized to the *V. dahliae* GAPDH using the ΔCt method.

### *Vd424Y* gene deletion in *V. dahliae*

Gene deletion was performed following previously published protocols for *V. dahliae* protoplast transformation (*97*) and CRISPR-Cas9-based fungal genome editing (*98*). After culturing on PDA plates at room temperature for 7 to 15 days in the dark, 3 × 10^7^ spores of *V. dahliae* JR2 were harvested from the mycelium surface and inoculated into 100 ml of liquid Complete Medium composed of 0.6% yeast extract (Duchefa, Haarlem, the Netherlands; ref. Y1333), 0.6% casein hydrolysate (Roth; ref. AE41), and 1% sucrose (VWR; ref. 0335) in Milli-Q water. After 20 hours in culture at 28°C with agitation, mycelium was harvested on a Falcon 40-μm nylon filter (ref. 352340) and washed with a sterile 0.7 M NaCl solution. Then, the harvested mycelium was incubated in 10 ml of sterile-filtered driselase solution (0.2% of enzyme in 0.7 M NaCl; Sigma-Aldrich, St. Louis, MO, USA; ref. D9515) for 2.5 hours at 33°C with agitation. The resulting solution was passed through a 40-μm nylon filter and then centrifuged at 3000*g* for 5 min. After supernatant removal, the protoplast pellet was resuspended in 1 ml of sterile STC [20% sucrose, 10 mM tris-HCl (pH 8.0), and 50 mM CaCl_2_ in Milli-Q water] and centrifuged at 3000*g* for 5 min. This last step was repeated twice to thoroughly wash the protoplasts. Protoplasts were counted under a microscope, and their concentration was adapted to 5 × 10^6^ protoplasts/ml in STC. After adding 1% of dimethyl sulfoxide, protoplast solutions were kept at −80°C.

To delete the gene of interest, two single guide RNAs (sgRNAs) were designed using CRISPick (https://portals.broadinstitute.org/gppx/crispick/public) to target the upstream and downstream regions of the Vd424Y-encoding gene (VDAG_JR2_Chr5g00880): CATACGTCCTGTTCAGCCGG (upstream) and GCCATCCGACAGCATTCAG (downstream). A blastn was performed (with parameter --word_size 11) to check that these sgRNA protospacer sequences only occur near the targeted gene in the *V. dahliae* JR2 genome. Oligonucleotides with sequences corresponding to the designed sgRNA protospacer in between sequences 5′-AAGCTAATACGACTCACTATA-3′ and 5′-GTTTTAGAGCTAGAAATAGCAAG-3′ were ordered. To synthesize sgRNA from these oligonucleotides, 1 μl of oligonucleotide (100 μM) was mixed with 1 μl of oligonucleotide 5′-AAAAGCACCGACTCGGTGCCACTTTTTCAAGTTGATAACGGACTAGCCTTATTTTAACTTGCTATTTCTAGCTCTAAAAC-3′ (100 μM) and 8 μl of nuclease-free water. This mixture was incubated in a thermocycler for annealing with the following program: 95°C for 5 min, 95° to 85°C at 2°C/s, and 85° to 25°C at −0.1°C/s. Then, 2.5 μl of deoxynucleoside triphosphates (10 mM each), 2 μl of NEBuffer r2.1 (10×, New England Biolabs, Ipswich, MA, USA; ref. B6002S), 0.5 μl of T4 DNA polymerase (New England Biolabs; ref. M0203S), and 5 μl nuclease-free water were added to the mixture, followed by an incubation at 12°C for 20 min. Products of this reaction were purified with a Monarch PCR and DNA Cleanup Kit (New England Biolabs; ref. T1030L). Concentration in DNA was then measured with a NanoDrop device. To transcribe the DNA molecules into sgRNA, 2 μg of DNA was mixed with 6 μl of RNA ribonucleoside triphosphates (25 mM each), 1.5 μl of T7 buffer (10×), 1.5 μl of HiScribe T7 polymerase (New England Biolabs; ref. E2040S), 1 μl of dithiothreitol (0.1 M), and nuclease-free water up to 20 μl total volume. After incubation overnight at 37°C, 14 μl of nuclease-free water, 4 μl of RQ1 DNase buffer (10×, Promega, Walldorf, Germany; ref. M198A), and 2 μl of RQ1 DNAse (Promega; ref. M610A) were added in the reaction tube. An incubation of 30 min at 37°C followed to digest the remaining DNA. sgRNA molecules were then purified using an RNA Clean and Concentrator (Zymo Research, Irvine, CA, USA; ref. R1017 & R1018) kit. Purified sgRNA were stored at −80°C until transformation.

Double-stranded donor DNA corresponding to the 50-bp sequence upstream of the expected Cas9-mediated cut in the fungal genome, followed by the 50-bp sequence downstream of the second expected cut, was synthesized. Through homologous recombination, this donor DNA promoted genome repair excluding our target gene upon double-stranded cuts by Cas9.

The commercial enzyme EnGen Spy Cas9 HF1 (New England Biolabs; ref. M0667M) was used for protoplast transformation. First, 4 μM of this Cas9 enzyme was mixed to 2× of NEBuffer r3.1 (New England Biolabs; ref. B6003S) and sgRNA (0.3 μg/μl). The mixture was incubated at 25°C for 30 min to bind sgRNA and enzyme. Both prebound Cas9-sgRNA complexes were then mixed with 200 μl of fungal protoplasts (5 × 10^6^ protoplasts/ml), 20 pmol of double-stranded donor DNA, and 6 μg of telomeric vector pTEL-Hyg (*98*) containing a hygromycin resistance gene. After 30 min of incubation on ice, 1.5 ml of PEG-STC [60% polyethylene glycol 4000, 20% of sucrose, 10 mM tris-HCl (pH 8.0), and 50 mM CaCl_2_ in Milli-Q water] was added to the mixture and gently mixed by tube rotation. This tube was incubated at room temperature for 15 min. Then, 5 ml of TB3 medium (3% yeast extract, 3% casein hydrolysate, and 20% sucrose in water) was added to the mixture, stimulating protoplast regeneration over an 18-hour incubation in the dark at room temperature.

Regeneration medium was centrifuged at 3000*g* for 5 min. The supernatant was removed and pelleted fungi were resuspended in sterile water to be plated on PDA medium containing hygromycin (50 μg/ml). Plates were incubated at room temperature for 5 days. Then, visible colonies were screened by PCR using a pair of primers targeting ~500 bp upstream and downstream of the gene of interest (forward: 5′-ACATATCGCGACGAGTTCCC-3′, reverse: 5′-CTCTTCTTCTCGAGCGACCC-3′). One colony for which the amplicon size was the one expected upon successful gene deletion was identified. Sanger sequencing of the amplicon confirmed the successful gene deletion. After cultivation of this mutant on PDA + hygromycin plate, spores were harvested and inoculated on hygromycin-free PDA medium. A colony that successfully grew in the absence of hygromycin and that was confirmed to have lost the pTEL-Hyg vector was used as Vd424Y deletion line (∆424Y).

Before to be used in plant recolonization experiments, the newly generated mutant line was tested to verify it is not impaired in growth. *V. dahliae* JR2 wild-type and the ∆424Y mutant were cultured on PDA plates for 10 days. Conidiospores were harvested and washed twice with sterile Milli-Q water. The final concentration was adjusted to 1 × 10^6^ spores/ml in 1 ml of PDB medium. This spore solution was incubated horizontally at 25°C with shaking for 48 hours. Following incubation, fungal cells were collected by centrifugation at 13,000 rpm for 5 min, and 900 μl of the supernatant was carefully removed to avoid disturbing the pellet. As an internal control, 1 ng of synthetic spike-in plasmid (*99*) was added to each sample, and genomic DNA was extracted from the resulting pellets using the DNeasy PowerSoil Pro Kit (QIAGEN, Hilden, Germany; ref. 47014), following the manufacturer’s protocol. *V. dahliae* biomass was quantified by real-time PCR using species-specific primers VdITS1-F (5′-AAAGTTTTAATGGTTCGCTAAGA-3′) and STVe1-R (5′’-CTTGGTCATTTAGAGGAAGTAA-3′) targeting the internal transcribed spacer (ITS) region. The spike-in plasmid was amplified in the same solutions using primers qRT-Spike-F (5′-TTTCTTTTCCAAGGTTTGTGC-3′) and qRT-Spike-R (5′-AACATTTACCCTGCTTGTAGCTCT-3′). A fungal growth index was calculated from both amplifications’ Ct values: index = 2^-(CtITS/CtSpike)^. The growth index values of *V. dahliae* wild-type and ∆424Y mutant were then compared to check that the ∆424Y mutant line is not impaired in growth (fig. S21).

### Tomato plant inoculation assays in a gnotobiotic system

The protocol applied here follows an adaptation of the FlowPot gnotobiotic system (*100*) to tomato plant inoculations with *V. dahliae* strains (*41*). FlowPot substrate, a 1:1 mixture of peat and vermiculite (Balster Einheitserde, Frödenberg, Germany; LIMERA Gartenbauservice, Geldern-Walbeck, Germany), was sterilized by two consecutive autoclaving rounds. Following substrate sterilization, FlowPot units were created by filling truncated 50-ml Luer lock syringes (Terumo Europe, Leuven, Belgium) with sterilized substrate and autoclaving for a third time on a liquid cycle. In addition, a recolonized condition was created, by mixing sterilized substrate with nonsterile substrate in a 9:1 ratio, followed by an incubation at room temperature overnight. To remove toxic compounds, which may accumulate during autoclaving, the substrate was flushed using 30 ml of sterile Milli-Q water using a vacuum system. Further, substrate was enriched using 30 ml of half-strength Murashige and Skoog medium (Duchefa; ref. M0222). Following FlowPot preparation, tomato seeds (*Solanum lycopersium* L.; cultivar “MoneyMaker”) were surface sterilized as described previously (*101*), stratified at 8°C for 24 hours, and sown into each FlowPot unit. Subsequently, up to five FlowPot units were placed into Microbox containers with four air filters (SacO2; Deinze, Belgium) and kept in a greenhouse (17 hours of light at 23°C followed by 7 hours of darkness at 22°C). After 14 days of growth, tomato plants were inoculated using *V. dahliae* wild-type and ∆424Y strains as well as ∆Ave1 (*7*) as a control. To this end, Microboxes were opened under a sterile hood, and plants were carefully uprooted from the substrate. Roots were rinsed using sterile Milli-Q water and subsequently placed into a *V. dahliae* spore suspension, containing 10^6^ spores/ml. After 8 min of incubation, plants were placed back into the FlowPots, and Microboxes were placed back into the greenhouse chamber. Symptom development was assessed at 14 days postinoculation by measuring tomato shoot fresh weight.

### Microbiota profiling and analysis

Tomato plants inoculated with *V. dahliae* wild-type and ∆424Y strains grown in recolonized soil were harvested. Stem samples were collected by cutting the region between the soil surface and the lowest leaf and were manually ground with a mortar and a pestle. Total DNA was extracted following a phenol chloroform-based extraction method (*102*). The bacterial *16S* ribosomal RNA gene was PCR-amplified using the universal primer pair 27F (5′-AGAGTTTGATCCTGGCTCAG-3′) and 1492R (5′-GGWTACCTTGTTACGACT-3′). To suppress coamplification of host-derived mitochondrial and chloroplast DNA, peptide nucleic acid (PNA) blocking clamps mPNA (GGCAAGTGTTCTTCGGA) and pPNA (GGCTCAACCCTGGACAG) (PNABio, Newbury Park, USA) were included in the PCR reactions, each at a final concentration of 0.25 μM. PCR products were purified using the Monarch PCR and DNA cleanup kit (New England Biolabs, Ipswich, USA). Sequencing libraries were prepared using the Native Barcoding Kit 96 v14 (SQK-NBD114.96) according to the manufacturer’s protocol (Oxford Nanopore Technologies, Oxford, UK). Barcoded libraries were pooled and sequenced on a MinION sequencing device (Oxford Nanopore Technologies). Sequenced reads were processed using porechop v0.2.4 to remove Nanopore adapters and then using cutadapt v5.2 to exclude reads shorter than 1000 nucleotides in length (parameter -m 1000). The filtered sets of *16S* reads were assigned to bacterial taxa using the method Emu v3.4.6 and its default database (*103*). To profile differences in bacterial microbiota compositions between samples, Bray-Curtis distances were calculated using the function spatial.distance.pdist(metric=“braycurtis”) of the Python library SciPy v1.13.1 (*104*), and a principal coordinates analysis (PCoA) was computed on these distances. A permutational multivariate analysis of variance (PERMANOVA) test was computed with the function adonis2(perm=999) of the R package vegan v2.6.4 to assess whether *V. dahliae* wild-type– and ∆424Y–inoculated plants have significantly different microbiota compositions (formula *BrayCurtis~InoculatedFungus*). To identify bacteria that may be suppressed by the Vd424Y effector, numbers of reads assigned to each bacterial taxon were summed up at the genus level. Then, a differential abundance analysis was computed using DESeq v1.40.2 with default parameters (*105*). Since a single genus was found significantly differentially abundant between *V. dahliae* wild-type– and ∆424Y–inoculated plants (adjusted *P* < 0.05) due to the small number of sequenced samples and the inherent variability of the microbiota that limits the statistical power of the analysis, the 10 bacterial genera showing the lowest log-transformed fold change values were considered as candidate targets of the Vd424Y effector.

### *Agrobacterium*-mediated transient effector gene expressions in *N. benthamiana*

Orthologs of Vd424Y were identified in two phylogenetically distant saprotrophic fungi: *C. cinerea* AmutBmut pab1-1(basidiomycete) and *P. restrictum* MPI-SP2-AT-0405 (ascomycete). While the genome assembly and annotation of *C. cinerea* were available through JGI mycocosm (ID: Copci_AmutBmut1) (*74**,* *75*), no genome assembly was available for *P. restrictum* MPI-SP2-AT-0405. Therefore, previously sequenced raw reads (*106*) were downloaded from the European Nucleotide Archive (ENA project PRJEB50298, sample SAMEA12383883, run ERR8084596), assembled with Flye v2.9.2 (default parameters) and annotated with BRAKER v3.0.8 (mode --fungus) (*107*, *108*). Orthologs of Vd424Y were identified in these two genomes using blastp v2.5.0+ on their set of annotated proteins. The ortholog of Vd424Y in *C. cinerea* was named Cc424Y (gene locus: scaffold_317:14532-15602), and the one in *P. restrictum* was named Pr424Y (gene locus: contig_7:133414-134175). The sequences of these proteins are provided in table S18.

Total RNA was isolated from *V. dahliae*, *C. cinerea*, and *P. restrictum* cultured on PDA medium, and the coding sequences of the 424Y homologs were amplified using the primer pairs 35*S*:Vd424Y-F (5′-TTACGAACGATAGCATCTAGAATGGTCTCGTTCACTTCTCTCCT-3′) and 35*S*:Vd424Y-R (5′-GTAGTCCATCCCGGGGGTACCAGACACAGTCATGGTGGCGC-3′), 35*S*:Cc424Y-F (5′-TTACGAACGATAGCATCTAGAATGAAGTTCTCTTCTCTCTTCGTTG-3′) and 35*S*:Cc424Y-R (5′-GTAGTCCATCCCGGGGGTACCGGAGACGGTGATGGTAGCG-3′), and 35*S*:Pr424Y-F (5′-TTACGAACGATAGCATCTAGAATGGTTTCTTTCACTTCTTTGATCGC-3′) and 35*S*:Pr424Y-R (5′-GTAGTCCATCCCGGGGGTACCGCTGACTGTGATAGTCGAGGA-3′), respectively, and cloned into the pCNF3 vector (*109*) by homologous recombination. *Pr424Y* carrying the *Vd424Y* NLS (Pr424Y-NLS) was synthesized by BioCat GmbH (Heidelberg, Germany) and inserted into the same vector. The 424Y homologs were C-terminally fused to yellow fluorescent protein (YFP) using the primer pair YFP-F (5′-GGGGTACCATGGTGAGCAAGGGCGAGG-3′) and YFP-R (5′-TCCCCCGGGCTTGTACAGCTCGTCCATGC-3′) in the pCNF3-424Y recombinant vectors, so their subcellular localization can be assessed. Then, the recombinant vectors 35Spro:Vd424Y-Flag/YFP, 35Spro:Cc424Y-Flag/YFP, 35Spro:Pr424Y-Flag/YFP, and 35Spro:Pr424YNLS-Flag/YFP were transformed into *Agrobacterium tumefaciens* strain GV3101. The p19 silencing suppressor from tomato bushy stunt virus was used to enhance transient expression. *A. tumefaciens* cultures carrying the expression constructs (kanamycin + rifampicin) or p19 (ampicillin, rifampicin + gentamicin) were grown overnight at 28°C in LB medium. Bacterial cells were collected by centrifugation at 4000*g* for 10 min and resuspended in infiltration buffer [10 mM MgCl_2_, 10 mM MES (pH 5.6), and 150 μM acetosyringone]. The suspensions were adjusted to OD_600_ = 1 and incubated at room temperature for 2 to 3 hours before infiltration. Next, *A. tumefaciens* suspensions carrying the expression construct and p19 were mixed in 1:1 ratio and infiltrated into the leaves of 3- to 4-week-old *N. benthamiana* plants using a needle-less syringe. After infiltration, the plants were kept under high humidity in the dark overnight, after which the plants were grown under normal conditions (22 to 24°C, 16 hours light/8 hours dark). After 7 days, the occurrence of cell death was recorded.

The expression of the 424Y proteins by *N. benthamiana* was validated by Western blot (fig. S22). Leaf tissue was ground into a fine powder, mixed with extraction buffer [150 mM NaCl, 1.0 NP-40, and 50 mM tris (pH 8.0)], and incubated on ice for 20 to 30 min. The mixture was then centrifuged at 12,000 rpm for 15 min at 4°C. After collecting the supernatant, anti-Flag magnetic beads (MedChemExpress, Monmouth Junction, USA) were added, and the mixture was incubated overnight at 4°C. Next, the beads were washed three times with phosphate-buffered saline buffer (0.137 M NaCl, 2.7 mM KCl, 1.8 mM KH_2_PO_4_, and 10 mM Na_2_HPO_4_), resuspended in 60 μl of water with 20 μl of loading buffer, incubated at 98°C for 10 min, and subjected to conventional SDS–polyacrylamide gel electrophoresis. The resulting protein blot was hybridized with α-Flag antibody (Sigma-Aldrich, St. Louis, USA) and developed with SuperSignal West Pico PLUS Chemiluminescence-Substrate (ref. 34577, Thermo Fisher Scientific, Waltham, MA, USA).

To assess subcellular localization, *A. tumefaciens* suspensions encoding YFP-tagged 424Y homologs and Sts2-mCherry (*110*) were coinfiltrated into the leaves of 3- to 4-week-old *N. benthamiana* plants. After 2 to 3 days, fluorescence signals were observed using a confocal laser scanning microscope (SP8, Leica, Wetzlar, Germany). YFP was excited at 514 nm and detected at 527 nm, while mCherry was excited at 587 nm and detected at 610 nm.

### *Pr424Y* gene deletion in *P. restrictum* and soil colonization assays

A *P. restrictum* gene deletion mutant lacking the *Pr424Y* gene (locus: contig_7:133414-134175) was generated essentially following the protocol used to generate the *V. dahliae* ∆*424Y* mutant while using GAGATCGGCAGAAATCCACA (upstream) and GCCCAGGGACAAAGCCCCAG (downstream) as sgRNA protospacer sequences. Following protoplast transformation and regeneration, gene deletion mutants were screened for with PCR using primers 5′-AAGCGATGGCTGGTTTCAGA-3′ and 5′-TGTTCTTCGCACCATACCCC-3′.

Cologne agricultural soil (CAS) (*111*) was autoclaved twice to obtain sterile soil. Briefly, after initial autoclaving, the soil was thoroughly mixed using a sterile spoon in a laminar flow cabinet and autoclaved once more. For sterile soil treatment, 1 g of autoclaved soil was weighed into a 15-ml sterile tube. For the recolonized soil treatment, 10% (w/w) nonautoclaved CAS was mixed with 90% autoclaved CAS, and 1 g of the mixture was transferred into a 15-ml sterile tube. All soil samples were incubated at room temperature in the dark for 2 days before inoculation.

Wild-type and *424Y* deletion strains of *P. restrictum* were grown on PDA medium at room temperature for 7 days. Conidiospores were harvested by adding 5 ml of sterile Milli-Q water, gently scraping the surface, and filtering through a cell strainer to remove mycelial fragments. After centrifugation at 10,000*g* for 2 min, the conidiospores were washed twice with sterile water. A spore suspension of 10^3^ conidiospores ml^−1^ was prepared in a 50-ml tube containing 20 ml of PDB medium and incubated overnight at room temperature while shaking at 60 rpm. Next, cultures were pelleted by centrifugation at 4000 rpm for 15 min, washed twice with sterile Milli-Q water, and resuspended in 20 ml of sterile Milli-Q water. A total of 100 μl of fungal suspension was added to 1 g of the soil samples and incubated at room temperature in the dark. Soil samples were collected at 0 and 14 days after inoculation, thoroughly homogenized using a sterile spatula, and 250 mg of soil was transferred into PowerBead tubes for DNA extraction using the DNeasy PowerSoil Pro Kit (QIAGEN, Hilden, Germany). One nanogram of spike-in plasmid was added to the CD1 lysis buffer of each sample for calibration (*99*). Last, DNA was eluted in 50 μl of sterile Milli-Q water.

Real-time qPCR was performed using SsoAdvanced Universal SYBR Green Supermix (Bio-Rad, Hercules, CA, USA) with primers 5′-TTTCTTTTCCAAGGTTTGTGC-3′ and 5′-AACATTTACCCTGCTTGTAGCTCT-3′ for the spike-in and 5′-GGAGGCATCAGCAAGTACCA-3′ and 5′-GGCTGGTTCCACCCATACTC-3′ for *P. restrictum* (designed for specifically targetting the *RPB1* gene of this species). The qPCR cycling conditions consisted of 95°C for 3 min, followed by 40 two-step cycles of denaturation at 95°C for 10 s and annealing/extension at 60°C for 30 s, with fluorescence signal acquisition at the end of each cycle. Melt curve analysis was performed from 65° to 95°C, with temperature increments of 0.5°C every 5 s, to verify the specificity of the amplification products. Fungal relative biomass was calculated by 2^Ct(*P. restrictum*) − Ct(Spike-in)^.

### Statistics

Fisher’s exact tests were computed using the function stats.fisher_exact in SciPy v1.13.0 (*104*). Mann-Whitney *U* tests were performed using the function stats.mannwhitneyu of SciPy v1.13.0. Cochrane-Armitage tests for trend were calculated with the function stats.contingency_tables.Table.test_ordinal_association of statsmodels v0.14.0 (*112*). In the case of multiple testing, *P* values from the tests mentioned above were adjusted using Benjamini-Hochberg correction with the function stats.multitest.multipletests(method=“fdr_bh”) of statsmodels v0.14.0. Measurements from in vitro microbial growth restriction assays were analyzed in R v4.4.1, first by using the shapiro.test function and Q-Q plots to assess normality of the datasets (Shapiro-Wilk test *P* > 0.05), and then since all data were normally distributed, pairwise one-sided Student’s *t* tests comparing microbial growth in the presence and absence of protein were performed using the function t.test(alternative=“less”). To analyze the tomato shoot fresh weight measurements, a square root transformation {in R: lm[sqrt(ShootFreshWeight)~Condition]} was applied to reach normal distribution of the data (Shapiro-Wilk test *P* > 0.05), and then statistical comparison between treatments was performed using an analysis of variance (ANOVA) test and a Tukey’s post hoc test (functions aov and TukeyHSD of R v4.2.0). Letters reflecting the significant differences between treatments were obtained with function multcompLetters4 from R package multcompView v0.1-1 (*113*). Significant differences between conditions of the *P. restrictum* soil colonization assays were assessed with a Kruskal-Wallis test, since data were not normally distributed (Shapiro-Wilk test *P <* 0.05), using the function kruskal.test in R v4.2.0. Post hoc testing was performed with a Dunn test using the function DunnTest of the R package DescTools v0.99.50.

## Supplementary Materials

### The PDF file includes:

Figs. S1 to S22 Legends for tables S1 to S18 References

## References

[1] D. E. Cook, C. H. Mesarich, B. P. H. J. Thomma, Understanding plant immunity as a surveillance system to detect invasion. Annu. Rev. Phytopathol. 53, 541–563 (2015). 26047564 10.1146/annurev-phyto-080614-120114

[2] G. Z. Han, Origin and evolution of the plant immune system. New Phytol. 222, 70–83 (2019). 30575972 10.1111/nph.15596

[3] J. D. G. Jones, B. J. Staskawicz, J. L. Dangl, The plant immune system: From discovery to deployment. Cell 187, 2095–2116 (2024). 38670067 10.1016/j.cell.2024.03.045

[4] V. Müller, R. J. de Boer, S. Bonhoeffer, E. Szathmáry, An evolutionary perspective on the systems of adaptive immunity. Biol. Rev. 93, 505–528 (2018). 28745003 10.1111/brv.12355

[5] I. Stergiopoulos, P. J. G. M. De Wit, Fungal effector proteins. Annu. Rev. Phytopathol. 47, 233–263 (2009). 19400631 10.1146/annurev.phyto.112408.132637

[6] G. Doehlemann, B. Ökmen, W. Zhu, A. Sharon, Plant pathogenic fungi. Microbiol. Spectr. 5, 10.1128/microbiolspec.FUNK-0023-2016 (2017). 10.1128/microbiolspec.funk-0023-2016 PMC11687436 28155813

[7] N. C. Snelders, H. Rovenich, G. C. Petti, M. Rocafort, G. C. M. van den Berg, J. A. Vorholt, J. R. Mesters, M. F. Seidl, R. Nijland, B. P. H. J. Thomma, Microbiome manipulation by a soil-borne fungal plant pathogen using effector proteins. Nat. Plants 6, 1365–1374 (2020). 33139860 10.1038/s41477-020-00799-5

[8] N. C. Snelders, G. C. Petti, G. C. M. van den Berg, M. F. Seidl, B. P. H. J. Thomma, An ancient antimicrobial protein co-opted by a fungal plant pathogen for in planta mycobiome manipulation. Proc. Natl. Acad. Sci. U.S.A. 118, e2110968118 (2021). 34853168 10.1073/pnas.2110968118 PMC8670511

[9] N. C. Snelders, J. C. Boshoven, Y. Song, N. Schmitz, G. L. Fiorin, H. Rovenich, G. C. M. van den Berg, D. E. Torres, G. C. Petti, Z. Prockl, L. Faino, M. F. Seidl, B. P. H. J. Thomma, A highly polymorphic effector protein promotes fungal virulence through suppression of plant-associated Actinobacteria. New Phytol. 237, 944–958 (2023). 36300791 10.1111/nph.18576

[10] E. A. Chavarro-Carrero, N. C. Snelders, D. E. Torres, A. Kraege, A. López-Moral, G. C. Petti, W. Punt, J. Wieneke, R. García-Velasco, C. J. López-Herrera, M. F. Seidl, B. P. H. J. Thomma, The soil-borne white root rot pathogen *Rosellinia necatrix* expresses antimicrobial proteins during host colonization. PLOS Pathog. 20, e1011866 (2024). 38236788 10.1371/journal.ppat.1011866 PMC10796067

[11] B. Ökmen, P. Katzy, L. Huang, R. Wemhöner, G. Doehlemann, A conserved extracellular Ribo1 with broad-spectrum cytotoxic activity enables smut fungi to compete with host-associated bacteria. New Phytol. 240, 1976–1989 (2023). 37680042 10.1111/nph.19244

[12] D. Gómez-Pérez, M. Schmid, V. Chaudhry, Y. Hu, A. Velic, B. Maček, J. Ruhe, A. Kemen, E. Kemen, Proteins released into the plant apoplast by the obligate parasitic protist *Albugo* selectively repress phyllosphere-associated bacteria. New Phytol. 239, 2320–2334 (2023). 37222268 10.1111/nph.18995

[13] H. X. Chang, Z. A. Noel, M. I. Chilvers, A β-lactamase gene of *Fusarium oxysporum* alters the rhizosphere microbiota of soybean. Plant J. 106, 1588–1604 (2021). 33788336 10.1111/tpj.15257

[14] A. Kraege, W. Punt, A. Doddi, J. Zhu, N. Schmitz, N. C. Snelders, B. P. H. J. Thomma, Undermining the cry for help: The phytopathogenic fungus *Verticillium dahliae* secretes an antimicrobial effector protein to undermine host recruitment of antagonistic *Pseudomonas* bacteria. New Phytol. 249, 406–417 (2026). 41163408 10.1111/nph.70686 PMC12676067

[15] M. Möller, E. H. Stukenbrock, Evolution and genome architecture in fungal plant pathogens. Nat. Rev. Microbiol. 15, 756–771 (2017). 28781365 10.1038/nrmicro.2017.76

[16] L. J. Ma, H. C. Van Der Does, K. A. Borkovich, J. J. Coleman, M. J. Daboussi, A. Di Pietro, M. Dufresne, M. Freitag, M. Grabherr, B. Henrissat, P. M. Houterman, S. Kang, W. B. Shim, C. Woloshuk, X. Xie, J. R. Xu, J. Antoniw, S. E. Baker, B. H. Bluhm, A. Breakspear, D. W. Brown, R. A. E. Butchko, S. Chapman, R. Coulson, P. M. Coutinho, E. G. J. Danchin, A. Diener, L. R. Gale, D. M. Gardiner, S. Goff, K. E. Hammond-Kosack, K. Hilburn, A. Hua-Van, W. Jonkers, K. Kazan, C. D. Kodira, M. Koehrsen, L. Kumar, Y. H. Lee, L. Li, J. M. Manners, D. Miranda-Saavedra, M. Mukherjee, G. Park, J. Park, S. Y. Park, R. H. Proctor, A. Regev, M. C. Ruiz-Roldan, D. Sain, S. Sakthikumar, S. Sykes, D. C. Schwartz, B. G. Turgeon, I. Wapinski, O. Yoder, S. Young, Q. Zeng, S. Zhou, J. Galagan, C. A. Cuomo, H. C. Kistler, M. Rep, Comparative genomics reveals mobile pathogenicity chromosomes in *Fusarium*. Nature 464, 367–373 (2010). 20237561 10.1038/nature08850 PMC3048781

[17] R. de Jonge, M. D. Bolton, A. Kombrink, G. C. M. Van Den Berg, K. A. Yadeta, B. P. H. J. Thomma, Extensive chromosomal reshuffling drives evolution of virulence in an asexual pathogen. Genome Res. 23, 1271–1282 (2013). 23685541 10.1101/gr.152660.112 PMC3730101

[18] Y. Sato, R. Bex, G. C. M. van den Berg, P. Santhanam, M. Höfte, M. F. Seidl, B. P. H. J. Thomma, Starship giant transposons dominate plastic genomic regions in a fungal plant pathogen and drive virulence evolution. Nat. Commun. 16, 1–17 (2025). 40707455 10.1038/s41467-025-61986-6 PMC12289983

[19] M. Torrent, D. Andreu, V. M. Nogués, E. Boix, Connecting peptide physicochemical and antimicrobial properties by a rational prediction model. PLOS ONE 6, e16968 (2011). 21347392 10.1371/journal.pone.0016968 PMC3036733

[20] G. Wang, The antimicrobial peptide database is 20 years old: Recent developments and future directions. Protein Sci. 32, e4778 (2023). 37695921 10.1002/pro.4778 PMC10535814

[21] F. Wan, F. Wong, J. J. Collins, C. de la Fuente-Nunez, Machine learning for antimicrobial peptide identification and design. Nat. Rev. Bioeng. 2, 392–407 (2024). 39850516 10.1038/s44222-024-00152-x PMC11756916

[22] R. Eichfeld, L. K. Mahdi, C. De Quattro, L. Armbruster, A. B. Endeshaw, S. Miyauchi, M. J. Hellmann, S. Cord-Landwehr, D. Peterson, V. Singan, K. Lail, E. Savage, V. Ng, I. V. Grigoriev, G. Langen, B. M. Moerschbacher, A. Zuccaro, Transcriptomics reveal a mechanism of niche defense: Two beneficial root endophytes deploy an antimicrobial GH18-CBM5 chitinase to protect their hosts. New Phytol. 244, 980–996 (2024). 39224928 10.1111/nph.20080

[23] F. Chen, L. Ou, H. Wu, L. Huang, Y.-P. Chen, “Expression and characterization of the antifungal protein PtAFP from *Pyrenophora tritci-repentis* by synonymous codon bias in *Escherichia coli*,” in *Proc. SPIE 13208*, *Third Int. Conf. Biomed. Intell. Syst.* (*IC-BIS 2024*) (2024), vol. 13208, pp. 13–19.

[24] K. de Guillen, L. Mammri, J. Gracy, A. Padilla, P. Barthe, F. Hoh, M. Lahfa, J. Rouffet, Y. Petit-Houdenot, T. Kroj, M.-H. Lebrun, *Zymoseptoria tritici* effectors structurally related to killer proteins UmV-KP4 and UmV-KP6 inhibit fungal growth, and define extended protein families in fungi. Mol. Plant Pathol. 26, e70141 (2025). 40864528 10.1111/mpp.70141 PMC12382754

[25] Z. Sorger, P. Sengupta, K. Beier-Heuchert, J. Bautor, J. E. Parker, E. Kemen, G. Doehlemann, GH25 lysozyme mediates tripartite interkingdom interactions and microbial competition on the plant leaf surface. Proc. Natl. Acad. Sci. U.S.A. 122, e2510124122 (2025). 41201826 10.1073/pnas.2510124122 PMC12626018

[26] L. Florez, V. M. Flores-Núñez, C. S. Francisco, E. Holtgrewe Stukenbrock, The fungal effector AvrStb6 regulates the wheat pathobiome. *Zenodo* (2025). 10.5281/zenodo.15852925.

[27] F. Mesny, AMAPEC v1.0. *Zenodo* (2026). 10.5281/zenodo.18220951.

[28] E. F. Fradin, B. P. H. J. Thomma, Physiology and molecular aspects of *Verticillium* wilt diseases caused by *V. dahliae* and *V. albo-atrum*. Mol. Plant Pathol. 7, 71–86 (2006). 20507429 10.1111/j.1364-3703.2006.00323.x

[29] N. C. Snelders, H. Rovenich, B. P. H. J. Thomma, Microbiota manipulation through the secretion of effector proteins is fundamental to the wealth of lifestyles in the fungal kingdom. FEMS Microbiol. Rev. 46, fuac022 (2022). 35604874 10.1093/femsre/fuac022 PMC9438471

[30] G. J. Kettles, C. Bayon, C. A. Sparks, G. Canning, K. Kanyuka, J. J. Rudd, Characterization of an antimicrobial and phytotoxic ribonuclease secreted by the fungal wheat pathogen *Zymoseptoria tritici*. New Phytol. 217, 320–331 (2018). 28895153 10.1111/nph.14786 PMC5724701

[31] Y. Zhang, Y. Gao, Y. Liang, Y. Dong, X. Yang, J. Yuan, D. Qiu, The *Verticillium dahliae* SnodProt1-like protein VdCP1 contributes to virulence and triggers the plant immune system. Front. Plant Sci. 8, 1880 (2017). 29163605 10.3389/fpls.2017.01880 PMC5671667

[32] L. Liu, Z. Wang, J. Li, Y. Wang, J. Yuan, J. Zhan, P. Wang, Y. Lin, F. Li, X. Ge, *Verticillium dahliae* secreted protein Vd424Y is required for full virulence, targets the nucleus of plant cells, and induces cell death. Mol. Plant Pathol. 22, 1109–1120 (2021). 34233072 10.1111/mpp.13100 PMC8358993

[33] D. Wang, J. Y. Chen, J. Song, J. J. Li, S. J. Klosterman, R. Li, Z. Q. Kong, K. V. Subbarao, X. F. Dai, D. D. Zhang, Cytotoxic function of xylanase VdXyn4 in the plant vascular wilt pathogen *Verticillium dahliae*. Plant Physiol. 187, 409–429 (2021). 34618145 10.1093/plphys/kiab274 PMC8418393

[34] A. Kombrink, H. Rovenich, X. Shi-Kunne, E. Rojas-Padilla, G. C. M. van den Berg, E. Domazakis, R. de Jonge, D. J. Valkenburg, A. Sánchez-Vallet, M. F. Seidl, B. P. H. J. Thomma, *Verticillium dahliae* LysM effectors differentially contribute to virulence on plant hosts. Mol. Plant Pathol. 18, 596–608 (2017). 27911046 10.1111/mpp.12520 PMC6638240

[35] R. de Jonge, B. P. H. J. Thomma, Fungal LysM effectors: Extinguishers of host immunity? Trends Microbiol. 17, 151–157 (2009). 19299132 10.1016/j.tim.2009.01.002

[36] A. Kombrink, B. P. H. J. Thomma, LysM effectors: Secreted proteins supporting fungal life. PLOS Pathog. 9, e1003769 (2013). 24348247 10.1371/journal.ppat.1003769 PMC3861536

[37] M. Urban, A. Cuzick, J. Seager, V. Wood, K. Rutherford, S. Y. Venkatesh, J. Sahu, S. Vijaylakshmi Iyer, L. Khamari, N. De Silva, M. C. Martinez, H. Pedro, A. D. Yates, K. E. Hammond-Kosack, PHI-base in 2022: A multi-species phenotype database for pathogen–host Interactions. Nucleic Acids Res. 50, D837–D847 (2022). 34788826 10.1093/nar/gkab1037 PMC8728202

[38] R. de Jonge, H. P. Van Esse, A. Kombrink, T. Shinya, Y. Desaki, R. Bours, S. Van Der Krol, N. Shibuya, M. H. A. J. Joosten, B. P. H. J. Thomma, Conserved fungal LysM effector Ecp6 prevents chitin-triggered immunity in plants. Science 329, 953–955 (2010). 20724636 10.1126/science.1190859

[39] S. Li, X. Peng, Y. Wang, K. Hua, F. Xing, Y. Zheng, W. Liu, W. Sun, S. Wei, The effector AGLIP1 in *Rhizoctonia solani* AG1 IA triggers cell death in plants and promotes disease development through inhibiting PAMP-triggered immunity in *Arabidopsis thaliana*. Front. Microbiol. 10, 2228 (2019). 31611861 10.3389/fmicb.2019.02228 PMC6775501

[40] G. Xiao, N. Laksanavilat, S. Cesari, K. Lambou, M. Baudin, A. Jalilian, M. J. Telebanco-Yanoria, V. Chalvon, I. Meusnier, E. Fournier, D. Tharreau, B. Zhou, J. Wu, T. Kroj, The unconventional resistance protein PTR recognizes the *Magnaporthe oryzae* effector AVR-Pita in an allele-specific manner. Nat. Plants 10, 994–1004 (2024). 38834685 10.1038/s41477-024-01694-z

[41] W. Punt, J. Park, H. Roevenich, A. Kraege, N. Schmitz, J. Wieneke, N. C. Snelders, G. L. Fiorin, A. López-Moral, E. A. Chavarro-Carrero, G. C. Petti, K. Wippel, B. P. H. J. Thomma, A gnotobiotic system reveals multifunctional effector roles in plant-fungal pathogen dynamics. bioRxiv 645772 [Preprint] (2025). 10.1101/2025.03.27.645772.

[42] S. Kumar, G. Stecher, M. Suleski, S. B. Hedges, TimeTree: A resource for timelines, timetrees, and divergence times. Mol. Biol. Evol. 34, 1812–1819 (2017). 28387841 10.1093/molbev/msx116

[43] T. Y. James, F. Kauff, C. L. Schoch, P. B. Matheny, V. Hofstetter, C. J. Cox, G. Celio, C. Gueidan, E. Fraker, J. Miadlikowska, H. T. Lumbsch, A. Rauhut, V. Reeb, A. E. Arnold, A. Amtoft, J. E. Stajich, K. Hosaka, G. H. Sung, D. Johnson, B. O’Rourke, M. Crockett, M. Binder, J. M. Curtis, J. C. Slot, Z. Wang, A. W. Wilson, A. Schüßler, J. E. Longcore, K. O’Donnell, S. Mozley-Standridge, D. Porter, P. M. Letcher, M. J. Powell, J. W. Taylor, M. M. White, G. W. Griffith, D. R. Davies, R. A. Humber, J. B. Morton, J. Sugiyama, A. Y. Rossman, J. D. Rogers, D. H. Pfister, D. Hewitt, K. Hansen, S. Hambleton, R. A. Shoemaker, J. Kohlmeyer, B. Volkmann-Kohlmeyer, R. A. Spotts, M. Serdani, P. W. Crous, K. W. Hughes, K. Matsuura, E. Langer, G. Langer, W. A. Untereiner, R. Lücking, B. Büdel, D. M. Geiser, A. Aptroot, P. Diederich, I. Schmitt, M. Schultz, R. Yahr, D. S. Hibbett, F. Lutzoni, D. J. McLaughlin, J. W. Spatafora, R. Vilgalys, Reconstructing the early evolution of Fungi using a six-gene phylogeny. Nature 443, 818–822 (2006). 17051209 10.1038/nature05110

[44] X. Yuan, S. Xiao, T. N. Taylor, Lichen-like symbiosis 600 million years ago. Science 308, 1017–1020 (2005). 15890881 10.1126/science.1111347

[45] M. A. Guerreiro, E. H. Stukenbrock, Fungal plant pathogens. Curr. Biol. 35, R480–R484 (2025). 40494300 10.1016/j.cub.2025.02.046

[46] C. Y. Huang, K. Araujo, J. N. Sánchez, G. Kund, J. Trumble, C. Roper, K. E. Godfrey, H. Jin, A stable antimicrobial peptide with dual functions of treating and preventing citrus Huanglongbing. Proc. Natl. Acad. Sci. U.S.A. 118, e2019628118 (2021). 33526689 10.1073/pnas.2019628118 PMC8017978

[47] D. Wu, L. Fu, W. Wen, N. Dong, The dual antimicrobial and immunomodulatory roles of host defense peptides and their applications in animal production. J. Anim. Sci. Biotechnol. 13, 141 (2022). 36474280 10.1186/s40104-022-00796-y PMC9724304

[48] R. Eichfeld, A. B. Endeshaw, M. J. Hellmann, B. M. Moerschbacher, A. Zuccaro, Domain gain or loss in fungal chitinases drives ecological specialization toward antagonism or immune suppression. bioRxiv 659886 [Preprint] (2025). 10.1101/2025.06.16.659886. PMC13039781 41912547

[49] P. van Dam, L. Fokkens, S. M. Schmidt, J. H. J. Linmans, H. Corby Kistler, L. J. Ma, M. Rep, Effector profiles distinguish formae speciales of *Fusarium oxysporum*. Environ. Microbiol. 18, 4087–4102 (2016). 27387256 10.1111/1462-2920.13445

[50] F. Mesny, M. Bauer, J. Zhu, B. P. H. J. Thomma, Meddling with the microbiota: Fungal tricks to infect plant hosts. Curr. Opin. Plant Biol. 82, 102622 (2024). 39241281 10.1016/j.pbi.2024.102622

[51] A. C. Sexton, B. J. Howlett, Parallels in fungal pathogenesis on plant and animal hosts. Eukaryot. Cell 5, 1941–1949 (2006). 17041185 10.1128/EC.00277-06 PMC1694825

[52] F. Teufel, J. J. Almagro Armenteros, A. R. Johansen, M. H. Gíslason, S. I. Pihl, K. D. Tsirigos, O. Winther, S. Brunak, G. von Heijne, H. Nielsen, SignalP 6.0 predicts all five types of signal peptides using protein language models. Nat. Biotechnol. 40, 1023–1025 (2022). 34980915 10.1038/s41587-021-01156-3 PMC9287161

[53] P. K. Meher, T. K. Sahu, V. Saini, A. R. Rao, Predicting antimicrobial peptides with improved accuracy by incorporating the compositional, physico-chemical and structural features into Chou’s general PseAAC. Sci. Rep. 7, 1–12 (2017). 28205576 10.1038/srep42362 PMC5304217

[54] D. Veltri, U. Kamath, A. Shehu, Deep learning improves antimicrobial peptide recognition. Bioinformatics 34, 2740–2747 (2018). 29590297 10.1093/bioinformatics/bty179 PMC6084614

[55] T.-T. Lin, L.-Y. Yang, I.-H. Lu, W.-C. Cheng, Z.-R. Hsu, S.-H. Chen, C.-Y. Lin, AI4AMP: An antimicrobial peptide predictor using physicochemical property-based encoding method and deep learning. mSystems 6, e0029921 (2021). 34783578 10.1128/mSystems.00299-21 PMC8594441

[56] H. Lee, S. Lee, I. Lee, H. Nam, AMP-BERT: Prediction of antimicrobial peptide function based on a BERT model. Protein Sci. 32, e4529 (2023). 36461699 10.1002/pro.4529 PMC9793967

[57] J. Yan, P. Bhadra, A. Li, P. Sethiya, L. Qin, H. K. Tai, K. H. Wong, S. W. I. Siu, Deep-AmPEP30: Improve short antimicrobial peptides prediction with deep learning. Mol. Ther.Nucleic Acids 20, 882–894 (2020). 32464552 10.1016/j.omtn.2020.05.006 PMC7256447

[58] A. Bateman, M. J. Martin, S. Orchard, M. Magrane, R. Agivetova, S. Ahmad, E. Alpi, E. H. Bowler-Barnett, R. Britto, B. Bursteinas, H. Bye-A-Jee, R. Coetzee, A. Cukura, A. Da Silva, P. Denny, T. Dogan, T. G. Ebenezer, J. Fan, L. G. Castro, P. Garmiri, G. Georghiou, L. Gonzales, E. Hatton-Ellis, A. Hussein, A. Ignatchenko, G. Insana, R. Ishtiaq, P. Jokinen, V. Joshi, D. Jyothi, A. Lock, R. Lopez, A. Luciani, J. Luo, Y. Lussi, A. MacDougall, F. Madeira, M. Mahmoudy, M. Menchi, A. Mishra, K. Moulang, A. Nightingale, C. S. Oliveira, S. Pundir, G. Qi, S. Raj, D. Rice, M. R. Lopez, R. Saidi, J. Sampson, T. Sawford, E. Speretta, E. Turner, N. Tyagi, P. Vasudev, V. Volynkin, K. Warner, X. Watkins, R. Zaru, H. Zellner, A. Bridge, S. Poux, N. Redaschi, L. Aimo, G. Argoud-Puy, A. Auchincloss, K. Axelsen, P. Bansal, D. Baratin, M. C. Blatter, J. Bolleman, E. Boutet, L. Breuza, C. Casals-Casas, E. de Castro, K. C. Echioukh, E. Coudert, B. Cuche, M. Doche, D. Dornevil, A. Estreicher, M. L. Famiglietti, M. Feuermann, E. Gasteiger, S. Gehant, V. Gerritsen, A. Gos, N. Gruaz-Gumowski, U. Hinz, C. Hulo, N. Hyka-Nouspikel, F. Jungo, G. Keller, A. Kerhornou, V. Lara, P. Le Mercier, D. Lieberherr, T. Lombardot, X. Martin, P. Masson, A. Morgat, T. B. Neto, S. Paesano, I. Pedruzzi, S. Pilbout, L. Pourcel, M. Pozzato, M. Pruess, C. Rivoire, C. Sigrist, K. Sonesson, A. Stutz, S. Sundaram, M. Tognolli, L. Verbregue, C. H. Wu, C. N. Arighi, L. Arminski, C. Chen, Y. Chen, J. S. Garavelli, H. Huang, K. Laiho, P. McGarvey, D. A. Natale, K. Ross, C. R. Vinayaka, Q. Wang, Y. Wang, L. S. Yeh, J. Zhang, UniProt: The universal protein knowledgebase in 2021. Nucleic Acids Res. 49, D480–D489 (2021). 33237286 10.1093/nar/gkaa1100 PMC7778908

[59] D. Osorio, P. Rondón-Villarreal, R. Torres, Peptides: A package for data mining of antimicrobial peptides. R J. 7, 4–14 (2015).

[60] J. Jumper, R. Evans, A. Pritzel, T. Green, M. Figurnov, O. Ronneberger, K. Tunyasuvunakool, R. Bates, A. Žídek, A. Potapenko, A. Bridgland, C. Meyer, S. A. A. Kohl, A. J. Ballard, A. Cowie, B. Romera-Paredes, S. Nikolov, R. Jain, J. Adler, T. Back, S. Petersen, D. Reiman, E. Clancy, M. Zielinski, M. Steinegger, M. Pacholska, T. Berghammer, S. Bodenstein, D. Silver, O. Vinyals, A. W. Senior, K. Kavukcuoglu, P. Kohli, D. Hassabis, Highly accurate protein structure prediction with AlphaFold. Nature 596, 583–589 (2021). 34265844 10.1038/s41586-021-03819-2 PMC8371605

[61] P. J. A. Cock, T. Antao, J. T. Chang, B. A. Chapman, C. J. Cox, A. Dalke, I. Friedberg, T. Hamelryck, F. Kauff, B. Wilczynski, M. J. L. de Hoon, Biopython: Freely available Python tools for computational molecular biology and bioinformatics. Bioinformatics 25, 1422–1423 (2009). 19304878 10.1093/bioinformatics/btp163 PMC2682512

[62] N. Mih, E. Brunk, K. Chen, E. Catoiu, A. Sastry, E. Kavvas, J. M. Monk, Z. Zhang, B. O. Palsson, ssbio: A Python framework for structural systems biology. Bioinformatics 34, 2155–2157 (2018). 29444205 10.1093/bioinformatics/bty077 PMC6658713

[63] H. Chen, F. Gu, Z. Huang, Improved Chou-Fasman method for protein secondary structure prediction. BMC Bioinformatics 7, 1–11 (2006). 17217506 10.1186/1471-2105-7-S4-S14 PMC1780123

[64] R. Nagarajan, A. Archana, A. M. Thangakani, S. Jemimah, D. Velmurugan, M. M. Gromiha, PDBparam: Online resource for computing structural parameters of proteins. Bioinform. Biol. Insights 10, 73–80 (2016). 27330281 10.4137/BBI.S38423 PMC4909059

[65] W. Kabsch, C. Sander, Dictionary of protein secondary structure: Pattern recognition of hydrogen-bonded and geometrical features. Biopolymers 22, 2577–2637 (1983). 6667333 10.1002/bip.360221211

[66] W. G. Touw, C. Baakman, J. Black, T. A. H. Te Beek, E. Krieger, R. P. Joosten, G. Vriend, A series of PDB-related databanks for everyday needs. Nucleic Acids Res. 43, D364–D368 (2015). 25352545 10.1093/nar/gku1028 PMC4383885

[67] V. Le Guilloux, P. Schmidtke, P. Tuffery, Fpocket: An open source platform for ligand pocket detection. BMC Bioinformatics 10, 1–11 (2009). 19486540 10.1186/1471-2105-10-168 PMC2700099

[68] Y. Liang, S. Yang, L. Zheng, H. Wang, J. Zhou, S. Huang, L. Yang, Y. Zuo, Research progress of reduced amino acid alphabets in protein analysis and prediction. Comput. Struct. Biotechnol. J. 20, 3503–3510 (2022). 35860409 10.1016/j.csbj.2022.07.001 PMC9284397

[69] J. L. Figueroa, A. Redinbo, A. Panyala, S. Colby, M. L. Friesen, L. Tiemann, R. A. White, MerCat2: A versatile k-mer counter and diversity estimator for database-independent property analysis obtained from omics data. Bioinforma. Adv. 4, vbae061 (2024). 10.1093/bioadv/vbae061 PMC11090762 38745763

[70] F. Pedregosa, G. Varoquaux, A. Gramfort, M. Vincent, B. Thirion, O. Grisel, M. Blondel, P. Prettenhofer, R. Weiss, V. Dubourg, J. Vanderplas, A. Passos, D. Cournapeau, M. Brucher, M. Perrot, É. Duchesnay, Scikit-learn: Machine learning in Python. J. Mach. Learn. Res. 12, 2825–2830 (2011).

[71] Z. Lin, H. Akin, R. Rao, B. Hie, Z. Zhu, W. Lu, N. Smetanin, R. Verkuil, O. Kabeli, Y. Shmueli, A. Dos Santos Costa, M. Fazel-Zarandi, T. Sercu, S. Candido, A. Rives, Evolutionary-scale prediction of atomic-level protein structure with a language model. Science 379, 1123–1130 (2023). 36927031 10.1126/science.ade2574

[72] R. de Jonge, H. P. Van Esse, K. Maruthachalam, M. D. Bolton, P. Santhanam, M. K. Saber, Z. Zhang, T. Usami, B. Lievens, K. V. Subbarao, B. P. H. J. Thomma, Tomato immune receptor Ve1 recognizes effector of multiple fungal pathogens uncovered by genome and RNA sequencing. Proc. Natl. Acad. Sci. U.S.A. 109, 5110–5115 (2012). 22416119 10.1073/pnas.1119623109 PMC3323992

[73] P. J. Kersey, J. E. Allen, I. Armean, S. Boddu, B. J. Bolt, D. Carvalho-Silva, M. Christensen, P. Davis, L. J. Falin, C. Grabmueller, J. Humphrey, A. Kerhornou, J. Khobova, N. K. Aranganathan, N. Langridge, E. Lowy, M. D. McDowall, U. Maheswari, M. Nuhn, C. K. Ong, B. Overduin, M. Paulini, H. Pedro, E. Perry, G. Spudich, E. Tapanari, B. Walts, G. Williams, M. Tello–Ruiz, J. Stein, S. Wei, D. Ware, D. M. Bolser, K. L. Howe, E. Kulesha, D. Lawson, G. Maslen, D. M. Staines, Ensembl Genomes 2016: More genomes, more complexity. Nucleic Acids Res. 44, D574–D580 (2016). 26578574 10.1093/nar/gkv1209 PMC4702859

[74] H. Muraguchi, K. Umezawa, M. Niikura, M. Yoshida, T. Kozaki, K. Ishii, K. Sakai, M. Shimizu, K. Nakahori, Y. Sakamoto, C. Choi, C. Y. Ngan, E. Lindquist, A. Lipzen, A. Tritt, S. Haridas, K. Barry, I. V. Grigoriev, P. J. Pukkila, Strand-specific RNA-seq analyses of fruiting body development in *Coprinopsis cinerea*. PLOS ONE 10, e0141586 (2015). 26510163 10.1371/journal.pone.0141586 PMC4624876

[75] I. V. Grigoriev, R. Nikitin, S. Haridas, A. Kuo, R. Ohm, R. Otillar, R. Riley, A. Salamov, X. Zhao, F. Korzeniewski, T. Smirnova, H. Nordberg, I. Dubchak, I. Shabalov, MycoCosm portal: Gearing up for 1000 fungal genomes. Nucleic Acids Res. 42, D699–D704 (2014). 24297253 10.1093/nar/gkt1183 PMC3965089

[76] G. Yildirir, J. Sperschneider, M. Malar C, E. C. H. Chen, W. Iwasaki, C. Cornell, N. Corradi, Long reads and Hi-C sequencing illuminate the two-compartment genome of the model arbuscular mycorrhizal symbiont *Rhizophagus irregularis*. New Phytol. 233, 1097–1107 (2022). 34747029 10.1111/nph.17842

[77] C. P. Cantalapiedra, A. Hernández-Plaza, I. Letunic, P. Bork, J. Huerta-Cepas, eggNOG-mapper v2: Functional annotation, orthology assignments, and domain prediction at the metagenomic scale. Mol. Biol. Evol. 38, 5825–5829 (2021). 34597405 10.1093/molbev/msab293 PMC8662613

[78] J. Huerta-Cepas, D. Szklarczyk, D. Heller, A. Hernández-Plaza, S. K. Forslund, H. Cook, D. R. Mende, I. Letunic, T. Rattei, L. J. Jensen, C. Von Mering, P. Bork, eggNOG 5.0: A hierarchical, functionally and phylogenetically annotated orthology resource based on 5090 organisms and 2502 viruses. Nucleic Acids Res. 47, D309–D314 (2019). 30418610 10.1093/nar/gky1085 PMC6324079

[79] J. Zheng, Q. Ge, Y. Yan, X. Zhang, L. Huang, Y. Yin, dbCAN3: Automated carbohydrate-active enzyme and substrate annotation. Nucleic Acids Res. 51, W115–W121 (2023). 37125649 10.1093/nar/gkad328 PMC10320055

[80] M. Bernhofer, B. Rost, TMbed: Transmembrane proteins predicted through language model embeddings. BMC Bioinformatics 23, 1–19 (2022). 35941534 10.1186/s12859-022-04873-x PMC9358067

[81] D. M. Emms, S. Kelly, OrthoFinder: Phylogenetic orthology inference for comparative genomics. Genome Biol. 20, 1–14 (2019). 31727128 10.1186/s13059-019-1832-y PMC6857279

[82] F. Mesny, S. Miyauchi, T. Thiergart, B. Pickel, L. Atanasova, M. Karlsson, B. Hüttel, K. W. Barry, S. Haridas, C. Chen, D. Bauer, W. Andreopoulos, J. Pangilinan, K. LaButti, R. Riley, A. Lipzen, A. Clum, E. Drula, B. Henrissat, A. Kohler, I. V. Grigoriev, F. M. Martin, S. Hacquard, Genetic determinants of endophytism in the *Arabidopsis* root mycobiome. Nat. Commun. 12, 1–15 (2021). 34893598 10.1038/s41467-021-27479-y PMC8664821

[83] D. M. Emms, S. Kelly, STAG: Species tree inference from all genes. bioRxiv 267914 [Preprint] (2018). 10.1101/267914.

[84] F. Mesny, phylorep v0.1. *Zenodo* (2023). 10.5281/ZENODO.10142123.

[85] M. N. Price, P. S. Dehal, A. P. Arkin, FastTree 2—Approximately maximum-likelihood trees for large alignments. PLOS ONE 5, e9490 (2010). 20224823 10.1371/journal.pone.0009490 PMC2835736

[86] G. L. Fiorin, A. Sanchéz-Vallet, D. P. d. T. Thomazella, P. F. V. do Prado, L. C. do Nascimento, A. V. d. O. Figueira, B. P. H. J. Thomma, G. A. G. Pereira, P. J. P. L. Teixeira, Suppression of plant immunity by fungal chitinase-like effectors. Curr. Biol. 28, 3023–3030.e5 (2018). 30220500 10.1016/j.cub.2018.07.055

[87] H. Tian, C. I. MacKenzie, L. Rodriguez-Moreno, G. C. M. van den Berg, H. Chen, J. J. Rudd, J. R. Mesters, B. P. H. J. Thomma, Three LysM effectors of *Zymoseptoria tritici* collectively disarm chitin-triggered plant immunity. Mol. Plant Pathol. 22, 683–693 (2021). 33797163 10.1111/mpp.13055 PMC8126183

[88] C. A. Schneider, W. S. Rasband, K. W. Eliceiri, NIH Image to ImageJ: 25 years of image analysis. Nat. Methods 9, 671–675 (2012). 22930834 10.1038/nmeth.2089 PMC5554542

[89] B. Q. Minh, H. A. Schmidt, O. Chernomor, D. Schrempf, M. D. Woodhams, A. Von Haeseler, R. Lanfear, E. Teeling, IQ-TREE 2: New models and efficient methods for phylogenetic inference in the genomic era. Mol. Biol. Evol. 37, 1530–1534 (2020). 32011700 10.1093/molbev/msaa015 PMC7182206

[90] K. Katoh, D. M. Standley, MAFFT multiple sequence alignment software version 7: Improvements in performance and usability. Mol. Biol. Evol. 30, 772–780 (2013). 23329690 10.1093/molbev/mst010 PMC3603318

[91] P. Jones, D. Binns, H. Y. Chang, M. Fraser, W. Li, C. McAnulla, H. McWilliam, J. Maslen, A. Mitchell, G. Nuka, S. Pesseat, A. F. Quinn, A. Sangrador-Vegas, M. Scheremetjew, S. Y. Yong, R. Lopez, S. Hunter, InterProScan 5: Genome-scale protein function classification. Bioinformatics 30, 1236–1240 (2014). 24451626 10.1093/bioinformatics/btu031 PMC3998142

[92] I. Letunic, P. Bork, Interactive Tree of Life (iTOL) v6: Recent updates to the phylogenetic tree display and annotation tool. Nucleic Acids Res. 52, W78–W82 (2024). 38613393 10.1093/nar/gkae268 PMC11223838

[93] O. Emanuelsson, H. Nielsen, G. Von Heijne, ChloroP, a neural network-based method for predicting chloroplast transit peptides and their cleavage sites. Protein Sci. 8, 978–984 (1999). 10338008 10.1110/ps.8.5.978 PMC2144330

[94] S. Kosugi, M. Hasebe, M. Tomita, H. Yanagawa, Systematic identification of cell cycle-dependent yeast nucleocytoplasmic shuttling proteins by prediction of composite motifs. Proc. Natl. Acad. Sci. U.S.A. 106, 10171–10176 (2009). 19520826 10.1073/pnas.0900604106 PMC2695404

[95] W. Punt, A. Kraege, S. Metzger, N. Schmitz, J. Zhu, S. Hacquard, M. Bonkowski, N. C. Snelders, B. P. H. J. Thomma, Differential contributions of an antimicrobial effector from *Verticillium dahliae* to virulence and tomato microbiota assembly across natural soils. Springer Nature, Microbiome (2026). 14:111. 10.1186/s40168-026-02376-y. PMC13064239 41840442

[96] J. Li, L. Faino, G. L. Fiorin, S. Bashyal, A. Schaveling, C. van den Berg, M. F. Seidl, B. PHJ Thomma, B. Thomma, A single *Verticillium dahliae* effector determines pathogenicity on tomato by targeting auxin response factors. bioRxiv 517554 [Preprint] (2022). 10.1101/2022.11.22.517554.

[97] L. Rehman, X. Su, H. Guo, X. Qi, H. Cheng, Protoplast transformation as a potential platform for exploring gene function in *Verticillium dahliae*. BMC Biotechnol. 16, 57 (2016). 27455996 10.1186/s12896-016-0287-4 PMC4960691

[98] T. Leisen, F. Bietz, J. Werner, A. Wegner, U. Schaffrath, D. Scheuring, F. Willmund, A. Mosbach, G. Scalliet, M. Hahn, CRISPR/Cas with ribonucleoprotein complexes and transiently selected telomere vectors allows highly efficient marker-free and multiple genome editing in *Botrytis cinerea*. PLOS Pathog. 16, e1008326 (2020). 32804988 10.1371/journal.ppat.1008326 PMC7451986

[99] X. Guo, X. Zhang, Y. Qin, Y. X. Liu, J. Zhang, N. Zhang, K. Wu, B. Qu, Z. He, X. Wang, X. Zhang, S. Hacquard, X. Fu, Y. Bai, Host-associated quantitative abundance profiling reveals the microbial load variation of root microbiome. Plant Commun. 1, 100003 (2020). 33404537 10.1016/j.xplc.2019.100003 PMC7747982

[100] J. M. Kremer, B. C. Paasch, D. Rhodes, C. Thireault, J. E. Froehlich, P. Schulze-Lefert, J. M. Tiedje, S. Y. He, FlowPot axenic plant growth system for microbiota research. bioRxiv 254953 [Preprint] (2018). 10.1101/254953.

[101] B. Schlesier, F. Bréton, H. P. Mock, A hydroponic culture system for growing *Arabidopsis thaliana* plantlets under sterile conditions. Plant Mol. Biol. Report. 21, 449–456 (2003).

[102] E. A. Chavarro-Carrero, J. P. Vermeulen, D. E. Torres, T. Usami, H. J. Schouten, Y. Bai, M. F. Seidl, B. P. H. J. Thomma, Comparative genomics reveals the *in planta*-secreted *Verticillium dahliae* Av2 effector protein recognized in tomato plants that carry the V2 resistance locus. Environ. Microbiol. 23, 1941–1958 (2021). 33078534 10.1111/1462-2920.15288 PMC8246953

[103] K. D. Curry, Q. Wang, M. G. Nute, A. Tyshaieva, E. Reeves, S. Soriano, Q. Wu, E. Graeber, P. Finzer, W. Mendling, T. Savidge, S. Villapol, A. Dilthey, T. J. Treangen, Emu: Species-level microbial community profiling of full-length 16S rRNA Oxford Nanopore sequencing data. Nat. Methods 19, 845–853 (2022). 35773532 10.1038/s41592-022-01520-4 PMC9939874

[104] P. Virtanen, R. Gommers, T. E. Oliphant, M. Haberland, T. Reddy, D. Cournapeau, E. Burovski, P. Peterson, W. Weckesser, J. Bright, S. J. van der Walt, M. Brett, J. Wilson, K. J. Millman, N. Mayorov, A. R. J. Nelson, E. Jones, R. Kern, E. Larson, C. J. Carey, İ. Polat, Y. Feng, E. W. Moore, J. VanderPlas, D. Laxalde, J. Perktold, R. Cimrman, I. Henriksen, E. A. Quintero, C. R. Harris, A. M. Archibald, A. H. Ribeiro, F. Pedregosa, P. van Mulbregt, SciPy 1.0 Contributors, SciPy 1.0: Fundamental algorithms for scientific computing in Python. Nat. Methods 17, 261–272 (2020). 32015543 10.1038/s41592-019-0686-2 PMC7056644

[105] M. I. Love, W. Huber, S. Anders, Moderated estimation of fold change and dispersion for RNA-seq data with DESeq2. Genome Biol. 15, 550 (2014). 25516281 10.1186/s13059-014-0550-8 PMC4302049

[106] F. Meyer, A. Fritz, Z.-L. Deng, D. Koslicki, T. R. Lesker, A. Gurevich, G. Robertson, M. Alser, D. Antipov, F. Beghini, D. Bertrand, J. J. Brito, C. T. Brown, J. Buchmann, A. Buluç, B. Chen, R. Chikhi, P. T. L. C. Clausen, A. Cristian, P. W. Dabrowski, A. E. Darling, R. Egan, E. Eskin, E. Georganas, E. Goltsman, M. A. Gray, L. H. Hansen, S. Hofmeyr, P. Huang, L. Irber, H. Jia, T. S. Jørgensen, S. D. Kieser, T. Klemetsen, A. Kola, M. Kolmogorov, A. Korobeynikov, J. Kwan, N. LaPierre, C. Lemaitre, C. Li, A. Limasset, F. Malcher-Miranda, S. Mangul, V. R. Marcelino, C. Marchet, P. Marijon, D. Meleshko, D. R. Mende, A. Milanese, N. Nagarajan, J. Nissen, S. Nurk, L. Oliker, L. Paoli, P. Peterlongo, V. C. Piro, J. S. Porter, S. Rasmussen, E. R. Rees, K. Reinert, B. Renard, E. M. Robertsen, G. L. Rosen, H.-J. Ruscheweyh, V. Sarwal, N. Segata, E. Seiler, L. Shi, F. Sun, S. Sunagawa, S. J. Sørensen, A. Thomas, C. Tong, M. Trajkovski, J. Tremblay, G. Uritskiy, R. Vicedomini, Z. Wang, Z. Wang, Z. Wang, A. Warren, N. P. Willassen, K. Yelick, R. You, G. Zeller, Z. Zhao, S. Zhu, J. Zhu, R. Garrido-Oter, P. Gastmeier, S. Hacquard, S. Häußler, A. Khaledi, F. Maechler, F. Mesny, S. Radutoiu, P. Schulze-Lefert, N. Smit, T. Strowig, A. Bremges, A. Sczyrba, A. C. McHardy, Critical Assessment of Metagenome Interpretation: The second round of challenges. Nat. Methods 19, 429–440 (2022). 35396482 10.1038/s41592-022-01431-4 PMC9007738

[107] M. Kolmogorov, J. Yuan, Y. Lin, P. A. Pevzner, Assembly of long, error-prone reads using repeat graphs. Nat. Biotechnol. 37, 540–546 (2019). 30936562 10.1038/s41587-019-0072-8

[108] L. Gabriel, T. Brůna, K. J. Hoff, M. Ebel, A. Lomsadze, M. Borodovsky, M. Stanke, BRAKER3: Fully automated genome annotation using RNA-seq and protein evidence with GeneMark-ETP, AUGUSTUS, and TSEBRA. Genome Res. 34, 769–777 (2024). 38866550 10.1101/gr.278090.123 PMC11216308

[109] G. Yang, L. Tang, Y. Gong, J. Xie, Y. Fu, D. Jiang, G. Li, D. B. Collinge, W. Chen, J. Cheng, A cerato-platanin protein SsCP1 targets plant PR1 and contributes to virulence of *Sclerotinia sclerotiorum*. New Phytol. 217, 739–755 (2018). 29076546 10.1111/nph.14842

[110] W. Zuo, J. R. L. Depotter, S. C. Stolze, H. Nakagami, G. Doehlemann, A transcriptional activator effector of *Ustilago maydis* regulates hyperplasia in maize during pathogen-induced tumor formation. Nat. Commun. 14, 6722 (2023). 37872143 10.1038/s41467-023-42522-w PMC10593772

[111] Y. Bai, D. B. Müller, G. Srinivas, R. Garrido-Oter, E. Potthoff, M. Rott, N. Dombrowski, P. C. Münch, S. Spaepen, M. Remus-Emsermann, B. Hüttel, A. C. McHardy, J. A. Vorholt, P. Schulze-Lefert, Functional overlap of the *Arabidopsis* leaf and root microbiota. Nature 528, 364–369 (2015). 26633631 10.1038/nature16192

[112] S. Seabold, J. Perktold, statsmodels: Econometric and statistical modeling with Python. SciPy 7, 92–96 (2010).

[113] S. Graves, H.-P. Piepho, L. Selzer, multcompView: Visualizations of paired comparisons. (2024). https://github.com/lselzer/multcompview.

[114] C. L. Schoch, S. Ciufo, M. Domrachev, C. L. Hotton, S. Kannan, R. Khovanskaya, D. Leipe, R. McVeigh, K. O’Neill, B. Robbertse, S. Sharma, V. Soussov, J. P. Sullivan, L. Sun, S. Turner, I. Karsch-Mizrachi, NCBI Taxonomy: A comprehensive update on curation, resources and tools. Database 2020, baaa062 (2020). 32761142 10.1093/database/baaa062 PMC7408187

[115] N. Istifadah, J. A. Saleeba, P. A. McGee, Isolates of endophytic *Chaetomium* spp. inhibit the fungal pathogen *Pyrenophora tritici-repentis* in vitro. Can. J. Bot. 84, 1148–1155 (2006).

[116] T. Sakamoto, J. M. Ortega, Taxallnomy: An extension of NCBI Taxonomy that produces a hierarchically complete taxonomic tree. BMC Bioinformatics 22, 1–23 (2021). 34325658 10.1186/s12859-021-04304-3 PMC8323199

[117] G. Wang, X. Li, Z. Wang, APD3: The antimicrobial peptide database as a tool for research and education. Nucleic Acids Res. 44, D1087–D1093 (2016). 26602694 10.1093/nar/gkv1278 PMC4702905

[118] L. V. Hedges, Distribution theory for Glass’s estimator of effect size and related estimators. J. Educ. Stat. 6, 107–128 (1981).

