# Supplementary material for: Plant-associated fungi co-opt ancient antimicrobials for host manipulation
Source: Sci Adv. 2026 Apr 29;12(18):eaec1406. doi: 10.1126/sciadv.aec1406 (PMC13127573; doi:10.1126/sciadv.aec1406)
Supplement: Supplementary file 1 — Figs. S1 to S22 Legends for tables S1 to S18 References [file sciadv.aec1406_sm.pdf]

Science Advances
AAAS

Supplementary Materials for

Plant-associated fungi co-opt ancient antimicrobials for host manipulation

Fantin Mesny *et al.*

Corresponding author: Bart P. H. J. Thomma, bthomma@uni-koeln.de

Sci. Adv. **12**, eaec1406 (2026)
DOI: 10.1126/sciadv.aec1406

The PDF file includes:

Figs. S1 to S22
Legends for tables S1 to S18
References

Other Supplementary Material for this manuscript includes the following:

Tables S1 to S18

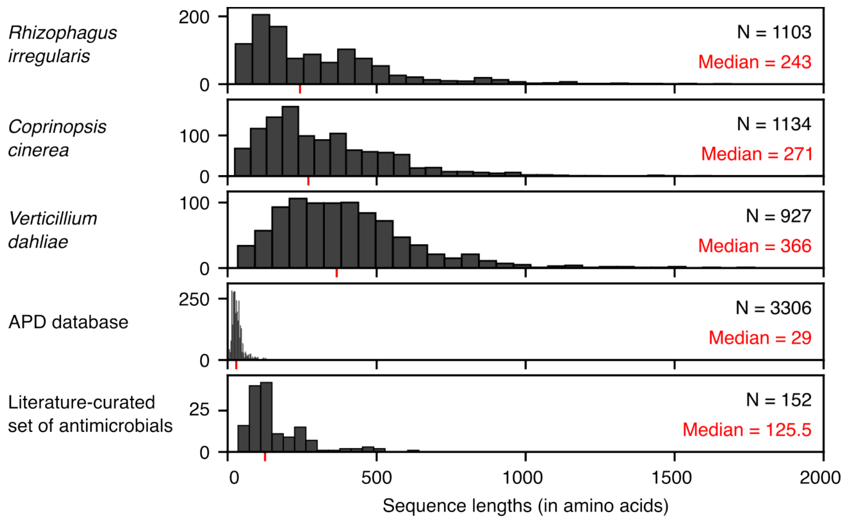

Fig. S1. Protein sequence lengths in three fungal secretomes, the AMP database APD and a literature-curated set of antimicrobials. The top three histograms show mature sequence lengths (in number of amino acids) of secreted proteins predicted with SignalP (*52*) in three fungi selected based on their distance in the tree of life and their distinct lifestyles: the Glomeromycota mycorrhizal fungus *Rhizophagus irregularis*, the Basidiomycete saprophyte *Coprinopsis cinerea*, and the Ascomycete plant pathogen *Verticillium dahliae*. The fourth histogram shows the length of AMPs in the APD database (*117*), which was previously used to train published antimicrobial peptide predictors. The bottom histogram shows sequence lengths in our newly curated set of antimicrobial proteins, used as a positive training set to develop AMAPEC.

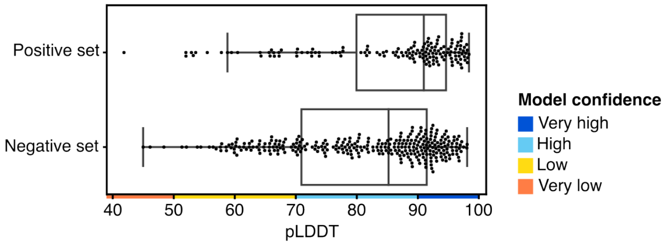

Fig. S2. Confidence of predicted structures in the training datasets. Boxplots showing the distribution of mean pLDDT confidence scores of AlphaFold-predicted protein structures in the positive and negative training sets. The color code depicting model confidence originates from the AlphaFold documentation (*60*).

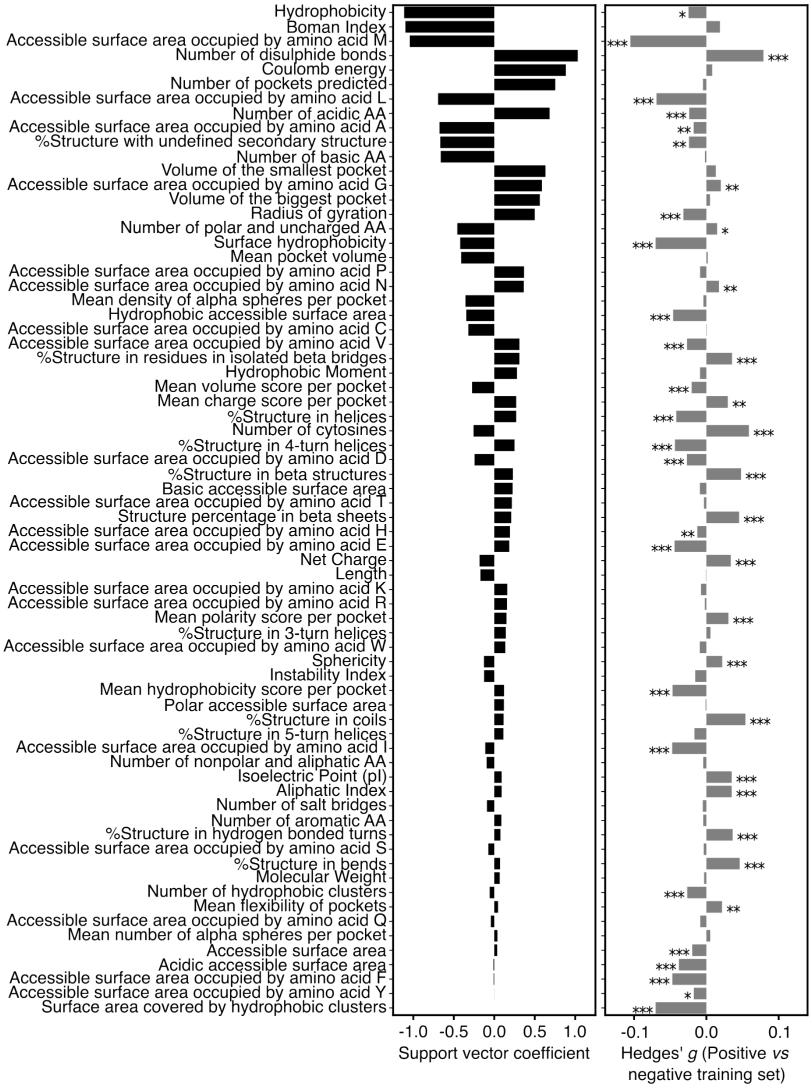

Fig. S3. Physicochemical properties of proteins and their importance for antimicrobial activity prediction. Physicochemical properties implemented in the AMAPEC training pipeline (Fig. 1, C) are listed and ranked according to their importance for our Support Vector Machines (SVM) classifier (vector weights). The left barplot (black) shows support vector coefficients, representing vector weights and orientation. The right barplot (grey) shows the results of an enrichment analysis testing for significant differences between values in the positive and in the negative training set. This analysis was conducted by Mann-Whitney U test and Benjamini-Hochberg correction (FDR values depicted with asterisks: *: ≤0.05; **≤0.01; ***≤0.001) and we additionally calculated standard effect sizes (Hedges' g, (*118*)).

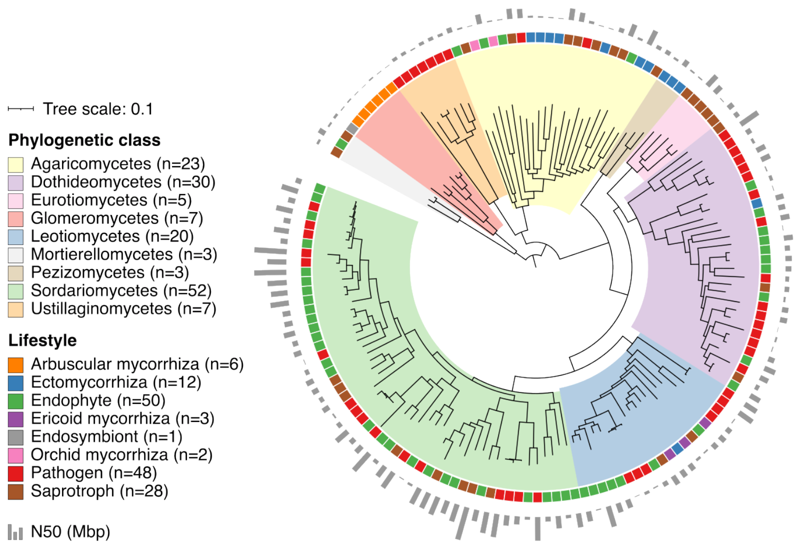

Fig. S4. Description of the dataset of 150 fungal genomes used for comparative genomics. Phylogenomic tree calculated on total sets of proteins from the selected 150 fungi (STAG method (*83*) implemented in OrthoFinder (*81*)). Color ranges on the phylogenomic tree highlight phylogenetic classes and fungal lifestyles are indicated. A barplot shows the N50 values, reflecting overall genome quality and fragmentation.

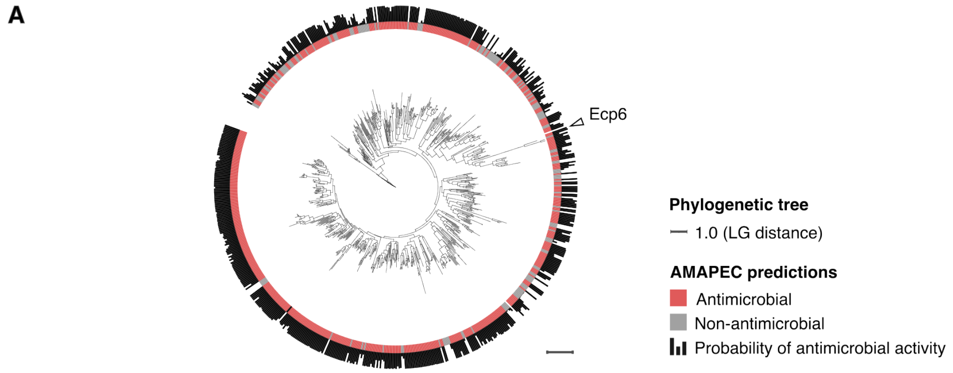

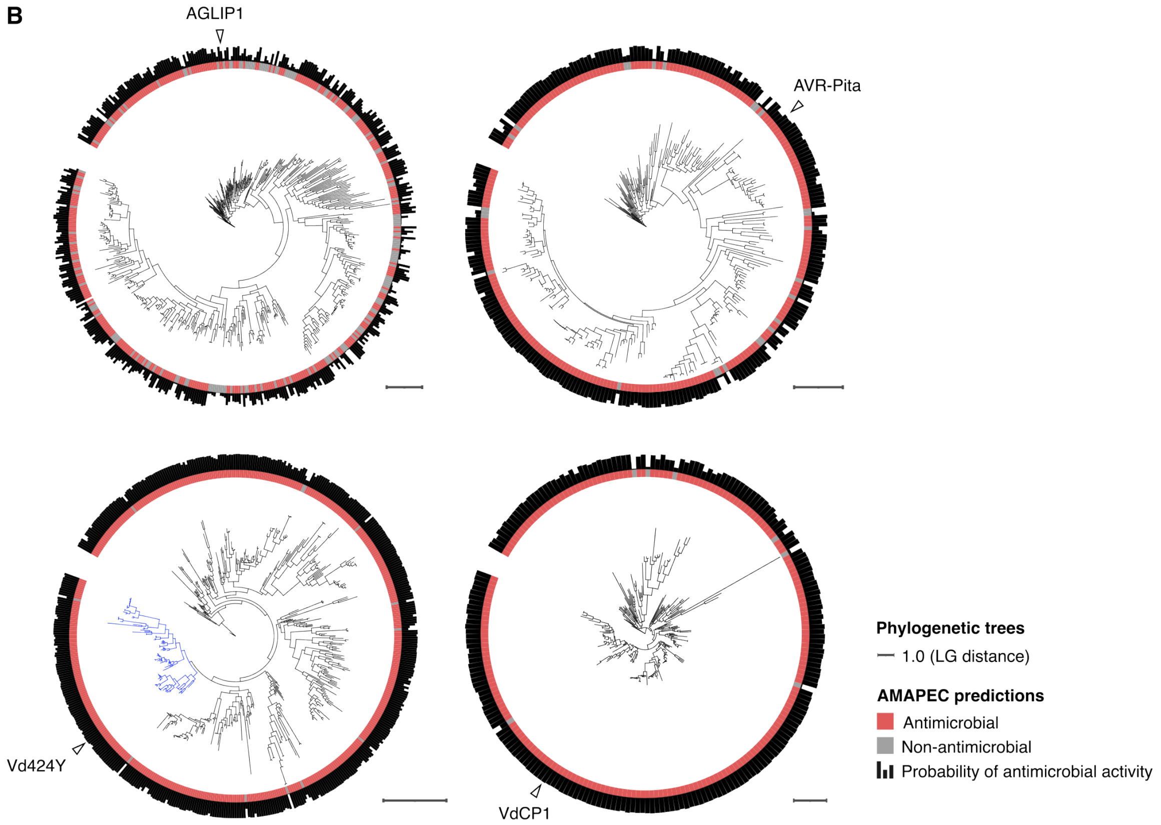

Fig. S5. Phylogenies of five secreted protein families, including reference effectors, with antimicrobial activity prediction for each family member. (**A**) Phylogeny and antimicrobial activity prediction calculated on LysM effectors annotated in the fungal dataset of 150 genomes and additionally in *Cladosporium fulvum*, which encodes the reference Ecp6 effector (*38*). (**B**) Phylogenies of the families of AGLIP1, AVR-Pita, Vd424Y and VdCP1 as defined by orthology prediction in the dataset of 150 fungal genomes. All five phylogenies were calculated by sequence alignment of mature sequence proteins with MAFFT (*90*), and phylogenetic reconstruction with IQ-TREE (*89*) with maximum-likelihood model 'LG'. In the family tree of Vd424Y, the clade depicted in blue was considered as the Vd424Y subfamily and was further analyzed in Fig. 4, A and fig. S17.

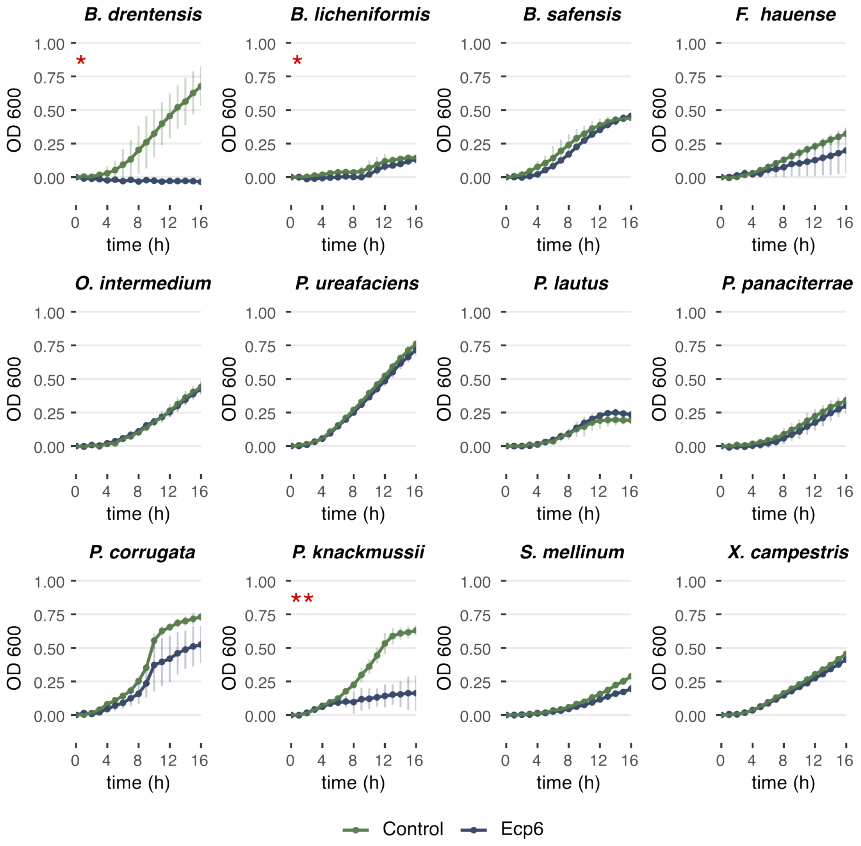

Fig. S6. Selective antibacterial activity displayed *in vitro* by the Ecp6 effector protein of the tomato leaf mold pathogen *Cladosporium fulvum*. Absorbance measurements (at wavelength 600 nm) over 16 hours of bacterial cultivation in presence and absence of 8 µM of heterologously produced effector protein. Each growth curve represents to the mean OD_{600} over 3 independent replicates and error bars correspond to the standard deviation. The assay was performed on a phylogenetically diverse set of 12 bacterial isolates, which species-level phylogeny can be seen on Fig. 3, C. Asterisks highlight significance ($P < 0.05$) of a Student's T-tests computed on area-under-curve values comparing bacterial growth in presence and absence of effector protein: ***: $P < 0.001$, **: $P < 0.01$, *: $P < 0.05$.

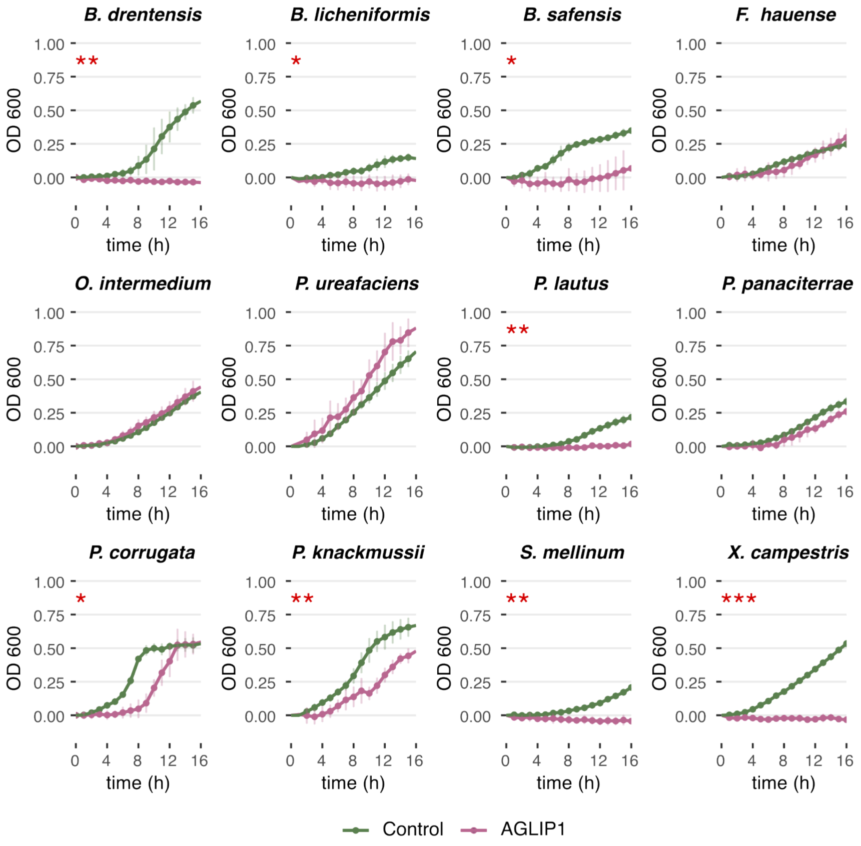

Fig. S7. Selective antibacterial activity displayed *in vitro* displayed by the AGLIP1 effector protein of the root rot pathogen *Rhizoctonia solani*. Absorbance measurements (at wavelength 600 nm) over 16 hours of bacterial cultivation in presence and absence of 8 µM of heterologously produced effector protein. Each growth curve represents to the mean OD_{600} over 3 independent replicates and error bars correspond to the standard deviation. The assay was performed on a phylogenetically diverse set of 12 bacterial isolates, which species-level phylogeny can be seen on Fig. 3, C. Asterisks highlight significance ($P < 0.05$) of a Student's T-tests computed on area-under-curve values comparing bacterial growth in presence and absence of effector protein: ***: $P < 0.001$, **: $P < 0.01$, *: $P < 0.05$.

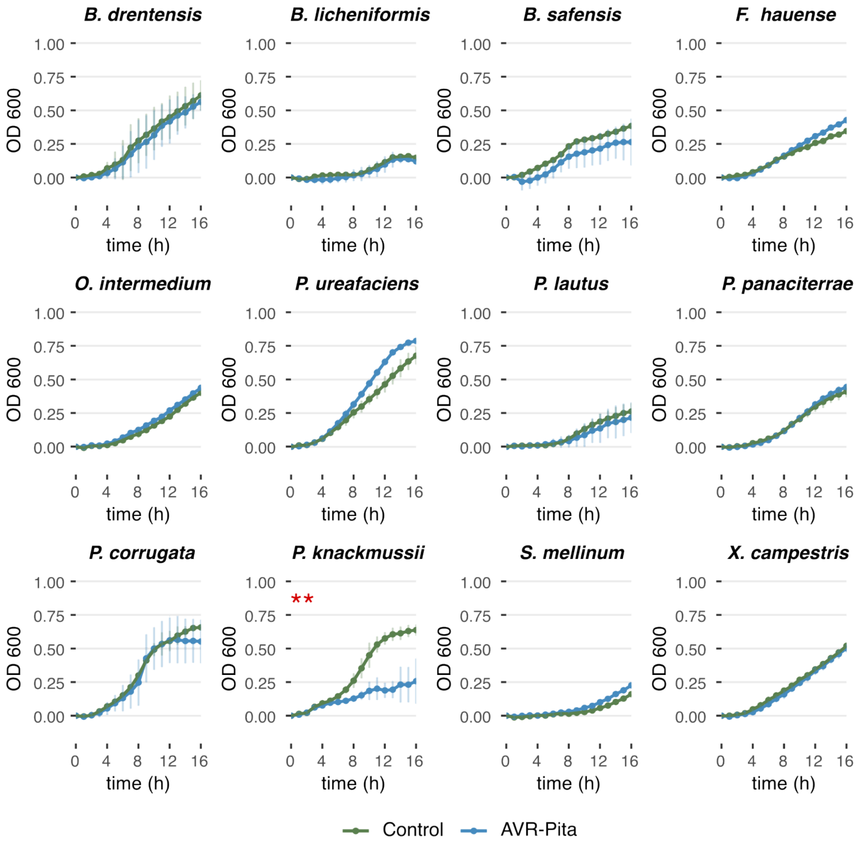

Fig. S8. Selective antibacterial activity displayed *in vitro* by the AVR-Pita effector protein of the rice blast pathogen *Magnaporthe oryzae*. Absorbance measurements (at wavelength 600 nm) over 16 hours of bacterial cultivation in presence and absence of 8 μM of heterologously produced effector protein. Each growth curve represents to the mean OD_{600} over 3 independent replicates and error bars correspond to the standard deviation. The assay was performed on a phylogenetically diverse set of 12 bacterial isolates, which species-level phylogeny can be seen on Fig. 3, C. Asterisks highlight significance ($P < 0.05$) of a Student's T-tests computed on area-under-curve values comparing bacterial growth in presence and absence of effector protein: ***: $P < 0.001$, **: $P < 0.01$, *: $P < 0.05$.

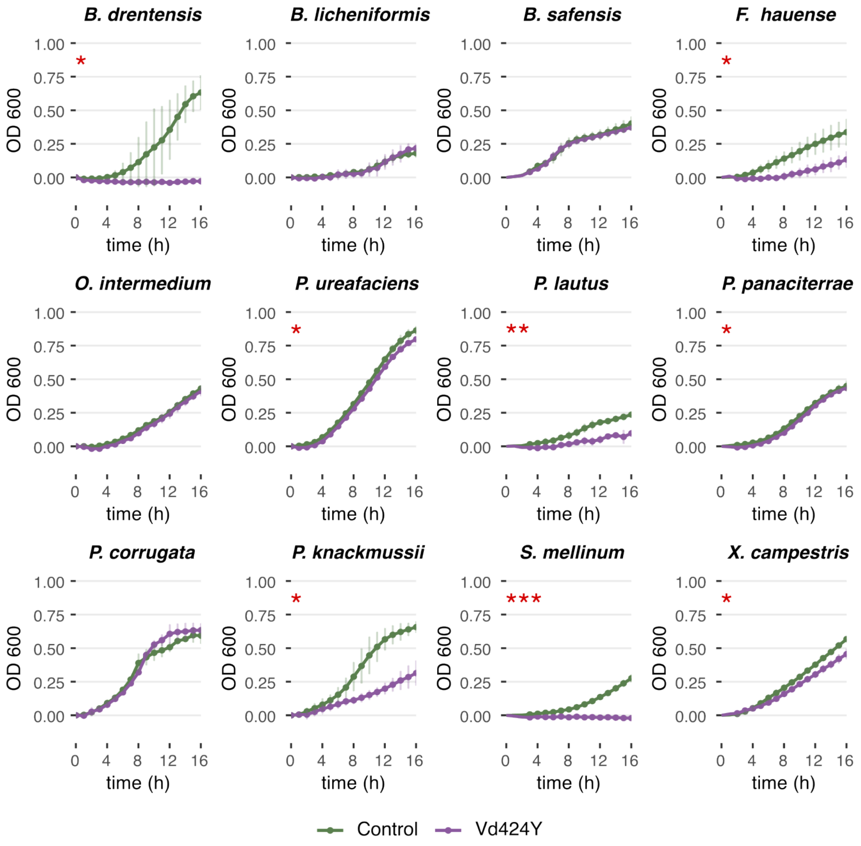

Fig. S9. Selective antibacterial activity displayed *in vitro* by the Vd424Y effector protein of the vascular wilt pathogen *Verticillium dahliae*. Absorbance measurements (at wavelength 600 nm) over 16 hours of bacterial cultivation in presence and absence of 8 µM of heterologously produced effector protein. Each growth curve represents to the mean OD_{600} over 3 independent replicates and error bars correspond to the standard deviation. The assay was performed on a phylogenetically diverse set of 12 bacterial isolates, which species-level phylogeny can be seen on Fig. 3, C. Asterisks highlight significance ($P < 0.05$) of a Student's T-tests computed on area-under-curve values comparing bacterial growth in presence and absence of effector protein: ***: $P < 0.001$, **: $P < 0.01$, *: $P < 0.05$.

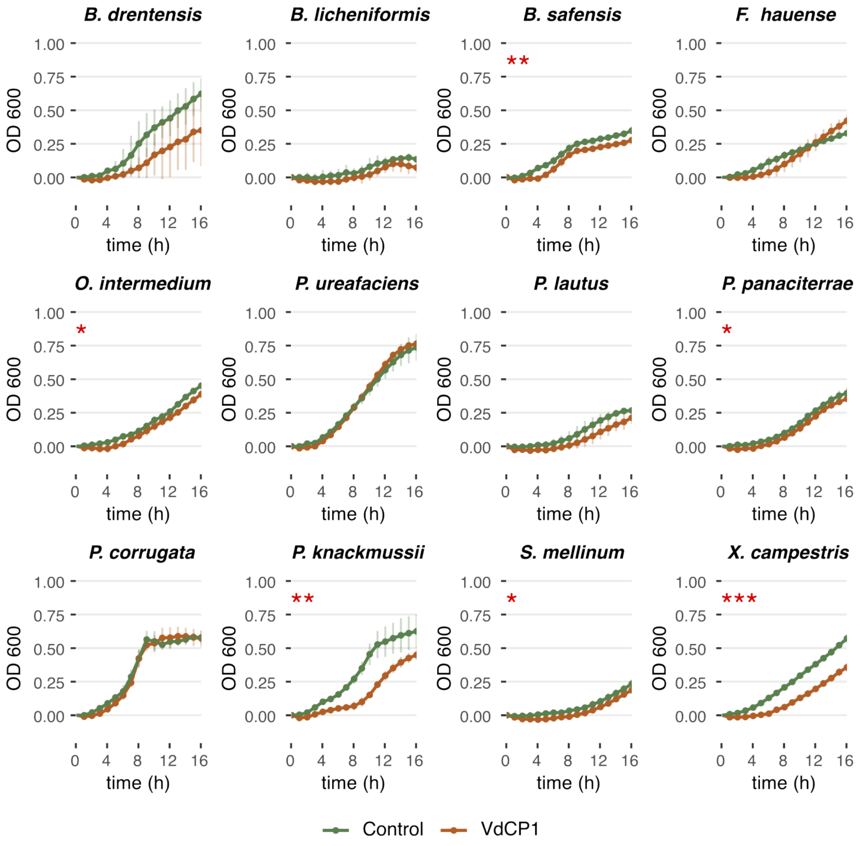

Fig. S10. Selective antibacterial activity displayed *in vitro* by the VdCP1 effector protein of the vascular wilt pathogen *Verticillium dahliae*. Absorbance measurements (at wavelength 600 nm) over 16 hours of bacterial cultivation in presence and absence of 8 µM of heterologously produced effector protein. Each growth curve represents to the mean OD_{600} over 3 independent replicates and error bars correspond to the standard deviation. The assay was performed on a phylogenetically diverse set of 12 bacterial isolates, which species-level phylogeny can be seen on Fig. 3, C. Asterisks highlight significance ($P < 0.05$) of a Student's T-tests computed on area-under-curve values comparing bacterial growth in presence and absence of effector protein: ***: $P < 0.001$, **: $P < 0.01$, *: $P < 0.05$.

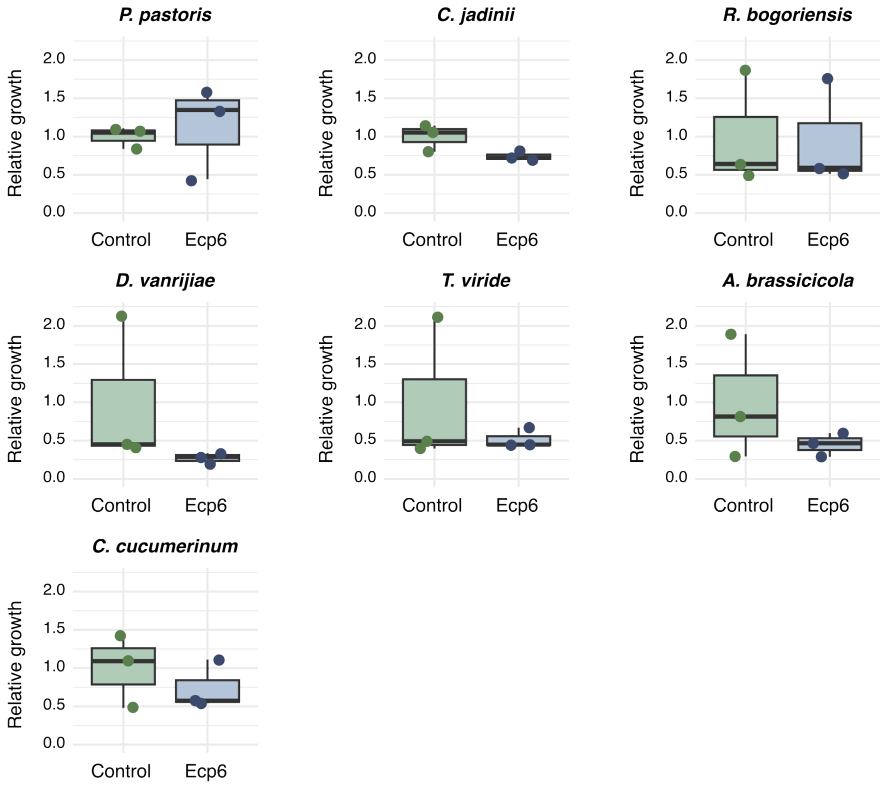

Fig. S11. Absence of antifungal activity displayed *in vitro* by the Ecp6 effector protein of the tomato leaf mold pathogen *Cladosporium fulvum*. Normalized fungal areas measured on microscopy photographs of growth medium, after 16 hours of fungal culture in presence and absence of 8 µM of heterologously produced effector protein. The assay was performed on a phylogenetically diverse set of seven fungal isolates, which species-level phylogeny can be seen on Fig. 3, D. Asterisks highlight significance ($P < 0.05$) of a Student's T-tests computed comparing fungal growth in presence and absence of effector protein: ***: $P < 0.001$, **: $P < 0.01$, *: $P < 0.05$.

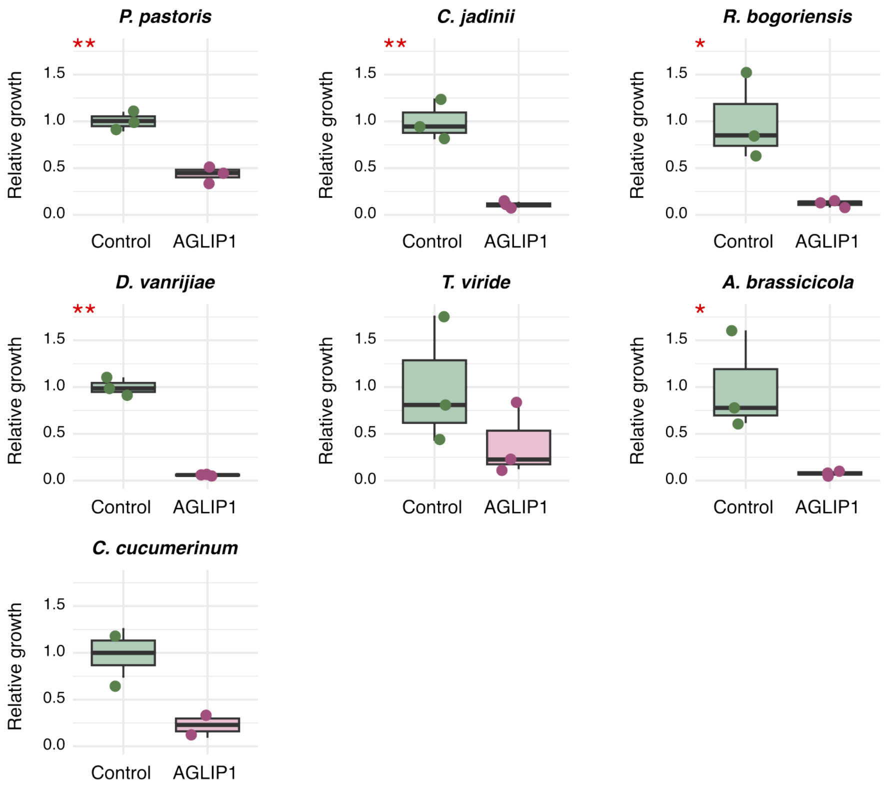

Fig. S12. Antifungal activity displayed *in vitro* by the AGLIP1 effector protein of the root rot pathogen *Rhizoctonia solani*. Normalized fungal areas measured on microscopy photographs of growth medium, after 16 hours of fungal culture in presence and absence of 8 µM of heterologously produced effector protein. The assay was performed on a phylogenetically diverse set of seven fungal isolates, which species-level phylogeny can be seen on Fig. 3, D. Asterisks highlight significance $P < 0.05$) of a Student's T-tests computed comparing fungal growth in presence and absence of effector protein: ***: $P < 0.001$, **: $P < 0.01$, *: $P < 0.05$.

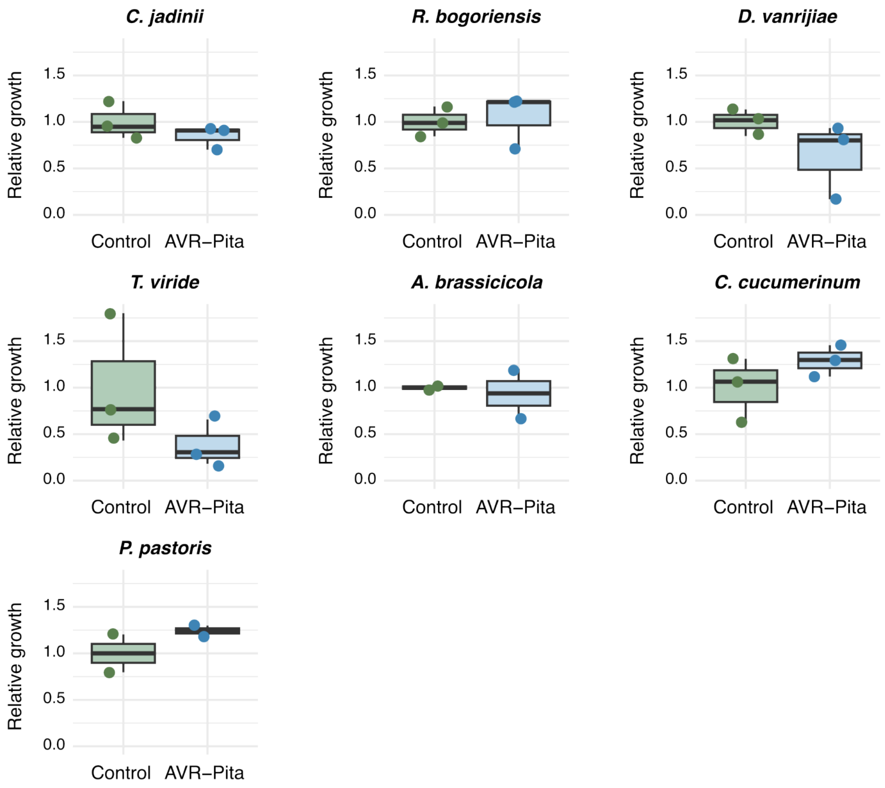

Fig. S13. Absence of antifungal activity displayed *in vitro* by the AVR-Pita effector protein of the rice blast pathogen *Magnaporthe oryzae*. Normalized fungal areas measured on microscopy photographs of growth medium, after 16 hours of fungal culture in presence and absence of 8 µM of heterologously produced effector protein. The assay was performed on a phylogenetically diverse set of seven fungal isolates, which species-level phylogeny can be seen on Fig. 3, D. Asterisks highlight significance ($P < 0.05$) of a Student's T-tests computed comparing fungal growth in presence and absence of effector protein: ***: $P < 0.001$, **: $P < 0.01$, *: $P < 0.05$.

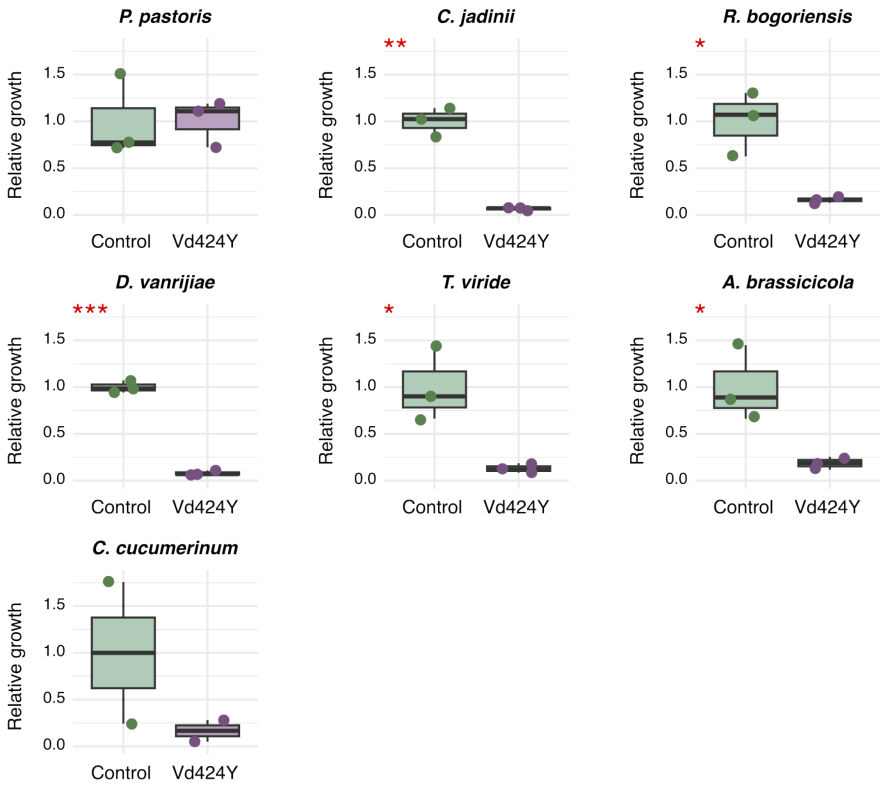

Fig. S14. Antifungal activity displayed *in vitro* by the Vd424Y effector protein of the vascular wilt pathogen *Verticillium dahliae*. Normalized fungal areas measured on microscopy photographs of growth medium, after 16 hours of fungal culture in presence and absence of 8 µM of heterologously produced effector protein. The assay was performed on a phylogenetically diverse set of seven fungal isolates, which species-level phylogeny can be seen on Fig. 3, D. Asterisks highlight significance ($P < 0.05$) of a Student's T-tests computed comparing fungal growth in presence and absence of effector protein: ***: $P < 0.001$, **: $P < 0.01$, *: $P < 0.05$.

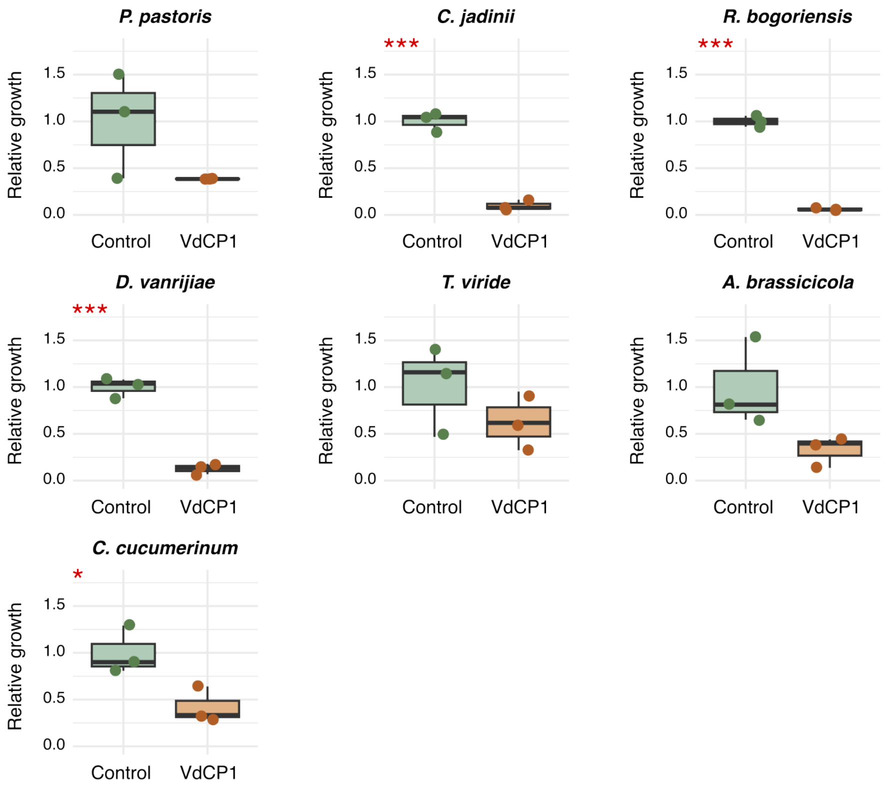

Fig. S15. Antifungal activity displayed *in vitro* by the VdCP1 effector protein of the vascular wilt pathogen *Verticillium dahliae*. Normalized fungal areas measured on microscopy photographs of growth medium, after 16 hours of fungal culture in presence and absence of 8 µM of heterologously produced effector protein. The assay was performed on a phylogenetically diverse set of seven fungal isolates, which species-level phylogeny can be seen on Fig. 3, D. Asterisks highlight significance ($P < 0.05$) of a Student's T-tests computed comparing fungal growth in presence and absence of effector protein: ***: $P < 0.001$, **: $P < 0.01$, *: $P <$ 0.05.

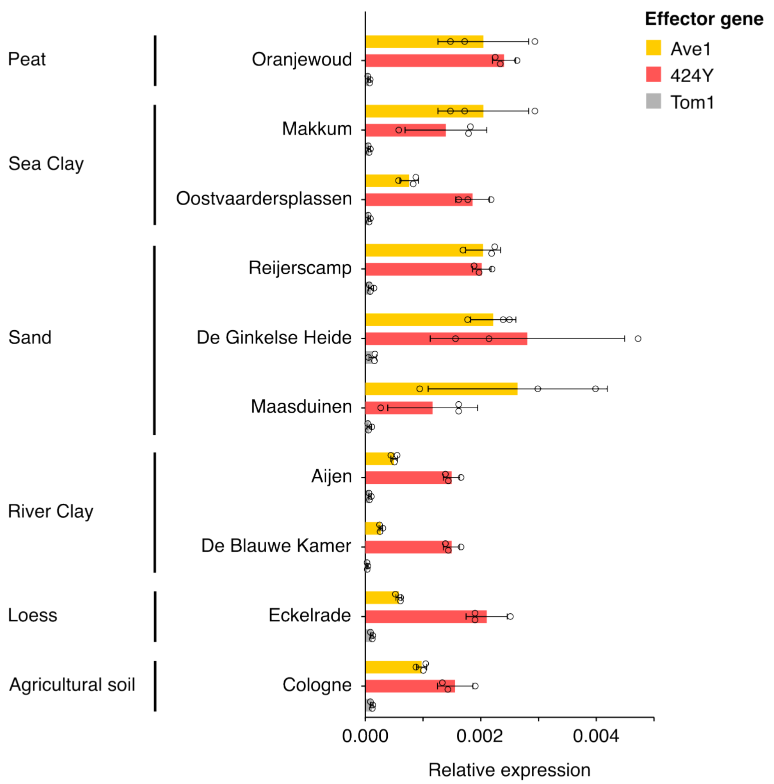

Fig. S16. Expression of the *V. dahliae* 424Y-encoding gene in a diverse set of soils. Real-time PCR measurements of effector gene expression in a diverse set of 10 soils (*95*), classified by soil type (left). In addition to the 424Y-encoding gene, effector genes Ave1 (*7*) and Tom1 (*96*) were studied and serve as positive and negative controls, respectively.

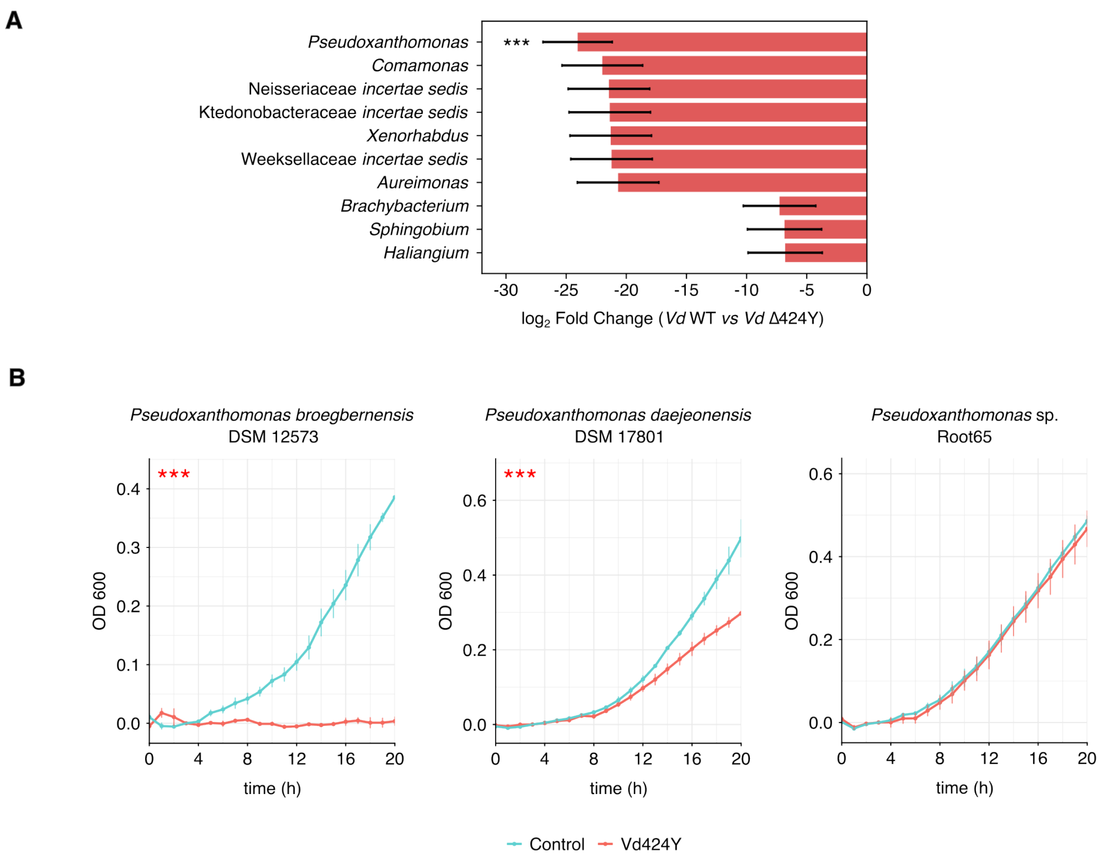

Fig. S17. Candidate bacterial targets of Vd424Y in tomato plant microbiota. (**A**) Barplot showing the $\log_2$-transformed fold change values and standard errors of the 10 bacterial genera that are the most depleted in presence of the *Vd424Y* gene in *Verticillium dahliae*-infected plant tissues. These data were obtained by applying the method DESeq2 on the number of reads assigned to each bacterial genus in three plant samples per condition. (**B**) Absorbance measurements (at wavelength 600 nm) over 20 hours of bacterial cultivation in presence and absence of 8 µM of heterologously produced Vd424Y protein. Each growth curve represents to the mean OD_{600} over 3 replicates and error bars correspond to the standard deviation. This assay was performed on three bacterial isolates from genus *Pseudoxanthomonas*. Asterisks highlight significance ($P < 0.05$) of a Student's T-tests computed on area-under-curve values comparing bacterial growth in presence and absence of effector protein: ***: $P < 0.001$, **: $P < 0.01$, *: $P < 0.05$.

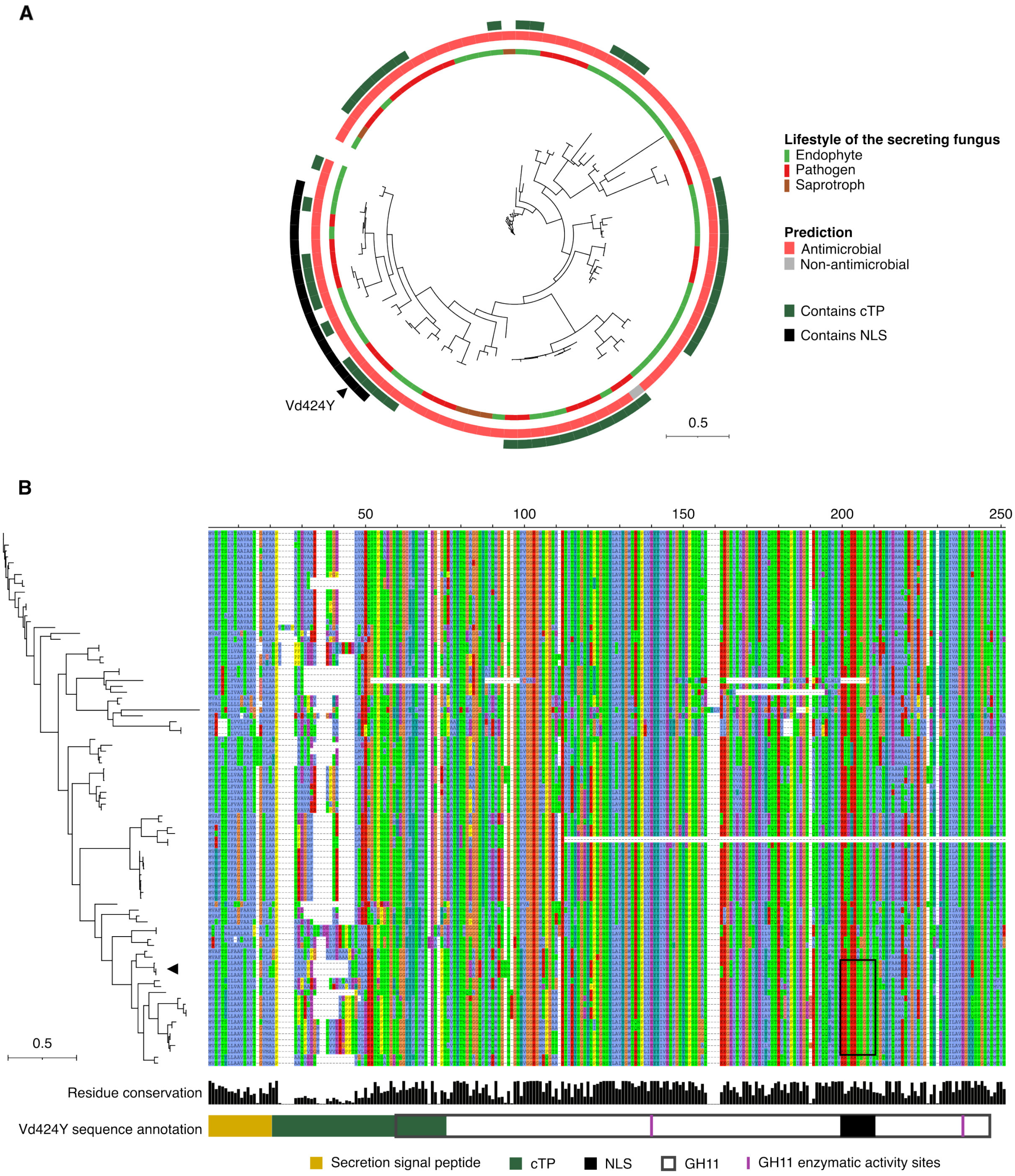

Fig. S18. Evolution of Vd424Y sequence features. (**A**) Maximum-likelihood phylogenetic tree (computed with IQ-TREE (*89*), model 'LG') of Vd424Y homologs in a dataset of 150 fungal genomes. The set of proteins represent those occurring in the same subfamily (large clade) as Vd424Y, as identified in the total family phylogenetic tree (fig. S5). The tree was manually rooted at the protein identified as an optimal outgroup by IQ-TREE and annotated with the lifestyles of the fungus secreting each protein, the results of antimicrobial activity as well as the occurrence of a chloroplast transit peptide (cTP) and nuclear localization signal (NLS) annotated using ChloroP (*93*) and cNLS Mapper (*94*), respectively. (**B**) The same phylogenetic tree as on panel A is displayed on the

left, with a black triangle indicating the location of Vd424Y. On the right, the protein sequence alignment (generated with MAFFT (*90*)) presents sequence variation in the Vd424Y subfamily. A black rectangle circumscribes annotated NLS motifs in the sequences. At the bottom, a barplot presents the conservation of amino acids at each position in the protein sequence. Below, a diagram shows the organization in functional domains of the Vd424Y sequence, as characterized previously (*33*).

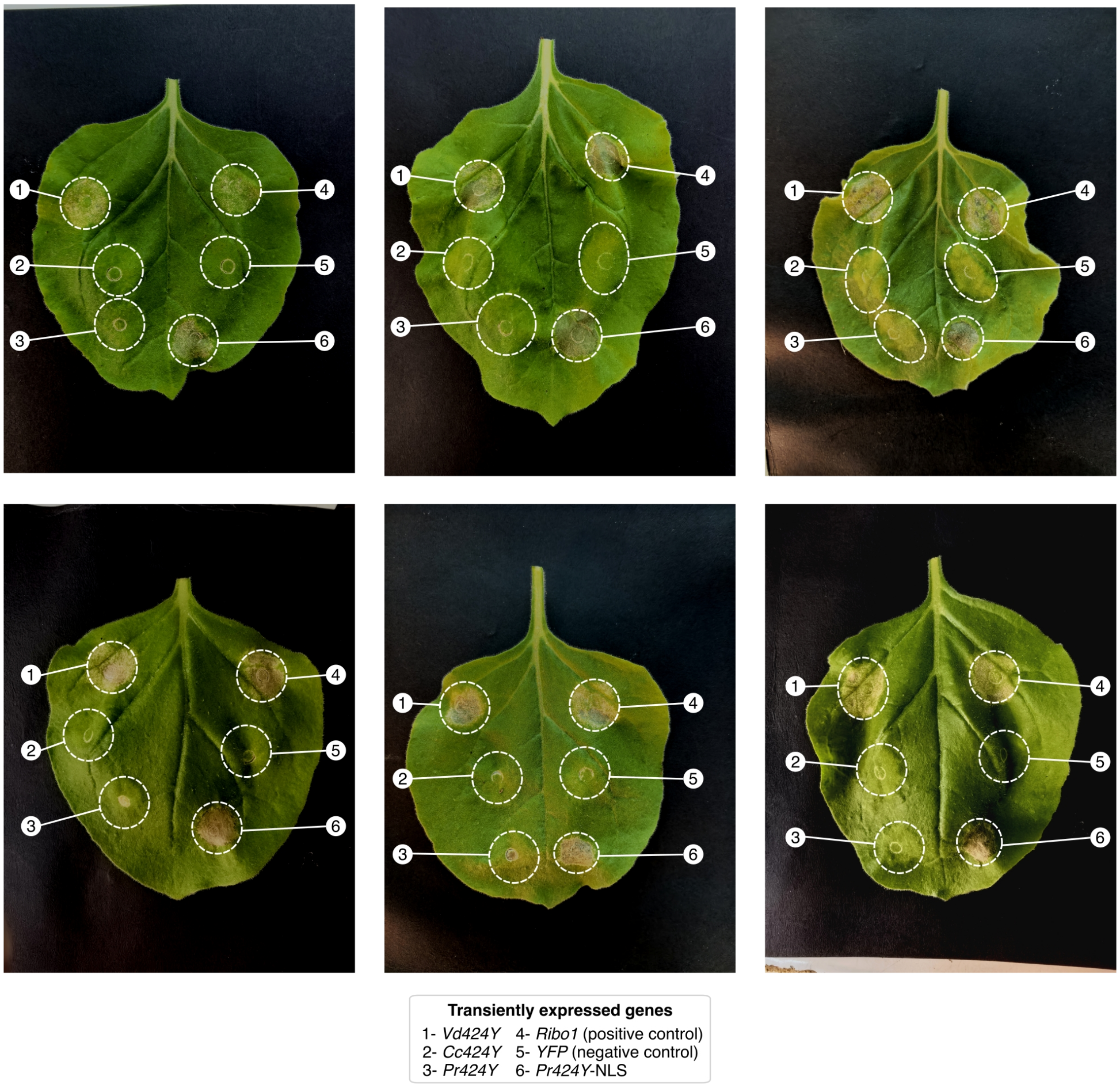

Fig. S19. *Nicotiana benthamiana* leaves transiently expressing Vd424Y and its homologs. Photographs showing the phenotypes of *N. benthamiana* leaves infiltrated with engineered *Agrobacterium tumefaciens* strains to mediate the transient expression of effector genes *Vd424Y* from *Verticillium dahliae* (1), *Cc424Y* from *Coprinopsis cinerea* (2), *Pr424Y* from *Penicillium restrictum* (3), *Ribo1* from *Ustilago maydis* (*11*) as a positive control (4), *YFP* as a negative control (5) and a chimeric gene corresponding to *Pr424Y* that carries the NLS of *Vd424Y* (6). Leaf areas that were infiltrated are circled by dotted white lines and annotated with the names of the genes that were transiently expressed. These six photos present replicates of the experiment shown in Fig. 5, B.

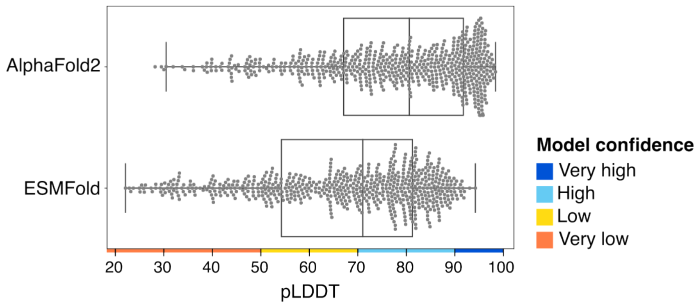

Fig. S20. Confidence of AlphaFold2- and ESMFold-predicted structures for secreted proteins of *Verticillium dahliae*. Boxplots showing the distribution of mean pLDDT confidence scores of AlphaFold2 (*60*)- and ESMFold (*71*)-predicted structures for 626 non-CAZyme secreted proteins of *Verticillium dahliae*. While the secretome of *V. dahliae* includes 635 non-CAZyme proteins, AlphaFold2 failed at predicting the structures of nine of these proteins, which were therefore excluding from this analysis. The color code depicting model confidence originates from the documentation of AlphaFold2.

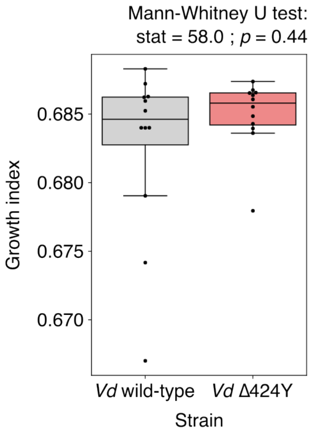

Fig. S21. Growth of *Verticillium dahliae* wild-type and Δ424Y mutant *in vitro*. Boxplot showing growth indices of *Verticillium dahliae* JR2 wild-type and Δ424Y calculated from quantitative PCR Ct values (see Material and methods for details) after 48 hours in growth medium. In total, 12 samples per condition corresponding to 12 independent fungal cultures over 3 biological replicates were analyzed. Results of a Mann-Whitney U test revealing no significant difference between the wild-type and the mutant strain are written on top of the figure.

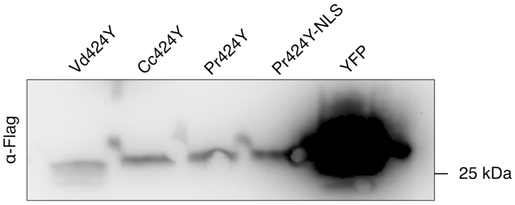

Fig. S22. Expression of 424Y proteins in *Nicotiana benthamiana* leaves. Western blot analysis confirming the expression of Vd424Y, Cc424Y, Pr424Y, Pr424Y-NLS (a Pr424Y chimeric protein carrying the nuclear localization signal of Vd424Y), and YFP (positive control) in *Nicotiana benthamiana* leaves following *Agrobacterium tumefaciens*-mediated transformation.

Table S1. Description of the literature-curated set of antimicrobial proteins.

For each protein in the set, the table provides (1) a reference identifier; (2) the name of the protein in literature; (3) the reported antimicrobial activity in literature; (4) the phylogenetic group of organisms by which it is encoded; (5) the species producing the protein; (6) the publication that described the antimicrobial activity of this protein; (7) whether a secretion signal was identified by SignalP (*52*) and removed from the protein sequence; (8) the pLDDT confidence score for the AlphaFold2 (*60*)-predicted structure.

Table S2. Description of the negative training set of presumably non-antimicrobial proteins.

For each protein in the set, the table provides (1) a reference identifier; (2) the functional description of the protein in the UniProt database (*58*); (3) the phylogenetic group of organisms encoding the protein; (4) the species producing the protein; (5) the UniProt entry identifier; (6) whether a secretion signal was identified by SignalP (*52*) and removed from the protein sequence; (7) the pLDDT confidence score for the AlphaFold2 (*60*)-predicted structure.

Table S3. Properties and k-mers describing protein physicochemistry used to predict antimicrobial activity.

List of 70 properties calculated from protein sequences and structures, with as a reference, the method implementing the calculation or publication introducing the formula. Additionally, the list includes 6 k-mers (in a reduced amino acid alphabet designed based on amino acid properties, see Material and Methods) found to be over- or under-represented in antimicrobial protein sequences. All 76 properties are used by the predictor to classify a protein as antimicrobial or non-antimicrobial.

Table S4. Prediction of antimicrobial activity for six recently characterized fungal antimicrobial proteins.

List of six recently characterized fungal antimicrobial proteins with their organism of origin, reference publication and sequence, together with the results of antimicrobial activity prediction after structure prediction with ESMFold (*71*).

Table S5. Functional annotation of the secretome of the arbuscular mycorrhizal glomeromycete *Rhizophagus irregularis* and antimicrobial activity prediction results.

Secretome functional annotation outputs of emapper (*77*), transmembrane protein prediction by TMBed (*80*) and carbohydrate-active enzyme annotation from dbCAN (*79*), together with the results of antimicrobial activity prediction with AMAPEC.

Table S6. Functional annotation of the secretome of the saprotrophic basidiomycete *Coprinopsis cinerea* and antimicrobial activity prediction results.

Secretome functional annotation outputs of emapper (*77*), transmembrane protein prediction by TMBed (*80*) and carbohydrate-active enzyme annotation from dbCAN (*79*), together with the results of antimicrobial activity prediction with AMAPEC.

Table S7. Functional annotation of the secretome of the saprotrophic ascomycete *Verticillium dahliae* and antimicrobial activity prediction results.

Secretome functional annotation outputs of emapper (*77*), transmembrane protein prediction by TMBed (*80*) and carbohydrate-active enzyme annotation from dbCAN (*79*), together with the results of antimicrobial activity prediction with AMAPEC.

Table S8. Description of the 150-fungal genome dataset used for comparative genomic analyses.

List of the fungal genomes included in the comparative genomic dataset used throughout this study. This dataset is a modified version of a previously studied set of 120 fungal genomes (*82*). This table provides genome identifiers, strain names, assigned lifestyles, metadata about the fungal isolation, genome references, statistics about the genome size and quality, and fungal phylogeny (phylum, class, order).

Table S9. Annotation and conservation of secreted protein families defined through orthology prediction.

List of secreted protein families defined through orthology prediction by OrthoFinder (*81*), together with the functional annotation of their most central, representative member (identified with phylorep (*84*)) including transmembrane protein prediction (TMBed (*80*)) and CAZyme annotation (dbcan (*79*)). This table also provides results of antimicrobial activity prediction for protein family representive members which were not annotated as transmembrane or CAZymes.

Table S10. Antimicrobial activity prediction for secreted proteins of the vascular wilt pathogen *Verticillium dahliae* which were previously studied for their contribution to fungal virulence.

List of proteins in the secretome of the plant pathogen *Verticillium dahliae* that match (blastp-based identification, sequence identity >95%) proteins registered in PHI-base (*37*) that were previously studied for their contribution to fungal virulence. This table provides results of antimicrobial activity prediction for these proteins, following structure prediction with ESMFold (*71*).

Table S11. Antimicrobial activity prediction for previously characterized fungal effectors registered in PHI-base.

List of fungal proteins registered as "effectors (plant avirulence determinant)" in PHI-base (*37*), together with their antimicrobial activity prediction, their closest protein homolog in the 150-genome dataset and their conservation throughout this dataset.

Table S12. Prediction of antimicrobial activities in LysM effectors annotated in 151 fungal genomes.

LysM effectors annotated in the set of 150 fungal genomes ((*35*), see Material and Methods for details), as well as in the genome of *Cladosporium fulvum* which secretes the well-studied LysM effector Ecp6 (*38*), listed with their LysM domain identities (as defined by InterPro (*91*)), sequences and results of antimicrobial activity prediction after structure prediction with ESMFold (*71*).

Table S13. Prediction of antimicrobial activities in the protein family including the AGLIP1 effector protein of the root rot pathogen *Rhizoctonia solani*.

List of protein homologs of AGLIP1 (effector secreted by *Rhizoctonia solani*) identified and classified in the same family through orthology prediction by OrthoFinder (*81*), together with results of antimicrobial activity prediction following structure prediction with ESMFold (*71*).

Table S14. Prediction of antimicrobial activities in the protein family including the AVR-Pita effector protein of the rice blast pathogen *Magnaporthe oryzae*.

List of protein homologs of AVR-Pita (effector secreted by *Magnaporthe oryzae*) identified and classified in the same family through orthology prediction by OrthoFinder (*81*)), together with results of antimicrobial activity prediction following structure prediction with ESMFold (*71*).

Table S15. Prediction of antimicrobial activities in the protein family including the Vd424Y effector protein of the vascular wilt pathogen *Verticillium dahliae*.

List of protein homologs of Vd424Y (effector secreted by *Verticillium dahliae*) identified and classified in the same family through orthology prediction by OrthoFinder (*81*), together with results of antimicrobial activity prediction following structure prediction with ESMFold (*71*).

Table S16. Prediction of antimicrobial activities in the protein family including the VdCP1 effector protein of the vascular wilt pathogen *Verticillium dahliae*.

List of protein homologs of VdCP1 (effector secreted by *Verticillium dahliae*) identified and classified in the same family through orthology prediction by OrthoFinder (*81*), together with results of antimicrobial activity prediction following structure prediction with ESMFold (*71*).

Table S17. Compositions of dialysis buffers used for effector protein purification.

List and composition of dialysis buffers used to purify effector proteins AGLIP1, AVR-Pita, Vd424Y and VdCP1.

Table S18. Sequences of 424Y proteins.

Gene loci and amino acid sequences of proteins Vd424Y, Cc424Y, Pr424Y and Pr424Y-NLS.

REFERENCES

1. D. E. Cook, C. H. Mesarich, B. P. H. J. Thomma, Understanding plant immunity as a surveillance system to detect invasion. *Annu. Rev. Phytopathol.* **53**, 541–563 (2015).

2. G. Z. Han, Origin and evolution of the plant immune system. *New Phytol.* **222**, 70–83 (2019).

3. J. D. G. Jones, B. J. Staskawicz, J. L. Dangl, The plant immune system: From discovery to deployment. *Cell* **187**, 2095–2116 (2024).

4. V. Müller, R. J. de Boer, S. Bonhoeffer, E. Szathmáry, An evolutionary perspective on the systems of adaptive immunity. *Biol. Rev.* **93**, 505–528 (2018).

5. I. Stergiopoulos, P. J. G. M. De Wit, Fungal effector proteins. *Annu. Rev. Phytopathol.* **47**, 233–263 (2009).

6. G. Doehlemann, B. Ökmen, W. Zhu, A. Sharon, Plant pathogenic fungi. *Microbiol. Spectr.* **5**, 10.1128/microbiolspec.FUNK-0023-2016 (2017).

7. N. C. Snelders, H. Rovenich, G. C. Petti, M. Rocafort, G. C. M. van den Berg, J. A. Vorholt, J. R. Mesters, M. F. Seidl, R. Nijland, B. P. H. J. Thomma, Microbiome manipulation by a soil-borne fungal plant pathogen using effector proteins. *Nat. Plants* **6**, 1365–1374 (2020).

8. N. C. Snelders, G. C. Petti, G. C. M. van den Berg, M. F. Seidl, B. P. H. J. Thomma, An ancient antimicrobial protein co-opted by a fungal plant pathogen for in planta mycobiome manipulation. *Proc. Natl. Acad. Sci. U.S.A.* **118**, e2110968118 (2021).

9. N. C. Snelders, J. C. Boshoven, Y. Song, N. Schmitz, G. L. Fiorin, H. Rovenich, G. C. M. van den Berg, D. E. Torres, G. C. Petti, Z. Prockl, L. Faino, M. F. Seidl, B. P. H. J. Thomma, A highly polymorphic effector protein promotes fungal virulence through suppression of plant-associated Actinobacteria. *New Phytol.* **237**, 944–958 (2023).

10. E. A. Chavarro-Carrero, N. C. Snelders, D. E. Torres, A. Kraege, A. López-Moral, G. C. Petti, W. Punt, J. Wieneke, R. García-Velasco, C. J. López-Herrera, M. F. Seidl, B. P. H. J.

Thomma, The soil-borne white root rot pathogen *Rosellinia necatrix* expresses antimicrobial proteins during host colonization. *PLOS Pathog.* **20**, e1011866 (2024).

11. B. Ökmen, P. Katzy, L. Huang, R. Wemhöner, G. Doehlemann, A conserved extracellular Ribo1 with broad-spectrum cytotoxic activity enables smut fungi to compete with host-associated bacteria. *New Phytol.* **240**, 1976–1989 (2023).

12. D. Gómez-Pérez, M. Schmid, V. Chaudhry, Y. Hu, A. Velic, B. Maček, J. Ruhe, A. Kemen, E. Kemen, Proteins released into the plant apoplast by the obligate parasitic protist *Albugo* selectively repress phyllosphere-associated bacteria. *New Phytol.* **239**, 2320–2334 (2023).

13. H. X. Chang, Z. A. Noel, M. I. Chilvers, A β-lactamase gene of *Fusarium oxysporum* alters the rhizosphere microbiota of soybean. *Plant J.* **106**, 1588–1604 (2021).

14. A. Kraege, W. Punt, A. Doddi, J. Zhu, N. Schmitz, N. C. Snelders, B. P. H. J. Thomma, Undermining the cry for help: The phytopathogenic fungus *Verticillium dahliae* secretes an antimicrobial effector protein to undermine host recruitment of antagonistic *Pseudomonas* bacteria. *New Phytol.* **249**, 406–417 (2026).

15. M. Möller, E. H. Stukenbrock, Evolution and genome architecture in fungal plant pathogens. *Nat. Rev. Microbiol.* **15**, 756–771 (2017).

16. L. J. Ma, H. C. Van Der Does, K. A. Borkovich, J. J. Coleman, M. J. Daboussi, A. Di Pietro, M. Dufresne, M. Freitag, M. Grabherr, B. Henrissat, P. M. Houterman, S. Kang, W. B. Shim, C. Woloshuk, X. Xie, J. R. Xu, J. Antoniw, S. E. Baker, B. H. Bluhm, A. Breakspear, D. W. Brown, R. A. E. Butchko, S. Chapman, R. Coulson, P. M. Coutinho, E. G. J. Danchin, A. Diener, L. R. Gale, D. M. Gardiner, S. Goff, K. E. Hammond-Kosack, K. Hilburn, A. Hua-Van, W. Jonkers, K. Kazan, C. D. Kodira, M. Koehrsen, L. Kumar, Y. H. Lee, L. Li, J. M. Manners, D. Miranda-Saavedra, M. Mukherjee, G. Park, J. Park, S. Y. Park, R. H. Proctor, A. Regev, M. C. Ruiz-Roldan, D. Sain, S. Sakthikumar, S. Sykes, D. C. Schwartz, B. G. Turgeon, I. Wapinski, O. Yoder, S. Young, Q. Zeng, S. Zhou, J. Galagan, C. A. Cuomo, H. C. Kistler, M. Rep, Comparative genomics reveals mobile pathogenicity chromosomes in *Fusarium*. *Nature* **464**, 367–373 (2010).

17. R. de Jonge, M. D. Bolton, A. Kombrink, G. C. M. Van Den Berg, K. A. Yadeta, B. P. H. J. Thomma, Extensive chromosomal reshuffling drives evolution of virulence in an asexual pathogen. *Genome Res.* **23**, 1271–1282 (2013).

18. Y. Sato, R. Bex, G. C. M. van den Berg, P. Santhanam, M. Höfte, M. F. Seidl, B. P. H. J. Thomma, Starship giant transposons dominate plastic genomic regions in a fungal plant pathogen and drive virulence evolution. *Nat. Commun.* **16**, 1–17 (2025).

19. M. Torrent, D. Andreu, V. M. Nogués, E. Boix, Connecting peptide physicochemical and antimicrobial properties by a rational prediction model. *PLOS ONE* **6**, e16968 (2011).

20. G. Wang, The antimicrobial peptide database is 20 years old: Recent developments and future directions. *Protein Sci.* **32**, e4778 (2023).

21. F. Wan, F. Wong, J. J. Collins, C. de la Fuente-Nunez, Machine learning for antimicrobial peptide identification and design. *Nat. Rev. Bioeng.* **2**, 392–407 (2024).

22. R. Eichfeld, L. K. Mahdi, C. De Quattro, L. Armbruster, A. B. Endeshaw, S. Miyauchi, M. J. Hellmann, S. Cord-Landwehr, D. Peterson, V. Singan, K. Lail, E. Savage, V. Ng, I. V. Grigoriev, G. Langen, B. M. Moerschbacher, A. Zuccaro, Transcriptomics reveal a mechanism of niche defense: Two beneficial root endophytes deploy an antimicrobial GH18-CBM5 chitinase to protect their hosts. *New Phytol.* **244**, 980–996 (2024).

23. F. Chen, L. Ou, H. Wu, L. Huang, Y.-P. Chen, "Expression and characterization of the antifungal protein PtAFP from *Pyrenophora tritci-repentis* by synonymous codon bias in *Escherichia coli*," in *Proc. SPIE 13208*, *Third Int. Conf. Biomed. Intell. Syst.* (*IC-BIS 2024*) (2024), vol. 13208, pp. 13–19.

24. K. de Guillen, L. Mammri, J. Gracy, A. Padilla, P. Barthe, F. Hoh, M. Lahfa, J. Rouffet, Y. Petit-Houdenot, T. Kroj, M.-H. Lebrun, *Zymoseptoria tritici* effectors structurally related to killer proteins UmV-KP4 and UmV-KP6 inhibit fungal growth, and define extended protein families in fungi. *Mol. Plant Pathol.* **26**, e70141 (2025).

25. Z. Sorger, P. Sengupta, K. Beier-Heuchert, J. Bautor, J. E. Parker, E. Kemen, G. Doehlemann, GH25 lysozyme mediates tripartite interkingdom interactions and microbial competition on the plant leaf surface. *Proc. Natl. Acad. Sci. U.S.A.* **122**, e2510124122 (2025).

26. L. Florez, V. M. Flores-Núñez, C. S. Francisco, E. Holtgrewe Stukenbrock, The fungal effector AvrStb6 regulates the wheat pathobiome. *Zenodo* (2025). https://doi.org/10.5281/zenodo.15852925.

27. F. Mesny, AMAPEC v1.0. *Zenodo* (2026). https://doi.org/10.5281/zenodo.18220951.

28. E. F. Fradin, B. P. H. J. Thomma, Physiology and molecular aspects of *Verticillium* wilt diseases caused by *V. dahliae* and *V. albo-atrum*. *Mol. Plant Pathol.* **7**, 71–86 (2006).

29. N. C. Snelders, H. Rovenich, B. P. H. J. Thomma, Microbiota manipulation through the secretion of effector proteins is fundamental to the wealth of lifestyles in the fungal kingdom. *FEMS Microbiol. Rev.* **46**, fuac022 (2022).

30. G. J. Kettles, C. Bayon, C. A. Sparks, G. Canning, K. Kanyuka, J. J. Rudd, Characterization of an antimicrobial and phytotoxic ribonuclease secreted by the fungal wheat pathogen *Zymoseptoria tritici*. *New Phytol.* **217**, 320–331 (2018).

31. Y. Zhang, Y. Gao, Y. Liang, Y. Dong, X. Yang, J. Yuan, D. Qiu, The *Verticillium dahliae* SnodProt1-like protein VdCP1 contributes to virulence and triggers the plant immune system. *Front. Plant Sci.* **8**, 1880 (2017).

32. L. Liu, Z. Wang, J. Li, Y. Wang, J. Yuan, J. Zhan, P. Wang, Y. Lin, F. Li, X. Ge, *Verticillium dahliae* secreted protein Vd424Y is required for full virulence, targets the nucleus of plant cells, and induces cell death. *Mol. Plant Pathol.* **22**, 1109–1120 (2021).

33. D. Wang, J. Y. Chen, J. Song, J. J. Li, S. J. Klosterman, R. Li, Z. Q. Kong, K. V. Subbarao, X. F. Dai, D. D. Zhang, Cytotoxic function of xylanase VdXyn4 in the plant vascular wilt pathogen *Verticillium dahliae*. *Plant Physiol.* **187**, 409–429 (2021).

34. A. Kombrink, H. Rovenich, X. Shi-Kunne, E. Rojas-Padilla, G. C. M. van den Berg, E. Domazakis, R. de Jonge, D. J. Valkenburg, A. Sánchez-Vallet, M. F. Seidl, B. P. H. J.

Thomma, *Verticillium dahliae* LysM effectors differentially contribute to virulence on plant hosts. *Mol. Plant Pathol.* **18**, 596–608 (2017).

35. R. de Jonge, B. P. H. J. Thomma, Fungal LysM effectors: Extinguishers of host immunity? *Trends Microbiol.* **17**, 151–157 (2009).

36. A. Kombrink, B. P. H. J. Thomma, LysM effectors: Secreted proteins supporting fungal life. *PLOS Pathog.* **9**, e1003769 (2013).

37. M. Urban, A. Cuzick, J. Seager, V. Wood, K. Rutherford, S. Y. Venkatesh, J. Sahu, S. Vijaylakshmi Iyer, L. Khamari, N. De Silva, M. C. Martinez, H. Pedro, A. D. Yates, K. E. Hammond-Kosack, PHI-base in 2022: A multi-species phenotype database for pathogen–host Interactions. *Nucleic Acids Res.* **50**, D837–D847 (2022).

38. R. de Jonge, H. P. Van Esse, A. Kombrink, T. Shinya, Y. Desaki, R. Bours, S. Van Der Krol, N. Shibuya, M. H. A. J. Joosten, B. P. H. J. Thomma, Conserved fungal LysM effector Ecp6 prevents chitin-triggered immunity in plants. *Science* **329**, 953–955 (2010).

39. S. Li, X. Peng, Y. Wang, K. Hua, F. Xing, Y. Zheng, W. Liu, W. Sun, S. Wei, The effector AGLIP1 in *Rhizoctonia solani* AG1 IA triggers cell death in plants and promotes disease development through inhibiting PAMP-triggered immunity in *Arabidopsis thaliana*. *Front. Microbiol.* **10**, 2228 (2019).

40. G. Xiao, N. Laksanavilat, S. Cesari, K. Lambou, M. Baudin, A. Jalilian, M. J. Telebanco-Yanoria, V. Chalvon, I. Meusnier, E. Fournier, D. Tharreau, B. Zhou, J. Wu, T. Kroj, The unconventional resistance protein PTR recognizes the *Magnaporthe oryzae* effector AVR-Pita in an allele-specific manner. *Nat. Plants* **10**, 994–1004 (2024).

41. W. Punt, J. Park, H. Roevenich, A. Kraege, N. Schmitz, J. Wieneke, N. C. Snelders, G. L. Fiorin, A. López-Moral, E. A. Chavarro-Carrero, G. C. Petti, K. Wippel, B. P. H. J. Thomma, A gnotobiotic system reveals multifunctional effector roles in plant-fungal pathogen dynamics. bioRxiv 645772 [Preprint] (2025). https://doi.org/10.1101/2025.03.27.645772.

42. S. Kumar, G. Stecher, M. Suleski, S. B. Hedges, TimeTree: A resource for timelines, timetrees, and divergence times. *Mol. Biol. Evol.* **34**, 1812–1819 (2017).

43. T. Y. James, F. Kauff, C. L. Schoch, P. B. Matheny, V. Hofstetter, C. J. Cox, G. Celio, C. Gueidan, E. Fraker, J. Miadlikowska, H. T. Lumbsch, A. Rauhut, V. Reeb, A. E. Arnold, A. Amtoft, J. E. Stajich, K. Hosaka, G. H. Sung, D. Johnson, B. O'Rourke, M. Crockett, M. Binder, J. M. Curtis, J. C. Slot, Z. Wang, A. W. Wilson, A. Schüßler, J. E. Longcore, K. O'Donnell, S. Mozley-Standridge, D. Porter, P. M. Letcher, M. J. Powell, J. W. Taylor, M. M. White, G. W. Griffith, D. R. Davies, R. A. Humber, J. B. Morton, J. Sugiyama, A. Y. Rossman, J. D. Rogers, D. H. Pfister, D. Hewitt, K. Hansen, S. Hambleton, R. A. Shoemaker, J. Kohlmeyer, B. Volkmann-Kohlmeyer, R. A. Spotts, M. Serdani, P. W. Crous, K. W. Hughes, K. Matsuura, E. Langer, G. Langer, W. A. Untereiner, R. Lücking, B. Büdel, D. M. Geiser, A. Aptroot, P. Diederich, I. Schmitt, M. Schultz, R. Yahr, D. S. Hibbett, F. Lutzoni, D. J. McLaughlin, J. W. Spatafora, R. Vilgalys, Reconstructing the early evolution of Fungi using a six-gene phylogeny. *Nature* **443**, 818–822 (2006).

44. X. Yuan, S. Xiao, T. N. Taylor, Lichen-like symbiosis 600 million years ago. *Science* **308**, 1017–1020 (2005).

45. M. A. Guerreiro, E. H. Stukenbrock, Fungal plant pathogens. *Curr. Biol.* **35**, R480–R484 (2025).

46. C. Y. Huang, K. Araujo, J. N. Sánchez, G. Kund, J. Trumble, C. Roper, K. E. Godfrey, H. Jin, A stable antimicrobial peptide with dual functions of treating and preventing citrus Huanglongbing. *Proc. Natl. Acad. Sci. U.S.A.* **118**, e2019628118 (2021).

47. D. Wu, L. Fu, W. Wen, N. Dong, The dual antimicrobial and immunomodulatory roles of host defense peptides and their applications in animal production. *J. Anim. Sci. Biotechnol.* **13**, 141 (2022).

48. R. Eichfeld, A. B. Endeshaw, M. J. Hellmann, B. M. Moerschbacher, A. Zuccaro, Domain gain or loss in fungal chitinases drives ecological specialization toward antagonism or immune suppression. bioRxiv 659886 [Preprint] (2025). https://doi.org/10.1101/2025.06.16.659886.

49. P. van Dam, L. Fokkens, S. M. Schmidt, J. H. J. Linmans, H. Corby Kistler, L. J. Ma, M. Rep, Effector profiles distinguish formae speciales of *Fusarium oxysporum*. *Environ. Microbiol.* **18**, 4087–4102 (2016).

50. F. Mesny, M. Bauer, J. Zhu, B. P. H. J. Thomma, Meddling with the microbiota: Fungal tricks to infect plant hosts. *Curr. Opin. Plant Biol.* **82**, 102622 (2024).

51. A. C. Sexton, B. J. Howlett, Parallels in fungal pathogenesis on plant and animal hosts. *Eukaryot. Cell* **5**, 1941–1949 (2006).

52. F. Teufel, J. J. Almagro Armenteros, A. R. Johansen, M. H. Gíslason, S. I. Pihl, K. D. Tsirigos, O. Winther, S. Brunak, G. von Heijne, H. Nielsen, SignalP 6.0 predicts all five types of signal peptides using protein language models. *Nat. Biotechnol.* **40**, 1023–1025 (2022).

53. P. K. Meher, T. K. Sahu, V. Saini, A. R. Rao, Predicting antimicrobial peptides with improved accuracy by incorporating the compositional, physico-chemical and structural features into Chou's general PseAAC. *Sci. Rep.* **7**, 1–12 (2017).

54. D. Veltri, U. Kamath, A. Shehu, Deep learning improves antimicrobial peptide recognition. *Bioinformatics* **34**, 2740–2747 (2018).

55. T.-T. Lin, L.-Y. Yang, I.-H. Lu, W.-C. Cheng, Z.-R. Hsu, S.-H. Chen, C.-Y. Lin, AI4AMP: An antimicrobial peptide predictor using physicochemical property-based encoding method and deep learning. *mSystems* **6**, e0029921 (2021).

56. H. Lee, S. Lee, I. Lee, H. Nam, AMP-BERT: Prediction of antimicrobial peptide function based on a BERT model. *Protein Sci.* **32**, e4529 (2023).

57. J. Yan, P. Bhadra, A. Li, P. Sethiya, L. Qin, H. K. Tai, K. H. Wong, S. W. I. Siu, Deep-AmPEP30: Improve short antimicrobial peptides prediction with deep learning. *Mol. Ther. Nucleic Acids* **20**, 882–894 (2020).

58. A. Bateman, M. J. Martin, S. Orchard, M. Magrane, R. Agivetova, S. Ahmad, E. Alpi, E. H. Bowler-Barnett, R. Britto, B. Bursteinas, H. Bye-A-Jee, R. Coetzee, A. Cukura, A. Da Silva, P. Denny, T. Dogan, T. G. Ebenezer, J. Fan, L. G. Castro, P. Garmiri, G. Georghiou, L.

Gonzales, E. Hatton-Ellis, A. Hussein, A. Ignatchenko, G. Insana, R. Ishtiaq, P. Jokinen, V. Joshi, D. Jyothi, A. Lock, R. Lopez, A. Luciani, J. Luo, Y. Lussi, A. MacDougall, F. Madeira, M. Mahmoudy, M. Menchi, A. Mishra, K. Moulang, A. Nightingale, C. S. Oliveira, S. Pundir, G. Qi, S. Raj, D. Rice, M. R. Lopez, R. Saidi, J. Sampson, T. Sawford, E. Speretta, E. Turner, N. Tyagi, P. Vasudev, V. Volynkin, K. Warner, X. Watkins, R. Zaru, H. Zellner, A. Bridge, S. Poux, N. Redaschi, L. Aimo, G. Argoud-Puy, A. Auchincloss, K. Axelsen, P. Bansal, D. Baratin, M. C. Blatter, J. Bolleman, E. Boutet, L. Breuza, C. Casals-Casas, E. de Castro, K. C. Echioukh, E. Coudert, B. Cuche, M. Doche, D. Dornevil, A. Estreicher, M. L. Famiglietti, M. Feuermann, E. Gasteiger, S. Gehant, V. Gerritsen, A. Gos, N. Gruaz-Gumowski, U. Hinz, C. Hulo, N. Hyka-Nouspikel, F. Jungo, G. Keller, A. Kerhornou, V. Lara, P. Le Mercier, D. Lieberherr, T. Lombardot, X. Martin, P. Masson, A. Morgat, T. B. Neto, S. Paesano, I. Pedruzzi, S. Pilbout, L. Pourcel, M. Pozzato, M. Pruess, C. Rivoire, C. Sigrist, K. Sonesson, A. Stutz, S. Sundaram, M. Tognolli, L. Verbregue, C. H. Wu, C. N. Arighi, L. Arminski, C. Chen, Y. Chen, J. S. Garavelli, H. Huang, K. Laiho, P. McGarvey, D. A. Natale, K. Ross, C. R. Vinayaka, Q. Wang, Y. Wang, L. S. Yeh, J. Zhang, UniProt: The universal protein knowledgebase in 2021. *Nucleic Acids Res.* **49**, D480–D489 (2021).

59. D. Osorio, P. Rondón-Villarreal, R. Torres, Peptides: A package for data mining of antimicrobial peptides. *R J.* **7**, 4–14 (2015).

60. J. Jumper, R. Evans, A. Pritzel, T. Green, M. Figurnov, O. Ronneberger, K. Tunyasuvunakool, R. Bates, A. Žídek, A. Potapenko, A. Bridgland, C. Meyer, S. A. A. Kohl, A. J. Ballard, A. Cowie, B. Romera-Paredes, S. Nikolov, R. Jain, J. Adler, T. Back, S. Petersen, D. Reiman, E. Clancy, M. Zielinski, M. Steinegger, M. Pacholska, T. Berghammer, S. Bodenstein, D. Silver, O. Vinyals, A. W. Senior, K. Kavukcuoglu, P. Kohli, D. Hassabis, Highly accurate protein structure prediction with AlphaFold. *Nature* **596**, 583–589 (2021).

61. P. J. A. Cock, T. Antao, J. T. Chang, B. A. Chapman, C. J. Cox, A. Dalke, I. Friedberg, T. Hamelryck, F. Kauff, B. Wilczynski, M. J. L. de Hoon, Biopython: Freely available Python tools for computational molecular biology and bioinformatics. *Bioinformatics* **25**, 1422–1423 (2009).

62. N. Mih, E. Brunk, K. Chen, E. Catoiu, A. Sastry, E. Kavvas, J. M. Monk, Z. Zhang, B. O. Palsson, ssbio: A Python framework for structural systems biology. *Bioinformatics* **34**, 2155–2157 (2018).

63. H. Chen, F. Gu, Z. Huang, Improved Chou-Fasman method for protein secondary structure prediction. *BMC Bioinformatics* **7**, 1–11 (2006).

64. R. Nagarajan, A. Archana, A. M. Thangakani, S. Jemimah, D. Velmurugan, M. M. Gromiha, PDBparam: Online resource for computing structural parameters of proteins. *Bioinform. Biol. Insights* **10**, 73–80 (2016).

65. W. Kabsch, C. Sander, Dictionary of protein secondary structure: Pattern recognition of hydrogen-bonded and geometrical features. *Biopolymers* **22**, 2577–2637 (1983).

66. W. G. Touw, C. Baakman, J. Black, T. A. H. Te Beek, E. Krieger, R. P. Joosten, G. Vriend, A series of PDB-related databanks for everyday needs. *Nucleic Acids Res.* **43**, D364–D368 (2015).

67. V. Le Guilloux, P. Schmidtke, P. Tuffery, Fpocket: An open source platform for ligand pocket detection. *BMC Bioinformatics* **10**, 1–11 (2009).

68. Y. Liang, S. Yang, L. Zheng, H. Wang, J. Zhou, S. Huang, L. Yang, Y. Zuo, Research progress of reduced amino acid alphabets in protein analysis and prediction. *Comput. Struct. Biotechnol. J.* **20**, 3503–3510 (2022).

69. J. L. Figueroa, A. Redinbo, A. Panyala, S. Colby, M. L. Friesen, L. Tiemann, R. A. White, MerCat2: A versatile k-mer counter and diversity estimator for database-independent property analysis obtained from omics data. *Bioinforma. Adv.* **4**, vbae061 (2024).

70. F. Pedregosa, G. Varoquaux, A. Gramfort, M. Vincent, B. Thirion, O. Grisel, M. Blondel, P. Prettenhofer, R. Weiss, V. Dubourg, J. Vanderplas, A. Passos, D. Cournapeau, M. Brucher, M. Perrot, É. Duchesnay, Scikit-learn: Machine learning in Python. *J. Mach. Learn. Res.* **12**, 2825–2830 (2011).

71. Z. Lin, H. Akin, R. Rao, B. Hie, Z. Zhu, W. Lu, N. Smetanin, R. Verkuil, O. Kabeli, Y. Shmueli, A. Dos Santos Costa, M. Fazel-Zarandi, T. Sercu, S. Candido, A. Rives, Evolutionary-scale prediction of atomic-level protein structure with a language model. *Science* **379**, 1123–1130 (2023).

72. R. de Jonge, H. P. Van Esse, K. Maruthachalam, M. D. Bolton, P. Santhanam, M. K. Saber, Z. Zhang, T. Usami, B. Lievens, K. V. Subbarao, B. P. H. J. Thomma, Tomato immune receptor Ve1 recognizes effector of multiple fungal pathogens uncovered by genome and RNA sequencing. *Proc. Natl. Acad. Sci. U.S.A.* **109**, 5110–5115 (2012).

73. P. J. Kersey, J. E. Allen, I. Armean, S. Boddu, B. J. Bolt, D. Carvalho-Silva, M. Christensen, P. Davis, L. J. Falin, C. Grabmueller, J. Humphrey, A. Kerhornou, J. Khobova, N. K. Aranganathan, N. Langridge, E. Lowy, M. D. McDowall, U. Maheswari, M. Nuhn, C. K. Ong, B. Overduin, M. Paulini, H. Pedro, E. Perry, G. Spudich, E. Tapanari, B. Walts, G. Williams, M. Tello–Ruiz, J. Stein, S. Wei, D. Ware, D. M. Bolser, K. L. Howe, E. Kulesha, D. Lawson, G. Maslen, D. M. Staines, Ensembl Genomes 2016: More genomes, more complexity. *Nucleic Acids Res.* **44**, D574–D580 (2016).

74. H. Muraguchi, K. Umezawa, M. Niikura, M. Yoshida, T. Kozaki, K. Ishii, K. Sakai, M. Shimizu, K. Nakahori, Y. Sakamoto, C. Choi, C. Y. Ngan, E. Lindquist, A. Lipzen, A. Tritt, S. Haridas, K. Barry, I. V. Grigoriev, P. J. Pukkila, Strand-specific RNA-seq analyses of fruiting body development in *Coprinopsis cinerea*. *PLOS ONE* **10**, e0141586 (2015).

75. I. V. Grigoriev, R. Nikitin, S. Haridas, A. Kuo, R. Ohm, R. Otillar, R. Riley, A. Salamov, X. Zhao, F. Korzeniewski, T. Smirnova, H. Nordberg, I. Dubchak, I. Shabalov, MycoCosm portal: Gearing up for 1000 fungal genomes. *Nucleic Acids Res.* **42**, D699–D704 (2014).

76. G. Yildirir, J. Sperschneider, M. Malar C, E. C. H. Chen, W. Iwasaki, C. Cornell, N. Corradi, Long reads and Hi-C sequencing illuminate the two-compartment genome of the model arbuscular mycorrhizal symbiont *Rhizophagus irregularis*. *New Phytol.* **233**, 1097–1107 (2022).

77. C. P. Cantalapiedra, A. Hernández-Plaza, I. Letunic, P. Bork, J. Huerta-Cepas, eggNOG-mapper v2: Functional annotation, orthology assignments, and domain prediction at the metagenomic scale. *Mol. Biol. Evol.* **38**, 5825–5829 (2021).

78. J. Huerta-Cepas, D. Szklarczyk, D. Heller, A. Hernández-Plaza, S. K. Forslund, H. Cook, D. R. Mende, I. Letunic, T. Rattei, L. J. Jensen, C. Von Mering, P. Bork, eggNOG 5.0: A hierarchical, functionally and phylogenetically annotated orthology resource based on 5090 organisms and 2502 viruses. *Nucleic Acids Res.* **47**, D309–D314 (2019).

79. J. Zheng, Q. Ge, Y. Yan, X. Zhang, L. Huang, Y. Yin, dbCAN3: Automated carbohydrate-active enzyme and substrate annotation. *Nucleic Acids Res.* **51**, W115–W121 (2023).

80. M. Bernhofer, B. Rost, TMbed: Transmembrane proteins predicted through language model embeddings. *BMC Bioinformatics* **23**, 1–19 (2022).

81. D. M. Emms, S. Kelly, OrthoFinder: Phylogenetic orthology inference for comparative genomics. *Genome Biol.* **20**, 1–14 (2019).

82. F. Mesny, S. Miyauchi, T. Thiergart, B. Pickel, L. Atanasova, M. Karlsson, B. Hüttel, K. W. Barry, S. Haridas, C. Chen, D. Bauer, W. Andreopoulos, J. Pangilinan, K. LaButti, R. Riley, A. Lipzen, A. Clum, E. Drula, B. Henrissat, A. Kohler, I. V. Grigoriev, F. M. Martin, S. Hacquard, Genetic determinants of endophytism in the *Arabidopsis* root mycobiome. *Nat. Commun.* **12**, 1–15 (2021).

83. D. M. Emms, S. Kelly, STAG: Species tree inference from all genes. bioRxiv 267914 [Preprint] (2018). https://doi.org/10.1101/267914.

84. F. Mesny, phylorep v0.1. *Zenodo* (2023). https://doi.org/10.5281/ZENODO.10142123.

85. M. N. Price, P. S. Dehal, A. P. Arkin, FastTree 2—Approximately maximum-likelihood trees for large alignments. *PLOS ONE* **5**, e9490 (2010).

86. G. L. Fiorin, A. Sanchéz-Vallet, D. P. d. T. Thomazella, P. F. V. do Prado, L. C. do Nascimento, A. V. d. O. Figueira, B. P. H. J. Thomma, G. A. G. Pereira, P. J. P. L. Teixeira,

Suppression of plant immunity by fungal chitinase-like effectors. *Curr. Biol.* **28**, 3023–3030.e5 (2018).

87. H. Tian, C. I. MacKenzie, L. Rodriguez-Moreno, G. C. M. van den Berg, H. Chen, J. J. Rudd, J. R. Mesters, B. P. H. J. Thomma, Three LysM effectors of *Zymoseptoria tritici* collectively disarm chitin-triggered plant immunity. *Mol. Plant Pathol.* **22**, 683–693 (2021).

88. C. A. Schneider, W. S. Rasband, K. W. Eliceiri, NIH Image to ImageJ: 25 years of image analysis. *Nat. Methods* **9**, 671–675 (2012).

89. B. Q. Minh, H. A. Schmidt, O. Chernomor, D. Schrempf, M. D. Woodhams, A. Von Haeseler, R. Lanfear, E. Teeling, IQ-TREE 2: New models and efficient methods for phylogenetic inference in the genomic era. *Mol. Biol. Evol.* **37**, 1530–1534 (2020).

90. K. Katoh, D. M. Standley, MAFFT multiple sequence alignment software version 7: Improvements in performance and usability. *Mol. Biol. Evol.* **30**, 772–780 (2013).

91. P. Jones, D. Binns, H. Y. Chang, M. Fraser, W. Li, C. McAnulla, H. McWilliam, J. Maslen, A. Mitchell, G. Nuka, S. Pesseat, A. F. Quinn, A. Sangrador-Vegas, M. Scheremetjew, S. Y. Yong, R. Lopez, S. Hunter, InterProScan 5: Genome-scale protein function classification. *Bioinformatics* **30**, 1236–1240 (2014).

92. I. Letunic, P. Bork, Interactive Tree of Life (iTOL) v6: Recent updates to the phylogenetic tree display and annotation tool. *Nucleic Acids Res.* **52**, W78–W82 (2024).

93. O. Emanuelsson, H. Nielsen, G. Von Heijne, ChloroP, a neural network-based method for predicting chloroplast transit peptides and their cleavage sites. *Protein Sci.* **8**, 978–984 (1999).

94. S. Kosugi, M. Hasebe, M. Tomita, H. Yanagawa, Systematic identification of cell cycle-dependent yeast nucleocytoplasmic shuttling proteins by prediction of composite motifs. *Proc. Natl. Acad. Sci. U.S.A.* **106**, 10171–10176 (2009).

95. W. Punt, A. Kraege, S. Metzger, N. Schmitz, J. Zhu, S. Hacquard, M. Bonkowski, N. C. Snelders, B. P. H. J. Thomma, Differential contributions of an antimicrobial effector from

Verticillium dahliae to virulence and tomato microbiota assembly across natural soils. Springer Nature, Microbiome (2026). 14:111. https://doi.org/10.1186/s40168-026-02376-y.

96. J. Li, L. Faino, G. L. Fiorin, S. Bashyal, A. Schaveling, C. van den Berg, M. F. Seidl, B. PHJ Thomma, B. Thomma, A single *Verticillium dahliae* effector determines pathogenicity on tomato by targeting auxin response factors. bioRxiv 517554 [Preprint] (2022). https://doi.org/10.1101/2022.11.22.517554.

97. L. Rehman, X. Su, H. Guo, X. Qi, H. Cheng, Protoplast transformation as a potential platform for exploring gene function in *Verticillium dahliae*. *BMC Biotechnol.* **16**, 57 (2016).

98. T. Leisen, F. Bietz, J. Werner, A. Wegner, U. Schaffrath, D. Scheuring, F. Willmund, A. Mosbach, G. Scalliet, M. Hahn, CRISPR/Cas with ribonucleoprotein complexes and transiently selected telomere vectors allows highly efficient marker-free and multiple genome editing in *Botrytis cinerea*. *PLOS Pathog.* **16**, e1008326 (2020).

99. X. Guo, X. Zhang, Y. Qin, Y. X. Liu, J. Zhang, N. Zhang, K. Wu, B. Qu, Z. He, X. Wang, X. Zhang, S. Hacquard, X. Fu, Y. Bai, Host-associated quantitative abundance profiling reveals the microbial load variation of root microbiome. *Plant Commun.* **1**, 100003 (2020).

100. J. M. Kremer, B. C. Paasch, D. Rhodes, C. Thireault, J. E. Froehlich, P. Schulze-Lefert, J. M. Tiedje, S. Y. He, FlowPot axenic plant growth system for microbiota research. bioRxiv 254953 [Preprint] (2018). https://doi.org/10.1101/254953.

101. B. Schlesier, F. Bréton, H. P. Mock, A hydroponic culture system for growing *Arabidopsis thaliana* plantlets under sterile conditions. *Plant Mol. Biol. Report.* **21**, 449–456 (2003).

102. E. A. Chavarro-Carrero, J. P. Vermeulen, D. E. Torres, T. Usami, H. J. Schouten, Y. Bai, M. F. Seidl, B. P. H. J. Thomma, Comparative genomics reveals the *in planta*-secreted *Verticillium dahliae* Av2 effector protein recognized in tomato plants that carry the V2 resistance locus. *Environ. Microbiol.* **23**, 1941–1958 (2021).

103. K. D. Curry, Q. Wang, M. G. Nute, A. Tyshaieva, E. Reeves, S. Soriano, Q. Wu, E. Graeber, P. Finzer, W. Mendling, T. Savidge, S. Villapol, A. Dilthey, T. J. Treangen, Emu: Species-

level microbial community profiling of full-length 16S rRNA Oxford Nanopore sequencing data. *Nat. Methods* **19**, 845–853 (2022).

104. P. Virtanen, R. Gommers, T. E. Oliphant, M. Haberland, T. Reddy, D. Cournapeau, E. Burovski, P. Peterson, W. Weckesser, J. Bright, S. J. van der Walt, M. Brett, J. Wilson, K. J. Millman, N. Mayorov, A. R. J. Nelson, E. Jones, R. Kern, E. Larson, C. J. Carey, İ. Polat, Y. Feng, E. W. Moore, J. VanderPlas, D. Laxalde, J. Perktold, R. Cimrman, I. Henriksen, E. A. Quintero, C. R. Harris, A. M. Archibald, A. H. Ribeiro, F. Pedregosa, P. van Mulbregt, SciPy 1.0 Contributors, SciPy 1.0: Fundamental algorithms for scientific computing in Python. *Nat. Methods* **17**, 261–272 (2020).

105. M. I. Love, W. Huber, S. Anders, Moderated estimation of fold change and dispersion for RNA-seq data with DESeq2. *Genome Biol.* **15**, 550 (2014).

106. F. Meyer, A. Fritz, Z.-L. Deng, D. Koslicki, T. R. Lesker, A. Gurevich, G. Robertson, M. Alser, D. Antipov, F. Beghini, D. Bertrand, J. J. Brito, C. T. Brown, J. Buchmann, A. Buluç, B. Chen, R. Chikhi, P. T. L. C. Clausen, A. Cristian, P. W. Dabrowski, A. E. Darling, R. Egan, E. Eskin, E. Georganas, E. Goltsman, M. A. Gray, L. H. Hansen, S. Hofmeyr, P. Huang, L. Irber, H. Jia, T. S. Jørgensen, S. D. Kieser, T. Klemetsen, A. Kola, M. Kolmogorov, A. Korobeynikov, J. Kwan, N. LaPierre, C. Lemaitre, C. Li, A. Limasset, F. Malcher-Miranda, S. Mangul, V. R. Marcelino, C. Marchet, P. Marijon, D. Meleshko, D. R. Mende, A. Milanese, N. Nagarajan, J. Nissen, S. Nurk, L. Oliker, L. Paoli, P. Peterlongo, V. C. Piro, J. S. Porter, S. Rasmussen, E. R. Rees, K. Reinert, B. Renard, E. M. Robertsen, G. L. Rosen, H.-J. Ruscheweyh, V. Sarwal, N. Segata, E. Seiler, L. Shi, F. Sun, S. Sunagawa, S. J. Sørensen, A. Thomas, C. Tong, M. Trajkovski, J. Tremblay, G. Uritskiy, R. Vicedomini, Z. Wang, Z. Wang, Z. Wang, A. Warren, N. P. Willassen, K. Yelick, R. You, G. Zeller, Z. Zhao, S. Zhu, J. Zhu, R. Garrido-Oter, P. Gastmeier, S. Hacquard, S. Häußler, A. Khaledi, F. Maechler, F. Mesny, S. Radutoiu, P. Schulze-Lefert, N. Smit, T. Strowig, A. Bremges, A. Sczyrba, A. C. McHardy, Critical Assessment of Metagenome Interpretation: The second round of challenges. *Nat. Methods* **19**, 429–440 (2022).

107. M. Kolmogorov, J. Yuan, Y. Lin, P. A. Pevzner, Assembly of long, error-prone reads using repeat graphs. *Nat. Biotechnol.* **37**, 540–546 (2019).

108. L. Gabriel, T. Brůna, K. J. Hoff, M. Ebel, A. Lomsadze, M. Borodovsky, M. Stanke, BRAKER3: Fully automated genome annotation using RNA-seq and protein evidence with GeneMark-ETP, AUGUSTUS, and TSEBRA. *Genome Res.* **34**, 769–777 (2024).

109. G. Yang, L. Tang, Y. Gong, J. Xie, Y. Fu, D. Jiang, G. Li, D. B. Collinge, W. Chen, J. Cheng, A cerato-platanin protein SsCP1 targets plant PR1 and contributes to virulence of *Sclerotinia sclerotiorum*. *New Phytol.* **217**, 739–755 (2018).

110. W. Zuo, J. R. L. Depotter, S. C. Stolze, H. Nakagami, G. Doehlemann, A transcriptional activator effector of *Ustilago maydis* regulates hyperplasia in maize during pathogen-induced tumor formation. *Nat. Commun.* **14**, 6722 (2023).

111. Y. Bai, D. B. Müller, G. Srinivas, R. Garrido-Oter, E. Potthoff, M. Rott, N. Dombrowski, P. C. Münch, S. Spaepen, M. Remus-Emsermann, B. Hüttel, A. C. McHardy, J. A. Vorholt, P. Schulze-Lefert, Functional overlap of the *Arabidopsis* leaf and root microbiota. *Nature* **528**, 364–369 (2015).

112. S. Seabold, J. Perktold, statsmodels: Econometric and statistical modeling with Python. *SciPy* **7**, 92–96 (2010).

113. S. Graves, H.-P. Piepho, L. Selzer, multcompView: Visualizations of paired comparisons. (2024). https://github.com/lselzer/multcompview.

114. C. L. Schoch, S. Ciufo, M. Domrachev, C. L. Hotton, S. Kannan, R. Khovanskaya, D. Leipe, R. McVeigh, K. O'Neill, B. Robbertse, S. Sharma, V. Soussov, J. P. Sullivan, L. Sun, S. Turner, I. Karsch-Mizrachi, NCBI Taxonomy: A comprehensive update on curation, resources and tools. *Database* **2020**, baaa062 (2020).

115. N. Istifadah, J. A. Saleeba, P. A. McGee, Isolates of endophytic *Chaetomium* spp. inhibit the fungal pathogen *Pyrenophora tritici-repentis* in vitro. *Can. J. Bot.* **84**, 1148–1155 (2006).

116. T. Sakamoto, J. M. Ortega, Taxallnomy: An extension of NCBI Taxonomy that produces a hierarchically complete taxonomic tree. *BMC Bioinformatics* **22**, 1–23 (2021).

117. G. Wang, X. Li, Z. Wang, APD3: The antimicrobial peptide database as a tool for research and education. *Nucleic Acids Res.* **44**, D1087–D1093 (2016).

118. L. V. Hedges, Distribution theory for Glass's estimator of effect size and related estimators. *J. Educ. Stat.* **6**, 107–128 (1981).
